# Land degradation is associated with larger crop yield gaps across global croplands

**DOI:** 10.1038/s43016-026-01382-5

**Published:** 2026-07-22

**Authors:** Hadi Hadi, D. K. Ray, P. Borrelli, J. S. Gerber, N. Shokri, S. Roman, D. Wuepper

**Affiliations:** 1 Land Economics Group, Institute for Food and Resource Economics, University of Bonn, Bonn, Germany; 2 Institute on the Environment, University of Minnesota, Saint Paul, MN USA; 3 Department of Environmental Sciences, Environmental Geosciences, University of Basel, Basel, Switzerland; 4 Department of Science, Roma Tre University, Rome, Italy; 5 Project Drawdown, Saint Paul, MN USA; 6 Institute of Geo-Hydroinformatics, Hamburg University of Technology, Hamburg, Germany; 7 United Nations University Hub on Engineering to Face Climate Change at the Hamburg University of Technology, United Nations University Institute for Water, Environment and Health, Hamburg, Germany

**Keywords:** Economics, Environmental impact, Environmental economics, Geography, Environmental studies

## Abstract

There is a long-standing concern that land degradation reduces agricultural productivity, particularly in the long run. However, the empirical evidence on this issue remains inconclusive. Here, we present a global quantification of the link between land degradation, measured as the difference between current and historical conditions, and crop yield gaps, defined as the difference between attainable and attained yields. Our analysis establishes that, on average, a 10% increase in land degradation is associated with a roughly 2% increase in average crop yield gaps. Regionally, the loss can reach up to 6% for a 10% increase in land degradation in the globally most affected hotspots. These include northern and southern India, northeastern China, the US Midwest, as well as parts of Central and South America, particularly northern Argentina. Aggregated across the world’s croplands, this translates into estimated losses of 20 million tonnes in crop production, corresponding to 52 trillion kcal, roughly the annual food calorie supply for 71 million people, and 4 billion USD in annual crop revenues.

## Abstract (web summary)

Land degradation is widely recognized as a threat to food security. Using high-resolution global data on multiple land degradation indicators and crop yield gaps, the study shows that greater historical land degradation is associated with larger yield gaps in major food-producing regions. These findings highlight the need for sustainable agricultural intensification and agri-environmental policies to reduce land degradation and protect future food production.

## Main

Land degradation, biodiversity loss and climate change are the three interconnected global grand challenges, as discussed by the three multilateral policy frameworks under the Rio Conventions (the United Nations (UN) Convention to Combat Desertification (UNCCD), the UN Framework Convention on Climate Change (UNFCC) and the UN Convention on Biodiversity (UNCBD)). Compared with climate change and loss of biodiversity, scientific understanding of land degradation is low^1^. This is particularly relevant to the grand challenge of ensuring food security through increasing crop yields^2,3^.

Despite the prevailing narratives that land degradation threatens food production^4^, there is still contrasting empirical evidence on whether land degradation, such as soil erosion, plays a negative role in cropland productivity^5^ and, if it does, by how much. Some studies report a small negative impact^5,6^, some report no impact^7^ and there are even studies reporting yield increases as a result of land degradation^6,8^.

However, results of small-scale field experiments cannot be directly extrapolated/generalized to the global scale, because the role of land degradation in crop yields varies across soils, climates, crops and management systems^6^. Moreover, land degradation encompasses more than just soil erosion; it also includes factors such as soil compaction, loss of soil organic carbon, depletion of soil water and reduction of aboveground vegetation cover^9–11^. Furthermore, plot experiments do not always accurately reflect real conditions on farmers’ fields. There is also the view that the importance of land degradation might be overestimated and farmers can actually compensate for losses in land quality by increasing fertilizer use, irrigation and other inputs^1,12^.

Compared with how climate change^13–15^ and agricultural management^16,17^ affect crop yields, there is currently a lack of systematic, global-scale evidence linking land degradation and crop yield gaps, and overall, it is unclear whether, globally, a negative link between land degradation and crop yields is detectable at all^5,6,12^. We hypothesize that more severe land degradation is associated with lower yields, which correspond to lower yield attainment relative to potentials—or, in other words, larger yield gaps.

Here we provide a spatially comprehensive, empirical quantification of the link between land degradation and crop yield gaps globally, based on high-resolution data and controlling for various confounding factors. Our study complements local, short-term, controlled field experiments, and prior global syntheses that relied on meta-analyses or global crop simulation models. Here, we leverage recent advances in global high-resolution inventory and mapping of land degradation^18,19^, crop yield gaps^20^ and agricultural inputs^21–25^.

Our outcome is global crop yield gaps, defined as the difference between observed yields and empirically attainable yields under real-world conditions^26^, rather than agronomic potential yields estimated under optimal conditions. Observed yields are derived from census and survey data, while attainable yields are based on the 95th percentile of observed yields, representing feasible yield ceilings achieved in practice under existing environmental, management and socio-economic constraints (Methods and Supplementary Note 2). This corresponds to ‘feasible’ yields in the sense of ref. ^27^, which is conceptually equivalent to the ‘plateau’ in farmer-attainable yields articulated by ref. ^26^. This approach enables a systematic comparison of productivity across locations while focusing on yield shortfalls relative to realistic, demonstrably attainable benchmarks.

Our land degradation measures are constructed as the deviation of current land conditions from historical baselines (‘debt’), following recent global assessments^18^. We consider multiple degradation processes—soil erosion^18^, compaction^19^, soil organic carbon loss^28^, soil water deficits^29^ and tree cover decline^18^—to capture key dimensions of long-term human-induced land change (Methods and Supplementary Note 3).

Our empirical framework estimates the association between land degradation and crop yield gaps at the global scale, quantifying the percentage change in yield gaps associated with a percentage change in different land degradation processes^30^. To account for interrelationships among degradation processes, all degradation indicators are included simultaneously. Beyond estimating the global average association, we also assess the spatial heterogeneity in these relationships using a machine learning approach^31,32^.

**Fig. 1:**
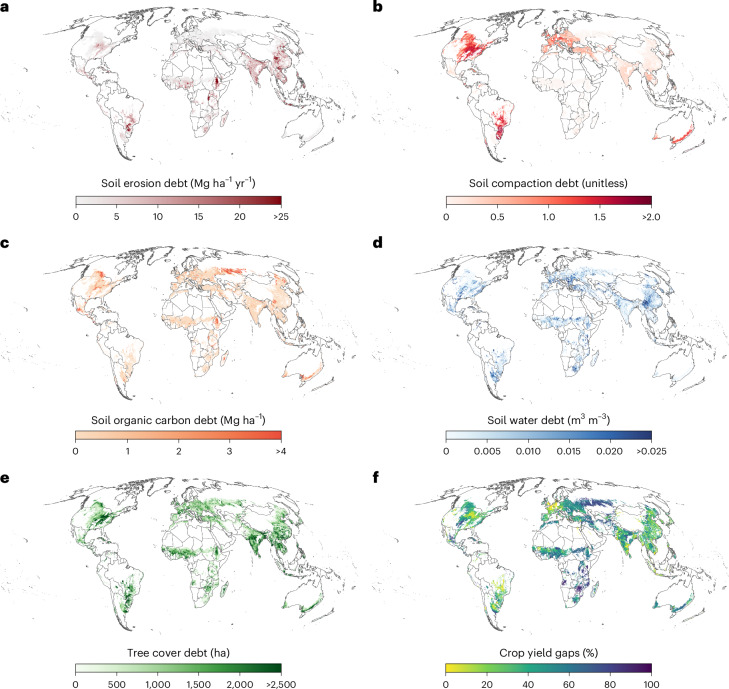
Land degradation and yield gaps. **a**–**e**, Global distributions of five land degradation measures, that is, soil erosion debt (**a**), soil compaction debt (**b**), soil organic carbon debt (**c**), soil water debt (**d**) and tree cover debt (**e**). **f**, Global crop yield gaps. For analysis, all data have been resampled to a common resolution of 5 arcmin (10 km), providing 405,084 observations (grid cells). We use average yield gaps^50^ (weighted by harvested area) for ten crops, namely, wheat, maize, rice, barley, sorghum, cassava, soybean, rapeseed, oil palm and sugar cane. All data represent circa 2010. We also use an extensive set of control variables covering natural–environmental factors (22 variables including climate, soil and terrain), agricultural inputs (10 variables including fertilizer, pesticide, irrigation, labour and machinery) and socio-economic variables (9 variables including agricultural share in gross domestic product, property rights and corruption) (Methods). The 97.5th percentile, 99th percentile and maximum values at grid cell level are 26.73, 42.09 and 162.85 Mg ha^−1^ yr^−1^ for soil erosion (rate) debt, 1.55, 1.63 and 2.00 for soil compaction debt; 4.90, 6.57 and 19.72 Mg ha^−1^ for soil organic carbon debt; 0.03, 0.04 and 0.23 m^3^ m^−3^ for soil water debt; 3,411, 4,013 and 6,789 ha for tree cover debt; and 82.52%, 89.41% and 99.55% for crop yield gaps. Basemaps generated with mapdata with data from the TM World Borders Dataset 0.3 (https://search.r-project.org/CRAN/refmans/prevR/html/TMWorldBorders.html) under a Creative Commons license CC BY SA 3.0. Source data

We examine high-resolution land degradation variables and crop yield gaps, as shown in Fig. 1. The empirical pattern shows that our measures of cropland degradation are a common by-product of intensive agriculture, especially when practised for a long time (Fig. 1a–e and Supplementary Fig. 1). This explains why there is commonly a positive association between land degradation and crop yields (Fig. 1f). This suggests that it is important to control for confounding factors, such as technology and management choices (fertilizer use and irrigation), as well as other sources of spatial heterogeneity (soils, climate and geography).

## Results

### Global average association of land degradation with crop yield gaps

**Fig. 2:**
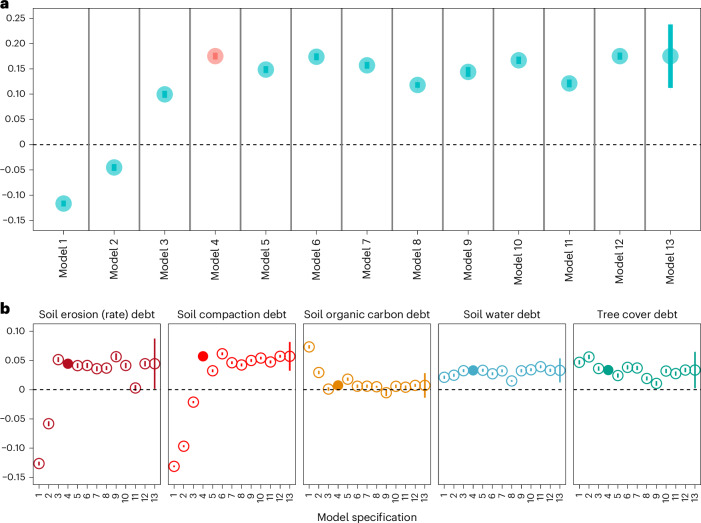
Land degradation is consistently associated with larger crop yield gaps. **a**, The overall association aggregated over all land degradation variables. **b**, The association by land degradation variable, that is, soil erosion (rate) debt, soil compaction debt, soil organic carbon debt, soil water debt and tree cover debt. Points indicate the marginal association as elasticities (Methods), that is, the per cent change in yield gaps associated with a 1% change in the land degradation measure (*n* = 405,084 grid cells). Error bars show the 95% confidence interval; the centre of each error bar is the point estimate. Exact two-sided *P* values are provided in the Source data. The *x* axis shows different model specifications. Model 1 is a regression with only the land degradation variables and crop yield gaps, without any controls. Model 2 includes natural–environmental controls. Model 3 includes additionally the management (agricultural inputs) controls. Model 4 (our preferred baseline specification, shown in red in **a** and as filled symbols in **b**) includes additionally the socio-economic–institutional controls. Model 5 includes additionally longitude and latitude spatial trends as controls. Model 6 is identical to model 4 but controls for crop types. Model 7 is identical to model 4 but omits gridded soil texture variables, which can be influenced by land degradation (that is, mediator variables). Model 8 is identical to model 4 but applies winsorization on the data at the 2.5th and 97.5th quantiles. Model 9 is identical to model 4 but adds quadratic terms of land degradation variables. Model 10 is identical to model 4 but adds all possible interaction terms between land degradation variables. Model 11 is identical to model 10 but adds all possible interaction terms between land degradation variables and the management (agricultural inputs) variables. Model 12 is identical to model 4 but with heteroscedasticity-robust standard errors (HC1). Model 13 is identical to model 4 but uses Conley standard errors with a 100-km spatial bandwidth. Results using the conservative Conley standard errors that take into account spatial autocorrelation for all models are shown in Supplementary Fig. 2. The spatial resolution of the analysis is 10 km. Source data

Our empirical analysis (Fig. 2a and Supplementary Table 1) shows that, after controlling for confounding factors, a significant positive association emerges between land degradation and crop yield gaps (that is, a negative association between land degradation and yield attainment). In other words, globally, on average, cropland areas with higher magnitude of historical land degradation, with other spatial heterogeneity controlled in the model being equal, correspond to areas presently having higher yield gaps. Our preferred model specification 4 includes controls for environmental, management (agricultural inputs) and socio-economic confounding factors. The association between crop yield gaps and the individual land degradation variables is that, for each 10% change in a land degradation variable, yield gaps change by 0.44% (95% confidence interval (CI) 0.40–0.49%; *P* < 0.01) for soil erosion debt, 0.57% (0.55–0.59%; *P* < 0.01) for soil compaction debt, 0.07% (0.05–0.10%; *P* < 0.01) for soil organic carbon debt, 0.33% (0.31–0.36%; *P* < 0.01) for soil water debt and 0.34% (0.30–0.37%; *P* < 0.01) for tree cover debt. To illustrate this magnitude, a 10% increase in land degradation corresponds to relatively modest changes in the underlying indicators when evaluated at their global means (Supplementary Table 2), ranging from 0.04 to 0.14 standard deviations across degradation dimensions. By comparison, heavily degraded grid cells (75–90th percentile of the global distribution) exhibit substantially higher degradation levels than the global median (for example, 3.4–6.9 times higher for soil erosion debt).

Overall, we estimate that, globally, on average, a 10% increase in overall land degradation is associated with a 1.75% (1.69–1.82%) increase in the average yield gap. The estimated relationship between land degradation and crop yield gaps remains significant with the alternative use of standard errors that take into account spatial autocorrelation (Supplementary Fig. 2).

Our estimates are robust across a wide range of alternative specifications (Fig. 2, Supplementary Figs. 2–5 and Supplementary Table 1). Across models, the overall association between land degradation and yield gaps remains consistently positive and statistically significant under alternative control variables, spatial adjustments and model functional forms (Supplementary Note 8). Figure 2 summarizes the main robustness checks, showing both the combined role across all land degradation indicators (Fig. 2a) and the individual contributions of each indicator (Fig. 2b). Across models (Fig. 2a, Supplementary Table 1 and Supplementary Fig. 3a), the associations between the overall land degradation and yield gaps are always significant and positive, with point estimates ranging between 1.18 and 1.88, meaning that a 1.18–1.88% increase in yield gaps is associated with a 10% increase in land degradation.

**Fig. 3:**
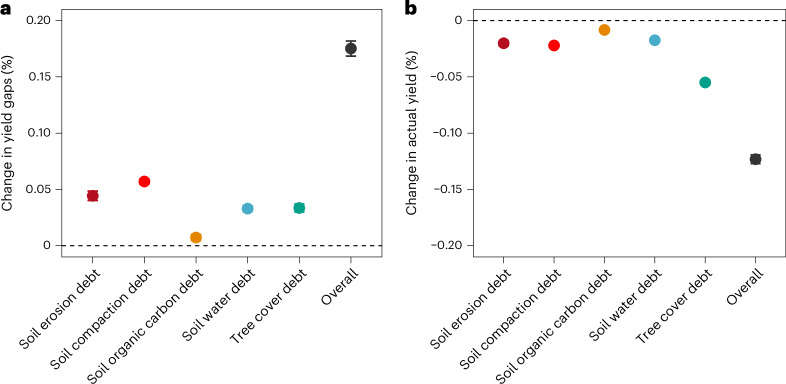
Land degradation and yield gaps and actual yields. **a**,**b**, The marginal association as elasticities (Methods), that is, the estimated mean percentage change in the yield gap (**a**) and actual yield (**b**) associated with a 1% increase in land degradation (*n* = 405,084 grid cells). Error bars show the 95% confidence interval; the centre of each error bar is the point estimate (mean elasticity from the regression). Exact two-sided *P* values are provided in the Source data. The results are based on model 4 (our preferred baseline specification; Fig. 2). Source data

Beyond examining yield gaps, we can also decompose the estimated elasticity by separately estimating the elasticity for actual yields and attainable yields (Fig. 3 and Supplementary Fig. 6). The estimated elasticity for the yield gap is +0.18, and this is indicated to be the result of an elasticity of −0.12 for the actual yield and −0.07 for the attainable yield; that is, the association is substantially more negative for actual yields (−0.12) than for attainable yields (−0.07), but both are affected.

### Spatial heterogeneity and country-level losses

**Fig. 4:**
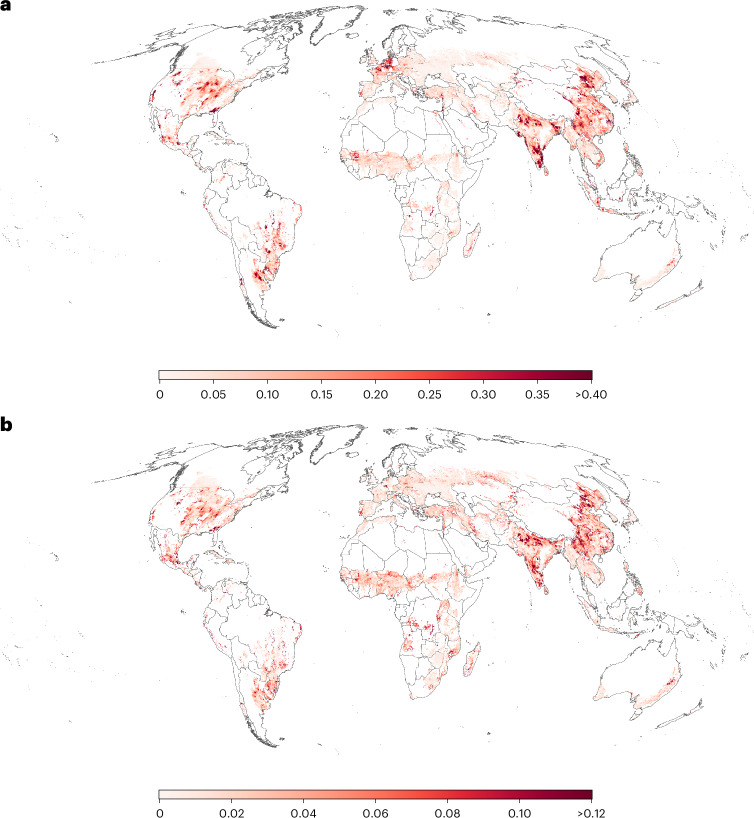
Estimated increase in crop yield gaps associated with overall land degradation. **a**, The marginal increase shown as elasticities, that is, the relative per cent (%) increase in yield gaps associated with a 1% increase in land degradation magnitude. **b**, The absolute percentage point (pp) increase in yield gaps associated with a 1% increase in land degradation magnitude, calculated by scaling the elasticity by current yield gap levels. The spatial resolution of the analysis is 10 km. The 97.5th percentile, 99th percentile, 99.9th percentile and maximum value at grid cell level is 0.44%, 0.62%, 1.10% and 2.27% for the relative change, and 0.13, 0.19, 0.38 and 1.45 pp for the absolute change. Maps of the elasticity estimates for each land degradation variable are shown in Supplementary Figs. 8–12. We test alternative causal forest models with additional hyperparameter tuning, and with feature selection for and fitting of the treatment (land degradation) and outcome (yield gaps) models separately, which result in similar overall spatial patterns of hotspots,in Supplementary Figs. 13 and 14. Basemaps generated with mapdata with data from the TM World Borders Dataset 0.3 (https://search.r-project.org/CRAN/refmans/prevR/html/TMWorldBorders.html) under a Creative Commons license CC BY SA 3.0. Source data

Beyond the global averages, the mapped heterogeneity of the land degradation–yield gaps association (Fig. 4a and Supplementary Fig. 7) obtained from the machine learning model (Methods and Supplementary Note 6) indicates that, at a local (grid-cell) level, a 10% increase in land degradation can correspond to yield gaps that are higher by up to 4.4–22.7% (97.5th, 99th, 99.9th and maximum of 4.4%, 6.2%, 11.0% and 22.7%). The map shows that the higher yield gaps (linked to higher land degradation) are particularly pronounced in India (northern and southern parts), China (dispersed throughout, but particularly the northeastern part), the USA (especially the Midwest^33^) and parts of Central and South America (northern part of Argentina). In terms of absolute percentage point (pp) increases in yield gaps, intensive farming regions in the USA exhibit moderate absolute pp increases, while regions in Europe show small absolute pp increases, reflecting their low baseline yield gaps (Fig. 4b). Regions in South Asia, China, and some parts of sub-Saharan Africa show comparable or higher absolute pp increases despite lower elasticities, driven by their higher baseline yield gaps. The hotspots of strong land degradation role in yield gaps are not as identifiable in sub-Saharan Africa.

Summed globally, the total losses (associated with a 1% increase in land degradation) are 2,002,307 tonnes yr^−1^ in crop production, 433,238,480 USD yr^−1^ in revenue (0.019% of global crop agricultural gross domestic product for 2010 of 2.3 trillion USD, based on data from ref. ^34^), 5,178,377 million kcal in food calories supply (corresponding to a loss of calories for 7,093,667 persons yr^−1^ assuming a 2,000 kcal daily requirement) and 132,800 tonnes yr^−1^ in food proteins supply (assuming a protein requirement of a 0.8 g per kg of body weight and 68.2 kg person, and so 55 g day^−1^, that is, protein for 6,615,203 persons year^−1^).

**Fig. 5:**
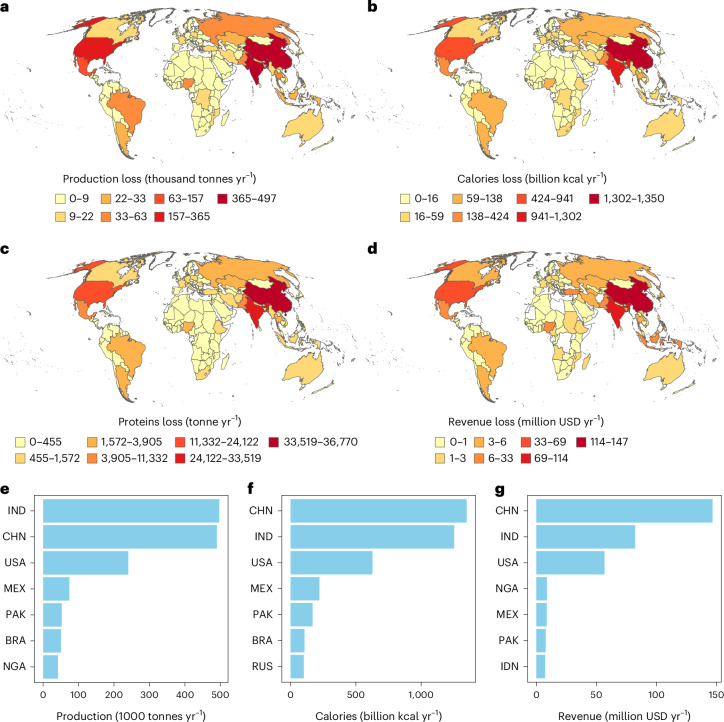
Country-aggregated estimated loss of production, calories, proteins and revenues associated with land degradation. **a**–**d**, Country-level estimates for all countries for loss of production (**a**), calories (**b**), proteins (**c**) and revenue (**d**). **e**–**g**, Values of loss of production (**e**), calories (**f**) and revenue (**g**) for the top seven countries with the largest losses. Values show the marginal loss associated with a 1% increase in land degradation magnitude. The country-level loss is the sum of grid cell-level losses (Supplementary Fig. 15) in each country. The map visualization uses seven classes determined by the Fisher–Jenks algorithm for natural breaks. IND, India; CHN, China; USA, United States of America; MEX, Mexico; PAK, Pakistan; BRA, Brazil; NGA, Nigeria; RUS, Russia; IDN, Indonesia. Basemaps in **a**–**d** generated with mapdata with data from the TM World Borders Dataset 0.3 (https://search.r-project.org/CRAN/refmans/prevR/html/TMWorldBorders.html) under a Creative Commons license CC BY SA 3.0. Source data

The highest absolute production losses (tonnes)—policy-relevant information within the context of global food supply—are estimated for India, China, USA, Mexico, Pakistan, Brazil, Nigeria, Russia, Thailand and Argentina (Fig. 5, Supplementary Table 3 and Supplementary Note 10).

**Fig. 6:**
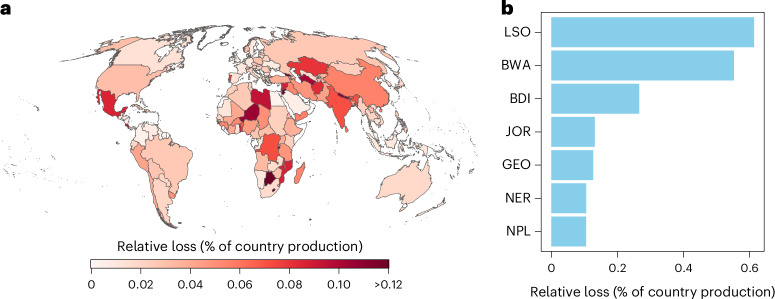
Country-aggregated relative production losses (per cent of total production) associated with land degradation. **a**, The relative production loss as a percentage of country-total production associated with a 1% increase in land degradation. The 97.5th percentile, 99th percentile, 99.9th percentile and maximum value at country level re 0.13, 0.45, 0.60 and 0.61. **b**, The top seven countries with the largest relative production loss associated with a 1% increase in land degradation. LSO, Lesotho; BWA, Botswana; BDI, Burundi; JOR, Jordan; GEO, Georgia; NER, Niger; NPL, Nepal. Basemap in **a** generated with mapdata with data from the TM World Borders Dataset 0.3 (https://search.r-project.org/CRAN/refmans/prevR/html/TMWorldBorders.html) under a Creative Commons license CC BY SA 3.0. Source data

In terms of relative losses (percentage of country-total production)—which identify countries most threatened relative to their domestic capacity, revealing where land degradation poses the greatest risk to local food supply and economic vulnerability—the highest losses (for a 1% increase in land degradation) are estimated for Lesotho (0.61%) and Botswana (0.55%), followed by Burundi (0.27%), Jordan (0.13%), Georgia (0.13%), Niger (0.11%) and Nepal (0.11%) (Fig. 6).

## Discussion

The conventional view of agricultural development often positions land degradation as primarily a developing-world problem. Our findings challenge this notion. Our results show that land degradation affects agricultural systems across all levels of economic development. That is, the overall spatial patterns of the degradation-associated yield losses suggest that, not only do richer countries experience land degradation just as much as the poorer, but also crop yields in richer countries with presumably greater resources to alleviate land degradation issues are just as much affected by land degradation. We find that higher degradation-associated yield losses occur predominantly in high-intensity agricultural systems with relatively small current yield gaps. This suggests that long histories of intensive agricultural practices in these high-yielding regions have created a highly optimized agricultural system that is particularly vulnerable to even a minor perturbation in soil conditions. This finding aligns with previous studies showing that high-yielding agricultural systems, while highly productive under optimal conditions, exhibit greater sensitivity to environmental stresses. For instance, ref. ^35^ found that US maize production has become increasingly sensitive to drought as farmers have removed non-water constraints. A previous high-resolution study also finds that crop yields in the high-yielding areas of the world are more sensitive to shocks in agricultural inputs^36^. These intensive systems have been optimized for maximum yields rather than resilience, making them particularly vulnerable to the marginal soil quality declines captured by land degradation debt.

In contrast, the results show that areas with high yield gaps found throughout Africa currently experience rather relatively low yield losses associated with historical land degradation. This pattern may be explained by the region’s historical reliance on extensive rather than intensive agricultural practices and the low use of agricultural inputs^37^. Moreover, African farming systems face multiple constraints beyond land degradation—such as limited access to fertilizers, irrigation, markets, farming knowledge and technology—that collectively contribute to high yield gaps^38,39^. Our findings indicate that, in this region with high yield gaps, addressing land degradation is probably of secondary priority for closing yield gaps, compared with the widely recognized need for input intensification^39^. Because intensive management has removed most other biophysical constraints in high-yielding regions, marginal soil degradation becomes a binding limitation—whereas, in low-input systems, yield gaps are linked to multiple unaddressed constraints, diminishing the relative signal of land degradation.

The local (grid cell) elasticity reveals which locations are most sensitive to land degradation, helping policymakers target restoration efforts where they will have the greatest proportional impact. The hotspots in Asia and Central and South America are particularly worrying as they might lead to cropland expansion in these regions, which have some of the largest remaining amounts of land suitable for agriculture^40^. Also, in these regions, the downstream impact of higher yield gaps (associated with higher land degradation) on human well-being is potentially the largest, owing to the lower income of the population, greater dependence on the agricultural sector, greater political instability and institutions that face challenges in effectiveness.

There are important differences in how absolute and relative losses should be interpreted. Countries with the highest absolute production losses combine substantial harvested areas with moderate-to-high attainable yields and sizeable absolute pp increases in yield gaps. In contrast, in countries with smaller production bases, moderate absolute production losses represent a substantial share of total production, indicating heightened economic vulnerability to land degradation despite lower absolute production losses. This divergence between the elasticity of yield gaps with respect to land degradation, absolute production losses and relative production losses reflects the interaction between yield sensitivity to land degradation, baseline yield gaps, production capacity and economic scale. Intensive farming systems demonstrate high marginal sensitivity to land degradation but benefit from low baseline yield gaps and high production volumes, resulting in small relative impacts. Conversely, countries with existing large yield gaps and smaller production bases face disproportionate relative production losses, even where the elasticity of yield gaps with respect to land degradation is more moderate. It is worth noting, however, that food produced within a country is not necessarily made available for domestic consumption, and therefore these country-aggregated figures should not be interpreted as contributing 1:1 to dietary requirements of the population within each respective country. Likewise, land degradation associated with agricultural activities, and in turn the adverse roles of land degradation on crop production and other ecosystem services, in one place is driven by both domestic and international crop production demands^40^.

Taken together, our findings have three key policy implications. Firstly, effective policy responses to close yield gaps must consider the different vulnerabilities of agricultural systems to land degradation. In regions with high yield gaps but low yield losses associated with land degradation—as shown in our results for Africa—policies should be tailored to facilitate agricultural intensification by improving access to agricultural inputs, markets and financial services. Secondly, such effective policies should be designed to avoid the pitfalls of unsustainably managed intensive agriculture. Our results indicate that past experiences from the world’s intensively farmed regions demonstrate how unsustainable practices have led to land degradation, ultimately undermining current productivity. Therefore, sustainable intensification should be designed to integrate conservation agriculture^41^ and integrated soil fertility management^42^ from the outset, ensuring that the promoted intensification enhances productivity while maintaining soil health, ultimately supporting long-term resilience and sustained agricultural output^43^. Thirdly, the global average association we found—a 2% increase in yield gaps associated with every 10% increase in land degradation—along with the resulting calorie and revenue losses, underscores that land degradation is a global economic and food security challenge. Given the interconnected nature of agricultural markets, mitigating the adverse effects of land degradation on food security and farmers’ livelihood requires stronger international cooperation and investment in soil restoration and conservation. This can include efforts to facilitate knowledge-sharing and financial incentives for sustainable land management practices and to develop a standardized global soil health monitoring system to guide effective and (spatially) targeted interventions.

Our estimates depend on the set of degradation processes and data sets currently available at global scale. Some important processes, such as salinization^44^ and nutrient imbalances, cannot yet be consistently quantified at high resolution or separated from natural background conditions and are therefore not explicitly included. In addition, soil nutrient mining (depletion), which is a land degradation process known to affect particularly the sub-Saharan Africa region^9^, is not accounted for in our analysis owing to the lack of suitable global-scale high-resolution data (and the ambiguity in defining nutrient imbalance as degradation, as both nutrient deficits and nutrient surpluses can have negative effects on crop yields, and as, while the former is more likely detrimental to yields, the latter can be considered a serious land degradation issue via off-site pollution). To the extent that these processes correlate with those analysed^9,45^, their effects may be partially captured in our estimates. Looking forward, as data become available, we highlight the need for further work incorporating other adaptation and response measures that may mask the role of land degradation in crop yields (such as conservation agriculture practices^41^), along with experimental methods, with the goal of establishing a causal relationship between land degradation and crop yields under diverse real-world management practices and environmental contexts.

In summary, our study provides empirical evidence that, on aggregate, historical land degradation already has a globally measurable role in current yield gaps. Furthermore, its negative role in yields is observed in major food-producing regions across both developing and developed countries. This underscores the critical need for globally concerted efforts to accelerate the adoption of sustainable agricultural intensification that can further mitigate potentially irreversible negative impacts of land degradation on food production. Agri-environmental policies^46^ that address land degradation therefore have the potential not only to mitigate its known negative environmental impacts but also to contribute to future food security.

## Methods

Our overarching goal is to identify how much historical land degradation plays a role in the global spatial pattern of current crop yield gaps. The yield gap is the difference between attained yield and estimated attainable yield for each location. Our analysis uses a cross-sectional design; that is, we exploit spatial differences in land degradation across grid cells. This approach is particularly appropriate for land degradation, which is a gradual, cumulative process not well captured by short-term temporal fluctuations. By leveraging cross-sectional variation, our analysis captures long-run equilibrium relationships between land degradation and agricultural yields.

When empirically estimating the association between the historical land degradation and current crop yield gaps (hereafter referred to as the degradation–yields association), we need to control for factors that may confound this association such as environmental (for example, climate, soil and terrain), agricultural management (for example, fertilizer and irrigation) and socio-economic variables (for example, income). The latter two—agricultural management and socio-economic variables—not only affect crop yields, but also capture the adaptation/adoption of compensatory measures with which farmers around the world have responded to changes in land conditions.

We compile the latest available high-resolution global data sets on crop yield gaps, land degradation and an extensive set of natural–environmental, agricultural management and socio-economic variables. Here, we rely on empirical crop yield data that are based on ground-based inventory (instead of a more indirect proxy via satellite remote sensing indicators) as reported in national and sub-national censuses and surveys around the world. Additionally, using yield gaps, instead of actual yields, provides a more nuanced understanding of differences in crop productivity between locations relative to location-specific yield potentials, therefore helping to isolate differences in crop productivity that are associated with land degradation as the focus of our study, along with agricultural management. We carry out the assessment using the latest available high-resolution global data sets of crop yield gaps compiled and aggregated for ten main crops for the year circa 2010^20^, along with a set of recently available high-resolution global land degradation data sets. The spatial resolution of our analysis is 10 km; that is, all variables are resampled to the 10-km crop yield gaps grid, which represents a compromise to preserve spatial detail in the high-resolution land degradation data while aligning to the coarser resolution data on the yield gaps outcome and agricultural inputs control variables. For all variables, when available, data for circa 2010 are used to match with the crop yield gaps data.

We are interested in the overall, long-term role of land degradation. Accordingly, our measures for land degradation are cumulative, constructed as differences between current (actual) land conditions and historical (natural) conditions, or as long-term differences in land conditions, based on available gridded data at global scale that capture variability in degradation conditions within croplands. For brevity, we refer to the differences as ‘debts’^18^, following previous research^18^. This does not mean that we assume that the historical land conditions are the most productive for agriculture (that is, is optimal agronomic condition). Rather, using such a historical benchmark provides us with a reference point to track changes and thus assess the role of long-term land degradation in current yield achievement.

To quantify the degradation–yields association, we first estimate the global average degradation–yields association using a multiple linear regression model with all the land degradation variables, and the natural–environmental, agricultural management and socio-economic control variables (Supplementary Tables 4 and 5). Following that, we estimate and map the heterogeneity of the degradation–yields association at 10 km $\times$ 10 km grid-cell level, using a causal machine learning method (causal forest^31,32^).

### Crop yield gaps data

Data on the global distribution of crop yield gaps is sourced from a recent comprehensive global assessment^20^ covering the ten most important global crops (wheat, maize, rice, barley, sorghum, cassava, soybean, rapeseed, oil palm and sugar cane), which together account for 83% of global calorie production^47^. Crop yield gaps are defined as the difference between current, that is, actually attained yields, and the attainable yields, expressed as a percentage relative to attainable yields. Observed yields were obtained from census and survey records^14^, whereas attainable yields were estimated empirically as the 95th percentile of observed yields under comparable biophysical conditions (Supplementary Note 2) representing realistic farmer-attainable (‘feasible’) yields^27^ rather than theoretical agronomic potential^26^. We acknowledge that, if land degradation is sufficiently widespread, even the 95th percentile may reflect somewhat degraded conditions, which would imply that our estimates of yield gaps—and consequently the role of land degradation—are conservative lower bounds. We use crop yield gap estimates for circa 2010 (mean of 2008–2012) to reduce sensitivity to short-term interannual variability, aggregated for all the ten crops using harvested-area weights.

### Land degradation data

Because land degradation manifests as different physical, chemical and biological processes, we assess several land degradation processes, that (1) are hypothesized to affect crop yields and have global footprints^9–11^, (2) for which high-resolution global data are available, (3) crucially, reflect spatial variability in land conditions within croplands and (4) allow for assessment of long-term human-induced changes. These land degradation processes include soil erosion (rate) debt^18^, soil compaction debt^19^, soil organic carbon debt (based on ref. ^28^), soil water debt (based on satellite-measured soil moisture^29^) and tree cover debt^18^ (Supplementary Note 3). While these five indicators certainly do not encompass all possible land degradation processes at any location across the globe, they represent key biophysical processes through which land degradation affects crop performance, including soil loss, fertility decline, soil structural deterioration and altered hydrological and microclimatic conditions (Supplementary Note 3). Unlike threshold-based approaches that classify degradation into binary or categorical classes^48^, we analyse land degradation as a continuous phenomenon (intensity/magnitude) to assess its association with crop yield gaps.

### Control variables

Estimating the association between land degradation and crop yield gaps at a global scale is challenged by confounding variables that affect both land degradation and crop yield gaps, potentially biasing the estimated association. Accounting for these confounders helps prevent the model from misattributing spatial variation in crop yield gaps driven by factors such as climate and management to land degradation. We therefore compile global gridded data sets of factors that affect both land degradation and crop yields, as well as factors that affect crop yields and improve the precision of the estimated degradation–yield association. These include climate, soil, terrain, agricultural inputs (including fertilizer^21^, irrigation^23^, pesticide^22^, labour and mechanization) and socio-economic and institutional indicators related to adaptive capacity (Supplementary Note 4).

### Estimating global and local degradation–yield associations

To quantify the global average association between land degradation and crop yield gaps, we estimate a multiple regression model relating crop yield gaps to the five land degradation variables while controlling for natural–environmental, agricultural management and socio-economic variables. We include all assessed land degradation processes simultaneously, considering that they may be interdependent and co-occur, which helps isolate the role of each degradation process while controlling for the others. To summarize the overall degradation–yield association across the investigated degradation dimensions, we also aggregate the estimated coefficients across the five land degradation variables (Supplementary Note 5).

We use a double-log (log–log) specification, such that the coefficients are interpreted as elasticities^30^, that is, the per cent change in crop yield gaps associated with a 1% change in the magnitude of the respective land degradation process, holding other factors constant. This allows a direct comparison of coefficients across land degradation variables. We also test alternative specifications including nonlinear terms and interactions (Supplementary Note 5).

The global average association may mask substantial regional heterogeneity. To estimate spatial heterogeneity in the degradation–yield association, we employ a causal machine learning method (causal forest^31,32^), which estimates grid cell-level conditional associations while flexibly adjusting for observed confounders (Supplementary Note 6). Because causal forest requires the specification of one treatment variable at a time, we run the model separately for each of the five land degradation variables while including the remaining degradation variables in the control set. For descriptive mapping of the overall spatial footprint of degradation-associated yield gaps, we similarly aggregate the grid cell-level estimated associations across the five degradation dimensions.

### Estimating loss of calories, proteins and revenues

We convert the estimated increase in yield gaps associated with land degradation into corresponding losses in crop production, food calories, protein supply and crop revenues. Because the yield gaps data represent a given year, these estimates reflect annual losses. Yield gap increases were converted into production losses using grid-cell attainable yields and harvested area, and then translated into calories, protein and revenues using country-level Food and Agriculture Organization Statistical Database food balance sheet and producer price data (Supplementary Note 7).

### Reporting summary

Further information on research design is available in the Nature Portfolio Reporting Summary linked to this article.

## Supplementary information

Supplementary Information Supplementary Notes 1–12, Fig. 1–19 and Tables 1–5.

Reporting Summary

Supplementary Data 1 Multiple-sheet Excel file. Statistical source data for supplementary figures and Supplementary Table 1. The workbook includes model estimates, standard errors, 95% confidence intervals, exact *P* values and sample sizes.

## Source data

Source Data Fig. 1 Map Source Data. Tabular source data for Fig. 1. The file contains longitude and latitude coordinates, five land degradation indicators, and crop yield gap values for the 405,084 10-km grid cells used in the analysis.

Source Data Fig. 2 Statistical Source Data. Statistical source data for Fig. 2. The workbook contains regression coefficient estimates, standard errors, 95% confidence intervals, exact *P* values and sample sizes for the overall land degradation coefficient plot and the land degradation indicator-specific coefficient plot.

Source Data Fig. 3 Statistical Source Data. Statistical source data for Fig. 3. The workbook contains regression coefficient estimates, standard errors, 95% confidence intervals, exact *P* values and sample sizes for models relating land degradation to crop yield gaps and actual yields.

Source Data Fig. 4 Map Source Data. Tabular source data for Fig. 4. The file contains grid-cell coordinates, the estimated relative change (%) in crop yield gaps, and the corresponding absolute change (percentage points) associated with a 1% increase in overall land degradation.

Source Data Fig. 5 Statistical Source Data. Country-level source data for Fig. 5. The workbook contains country-level estimates of production, calorie, protein and revenue losses for the map panels, and the plotted top-country values for the barplot panels.

Source Data Fig. 6 Statistical Source Data. Country-level source data for Fig. 6. The workbook contains country-level relative production loss estimates for the map panel and the plotted top seven countries for the barplot panel.

## Data Availability

All input data sets used in this study were obtained from publicly available sources and are described in the Methods and listed in Supplementary Table 4. The minimum data set required to interpret, verify and reproduce the findings of this study, together with processed data and full replication materials, is available via Zenodo at 10.5281/zenodo.20214047 (ref. ^49^). Source data are provided with this paper.

## Code availability

All custom code used for data processing, analysis and figure generation in this study (R scripts and Google Earth Engine workflows) is available via Zenodo at 10.5281/zenodo.20214047 (ref. ^49^). Analyses were performed using R (R Foundation for Statistical Computing), Google Earth Engine, and openly available software packages as described in the Methods.
