# Supplementary material for: Land degradation is associated with larger crop yield gaps across global croplands
Source: Nat Food. 2026 Jul 22;7(7):678–87. doi: 10.1038/s43016-026-01382-5 (PMC13391347; doi:10.1038/s43016-026-01382-5)
Supplement: Supplementary file 1 — Supplementary Notes 1–12, Fig. 1–19 and Tables 1–5. [file 43016_2026_1382_MOESM1_ESM.pdf]

nature food

Supplementary information https://doi.org/10.1038/s43016-026-01382-5

Land degradation is associated with larger crop yield gaps across global croplands

In the format provided by the authors and unedited

Table of Contents

This file includes:

Supplementary Notes (1-12)

Supplementary Figures (1-19)

Supplementary Tables (1-5)

Supplementary Notes

Supplementary Note 1 Study design and analytical framework

Supplementary Note 2 Yield-gap data and aggregation across crops

Supplementary Note 3 Land degradation indicators (“debt” metrics)

Supplementary Note 4 Control variables

Supplementary Note 5 Global average associations: multivariable regression

Supplementary Note 6 Spatial heterogeneity: causal forest estimation

Supplementary Note 7 Translating model estimates into production, calorie, protein, and revenue losses

Supplementary Note 8 Robustness and sensitivity analyses

Supplementary Note 9 Interpreting a 10% increase in land degradation

Supplementary Note 10 Country-level losses associated with land degradation

Supplementary Note 11 Comparison with prior global estimates

Supplementary Note 12 Limitations, data gaps, and contributions

Supplementary Figures

Supplementary Fig. 1 Map- of land degradation variables and crop yield gaps.

Supplementary Fig. 2 Estimated association between land degradation and crop yield gaps across different model specifications, using Conley standard errors.

Supplementary Fig. 3 Estimated association between land degradation and crop yield gaps across different additional regression model specifications for robustness checks.

Supplementary Fig. 4 Robustness check comparing baseline model coefficients with residualized land degradation specification.

Supplementary Fig. 5 Robustness check comparing baseline model coefficients across spatial resolutions.

Supplementary Fig. 6 Land degradation and attainable yields.

Supplementary Fig. 7 Estimated increase in crop yield gaps associated with overall land degradation.

Supplementary Fig. 8 Estimated increase in crop yield gaps associated with soil erosion debt.

Supplementary Fig. 9 Estimated increase in crop yield gaps associated with soil compaction debt.

Supplementary Fig. 10 Estimated increase in crop yield gaps associated with soil organic carbon debt.

Supplementary Fig. 11 Estimated increase in crop yield gaps associated with soil water debt.

Supplementary Fig. 12 Estimated increase in crop yield gaps associated with tree cover debt.

Supplementary Fig. 13 Estimated increase in crop yield gaps associated with soil erosion debt, with causal forest hyperparameters tuning (set to “all”).

Supplementary Fig. 14 Estimated increase in crop yield gaps associated with soil erosion debt, with feature selection using multivariate adaptive regression splines (MARS) in both the treatment and outcome model subroutines of causal forest.

Supplementary Fig. 15 Estimates of production, calorie, protein, and revenue losses due to overall land degradation.

Supplementary Fig. 16 Estimates of country-aggregated (sum) loss of production, calories, proteins, and revenues associated with overall land degradation.

Supplementary Fig. 17 Input data for the calculations to translate the estimated change in yield gaps associated with land degradation, into corresponding change in food production, calories supply, proteins supply, and agricultural revenues.

Supplementary Fig. 18 Yield gap elasticity to land degradation by dominant crop type.

Supplementary Fig. 19 Spatial distribution of soil erosion debt at multiple intensity levels relative to the global median.

Supplementary Tables

Supplementary Table 1 Summary of coefficients (elasticities) estimating the global association of overall land degradation to yield gaps, across different regression model specifications for robustness checks.

Supplementary Table 2 Interpretation of a 10% increase in land degradation indicators.

Supplementary Table 3 Country-level absolute losses associated with a 1% increase in land degradation for the 10 countries with the highest production losses.

Supplementary Table 4 Summary of data as used in the main estimation sample i.e., after resampling to 10-km common resolution, and filtering for grid cells with values for all variables available. N = 405,084.

Supplementary Table 5 Summary statistics of data as used in the main estimation sample i.e., after resampling to 10-km common resolution, and filtering for grid cells with values for all variables available. N = 405,084.

Supplementary Notes

This section provides additional detail on the datasets, variable construction, and analytical framework used in the main text.

Supplementary Note 1 Study design and analytical framework

Our analysis focuses on estimating the relationship between treatment variables (land degradation indicators) and the outcome variable (crop yield gaps), while controlling for variables that may confound this specific relationship. This differs from a purely predictive modelling exercise, which focuses on maximizing predictive accuracy of the outcome variable rather than estimating conditional associations.

The yield gap is defined as the difference between attained yield and estimated attainable yield (in %) for each location. Using yield gaps, rather than actual yields, provides a more nuanced understanding of differences in crop productivity between locations relative to location-specific yield potential, thereby helping isolate differences in productivity associated with land degradation.

Land degradation indicators are cumulative measures constructed as differences between current and historical or counterfactual land conditions ("debts")[1].

We set up our models to estimate the change in yield gaps (in %) in response to changes in different kinds of land degradation (in %)[2–4].

To account for potential interrelationships among degradation processes, we always include all degradation variables simultaneously in our analyses.

The assessment is conducted using global gridded datasets aligned to a common 10-km resolution. All variables were resampled to the 10-km crop yield gap grid, representing a compromise between preserving spatial detail in high-resolution degradation datasets and matching coarser global datasets for crop yields and agricultural inputs. Where possible, data for circa 2010 were used to align with the crop yield gap outcome.

To quantify the global average degradation–yield association, we estimate multiple linear regression models including all degradation indicators and control variables simultaneously. Following standard practices, we evaluate the robustness of our results through sensitivity analysis, assessing whether the association remains stable under alternative model specifications e.g., with different control variables and different model functional forms[5,6].

Beyond the global average association , to estimate local heterogeneity in the degradation–yield association, we use causal forest methods[7,8] at 10-km × 10-km grid-cell resolution.

In the following we describe the datasets and the statistical framework in further details. All analyses are performed in R statistical environment[9] using terra[10], raster[11], sf[12], fixest[13], modelsummary[14], marginaleffects[15], data.table[16] and tidyverse[17] libraries, Geospatial Data Abstraction Library[18], and Google Earth Engine[19].

Supplementary Note 2 Yield-gap data and aggregation across crops

Our outcome is global crop yield gaps, which are defined as the difference between observed yields in any given location and demonstrably attainable (i.e., observed in real-world conditions[20], not agronomic potential) yields of these crops in each location. Our observed yield data come from census and survey records and are therefore based on ground data collection rather than from a model. This ensures that real-world yield variability due to environmental factors, management practices, and socio-economic conditions, including the effect of land degradation, is captured. Our attainable yields are also based on observed yield ceiling, and thus represent what farmers actually achieve in the best-performing areas, given real-world environmental, management and socioeconomic constraints (such as institutions, market, input access, and farmer behavior). This contrasts with purely biophysical (agronomic) potential yields, which are typically estimated using crop models or measured in highly controlled on-station experiments under some optimal conditions (sometimes with water constraints, but always without management and socioeconomic constraints). Our objective, therefore, is to assess yield gaps relative to realistic attainable yield ceilings, as observed in actual farming conditions, rather than relative to theoretical maximum yields.

The global crop yield gaps dataset used in this study was sourced from a recent comprehensive assessment[21]. The dataset includes the ten most important global crops: wheat, maize, rice, barley, sorghum, cassava, soybean, rapeseed, oil palm, and sugar cane, which together represent the majority of global food production[22]. The crop yield gaps dataset was originally constructed using actual yield data compiled from census and survey records across approximately 20,000 political units worldwide, following the methods of ref. 23. Substantial efforts were made to quality-control and harmonize the census and survey data to account for potential errors from uneven data collection, boundary changes, misreporting, or incorrect tabulation. This database therefore represents the highest resolution (up to district level) available empirical yield data in a given country. The georeferenced yield data were then spatialized into 5 arc-minutes (i.e., 10-km × 10-km) gridded data to facilitate overlay with other data layers, such as with the gridded attainable yields, to derive the gridded yield gaps surface.

Attainable yields were estimated empirically using quantile regression as the 95th percentile of observed yields under comparable environmental conditions. This corresponds to ‘feasible’ yields in the sense of ref. 24 which is conceptually equivalent to the ‘plateau’ in farmer-attainable yields articulated by ref. 20. The quantile regression is carried out using as predictors the climate variables, irrigation fraction, soil variables (soil available water capacity, soil organic carbon, soil pH), and topography. As such, attainable yields are estimated by pooling data across locations that share similar measured biophysical conditions, ensuring the 95th percentile benchmark reflects performance under comparable local environments rather than arbitrary geographic regions.

Because variations in yield gaps reflect spatial differences in yield potential due to differences in environmental factors such as climate, soil, and terrain[25], and differences in on-farm management, using yield gaps, instead of current yields, helps to make yield differences across locations comparable[25]. This in turn helps to isolate spatial variations in yields that can be attributed to land degradation.

We focus on spatial variations in overall productivity across entire global croplands rather than crop-specific yields, highlighting the total agricultural output lost (associated with land degradation), which is a key concern for policymakers and global food security.

For this study, crop-specific yield gaps are aggregated across the ten crops using harvested-area weights to represent overall cropland productivity. We use yield gap estimates for circa 2010, corresponding to the average across 2008–2012, thereby reducing the influence of short-term annual yield fluctuations.

A potential limitation of this empirical benchmark is that if land degradation is sufficiently widespread, even the highest-performing observed yields may partially reflect degraded conditions. In that case, estimated yield gaps – and associated productivity losses attributable to land degradation – would be conservative lower-bound estimates.

Supplementary Note 3 Land degradation indicators ("debt" metrics)

Our land degradation measures are also constructed as differences, here defined as the difference between current land conditions and historical conditions. For simplicity, we refer to these differences as *debts*, following previous research[1]. This does not mean, we assume that the historical land conditions are the most productive for agriculture (i.e., is optimal agronomic condition). Rather, using such a historical benchmark provides us with a reference point to track changes and thus assess the role of long-term land degradation in current yield achievement. This is helpful, particularly in global assessments[26–29], as it makes the different dimensions of land degradation comparable.

We opt for objective, measurable criteria based on latest data and models with operational global applications, instead of qualitative assessment relying solely on expert opinions as performed in early efforts on global land degradation assessments[30,31]. Since land degradation manifests as different physical, chemical, and biological processes, we assess several land degradation processes, that (i) are hypothesized to affect crop yields and have global footprints[32–34], (ii) for which high-resolution global data is available, (iii) crucially, reflect spatial variability in land conditions within croplands, and (iv) allow for assessment of long-term human-induced changes.

These include soil erosion (rate) debt[1], soil compaction debt[35], soil organic carbon debt (based on Zhao et al.[36]), soil water debt (based on satellite-measured soil moisture[37–39]), and tree cover debt[1,40,41].

While these five indicators do not encompass all possible land degradation processes at any location across the globe – worldwide, multiple risks to soil health have been documented [32,42,43] such as heightened erosion, organic matter depletion, salinization, acidification, pollution, toxicity, biodiversity decline, nutrient imbalances, compaction, surface sealing, vegetation loss, and moisture loss – they represent the key biophysical dimensions of land degradation that are both globally measurable (globally representative data available) and most directly linked to agricultural productivity decline. **Soil erosion** and **SOC debt** directly reflect loss of fertile topsoil and depletion of soil organic matter, both of which are key determinants of soil productivity[44,45]. Soil loss via elevated erosion is widely regarded as the most important land degradation process – driven by agricultural management practices – leading to loss of soil organic matter (and thus

water-holding capacity), nutrients and biodiversity that negatively impact agricultural production [42,46–50], because of its effectively irreversible nature (it takes about 1000 years to replace a depth of 25 mm of soil[51]). Soil organic matter – of which SOC is the main component – modulates the physical and chemical attributes of soil by strengthening its structure and stability[52], thereby enhancing water retention[53] and nutrient cycling[26,45,47,54]. **Soil compaction** reduces soil porosity and root growth[55,56], which in turn constrains oxygen availability, as well as water and nutrient uptake[57–59]. **Soil water debt** represents long-term declines in plant-available soil moisture within the root zone which may be linked to groundwater decline[60,61] that negatively affect yields[62–65]. Soil water debt was included because long-term declines in plant-available soil moisture directly reduce ecosystem productivity and agricultural yields, making it a fundamental dimension of land degradation. It reflects persistent drying of the root zone, often linked to groundwater depletion, reduced infiltration, and changes in soil structure. Soil water debt captures cumulative moisture deficits that impair vegetation growth and soil function. **Tree cover debt** captures the loss of arboreal vegetation within and adjacent to agricultural landscapes. Although this indicator does not directly correspond to within-field crop mechanisms, we included it because it has a wide range of indirect effects on soil, water, and climate. It serves as a useful proxy for broader agro-ecological effects that are otherwise difficult to account for directly – such as soil protection, pollination support, microclimate regulation, and water management[40,66–68]. Higher tree debt thus signals higher cascading degradation, which indirectly undermines agricultural resilience and environmental resilience. Thus, taken together, the land degradation indicators we could include in our analysis represent the key biophysical processes through which land degradation affects crop performance, namely through soil loss, fertility decline, soil structural deterioration, and altered hydrological and microclimatic conditions.

Our land degradation measures, in multiple dimensions, along with their combined use, represent a significant advancement in quantifying land degradation in croplands. This is as compared to the commonly used vegetation index trends that serve as a more generic, indirect proxy for land conditions for all land cover types. In addition, unlike previous studies that assess multiple land degradation processes based on critical thresholds to derive binary (presence/absence) or multiple-severity-categories focused on identifying co-occurrences of the processes[43], here we analyze land degradation as a continuous phenomenon (intensity, magnitude) to assess how land degradation associates with crop yield gaps.

Data for soil erosion debt is obtained from ref. 1, which estimates the current soil erosion and the natural soil erosion (i.e., scenario without human land use) rates (Mg ha^{-1} yr^{-1}), using the Global Soil Erosion Modeling platform (GloSEM), a global operational Revised Universal Soil Loss Equation (RUSLE) based model. Briefly, the RUSLE spatially explicit framework models soil erosion by water (inter-rill and rill erosion processes) based on a driving force (erosivity of the climate, based on rainfall), a resistance term (erodibility of the soil, based on soil texture, organic matter, and coarse fragmentation) and other factors representing the farming choice, i.e., topographical conformation of the field, the soil conservation practices, and the cropping system (the Land Cover and Management Factor, or the C-factor). The GloSEM module addressing the Land Cover and Management Factor (C) for agricultural land was developed using data for 170 crops provided by FAOSTAT (www.fao.org/faostat). These crops were (i) categorized into fourteen groups based on their soil cover effectiveness and susceptibility to erosion, and (ii) mapped across 3,252 subnational administrative units to capture the diversity of farming systems and crop distributions. Typical crop rotations were defined using multiannual crop data spanning a ten-year period (2001–2012), while the spatial extent of cropland was derived from the

harmonized 250-m MODIS Land Cover Type product (MCD12Q1). The current (actual) soil erosion rate was estimated for the reference year 2012[69]. The natural (potential, native) soil erosion rate in turn was estimated using C-factor based on the potential no-human land cover, in turn based on the potential percent tree cover from ref. 29 augmented by the MODIS Vegetation Continuous Fields (VCF) product[70]. We calculate soil erosion debt (Mg ha^{-1} yr^{-1}) as current (actual) soil erosion rate minus native (potential) soil erosion rate, hence positive values mean debt, and higher values correspond to higher land degradation. The original soil erosion debt data is available at 1-km resolution, which we resample to 10-km common analysis grid using mean resampling.

Data for soil compaction debt is sourced from ref. 35, which provides a gridded map of subsoil compaction susceptibility index (SCSI) for arable land, computed as the ratio of expected soil stress by average tractor size to estimated soil strength. Soil stress is estimated based on country-level information on farm size distribution, mechanization level, and tractor density, in the absence of reliable (spatially downscaled) gridded data for these specific variables. The most recent tractor density data for each country from the Food and Agriculture Organization of the United Nations (FAO) and World Bank was used, of which in the data the most recent year with records is 2009. Soil strength (pre-compression stress) is estimated based on soil texture and climatic averaged soil moisture. The original SCSI data is available at 0.1 degrees (i.e., 11-km) resolution, which we resample to the 10-km common analysis grid using nearest neighbor resampling. The SCSI (unitless) is interpreted as soil compaction debt, with higher values corresponding to higher land degradation.

Data for soil organic carbon (SOC) debt is based on global annual gridded SOC maps[36]. The dataset represents topsoil (0-30cm) SOC stocks (Mg C ha^{-1}) in mineral soils annually from 1981 to 2018. The SOC stocks were estimated using a hybrid machine learning and process-based modelling approaches, combining digital soil mapping[71] and RothC model[72], along with ~15,000 in situ SOC sample data from 1975-1980 and ~43,000 in situ sample data from after 1980. Covariates used include crop class, cropping system class, manure inputs in cropland (annual), climate (annual), land use/land cover (annual), topography, net primary productivity (NPP; annual), normalized difference vegetation index (NDVI; annual), soil group and soil variables (soil clay content, soil depth). We calculate SOC debt (Mg C ha^{-1}) as the difference between SOC stock circa 1980 (average of 1981-1985) and circa 2010 (average of 2008-2012) i.e., SOC circa 1980 minus SOC circa 2010 is used to compute the debt (thus positive values mean debt, and higher values mean higher long-term SOC reduction, and therefore correspond to higher land degradation). The original data is available at 5-km resolution, which we resample into the 10-km common grid using mean resampling.

Data for soil water debt is based on the European Space Agency Climate Change Initiative (ESA CCI) Soil Moisture product[37–39] Version 8.1 Combined Passive and Active Sensors product. The satellites-measured data product provides daily soil moisture ($m^{-3}m^{-3}$) starting from 1979, at ~25-km resolution, which we resample to 10-km common grid using nearest neighbor resampling. From daily soil moisture, we calculate annual average soil moisture. We then calculate soil water debt ($m^{-3}m^{-3}$) as the difference between soil moisture circa 1980 (average of 1979-1983) and soil moisture circa 2010 (average of 2008-2012) i.e., soil moisture circa 1980 minus soil moisture circa 2010 (positive values mean debt, and higher values mean higher long-term reduction in soil water, and thus higher land degradation). We note, however, that soil moisture's effects on crop yields are particularly nuanced; while we herein interpret soil moisture loss as a form of degradation detrimental to crop yields – primarily through reduced plant-available water and impaired root

development[32] – we recognize that in certain exceptions, such as poorly drained or high-rainfall regions, excessive soil moisture can exacerbate waterlogging and restrict aeration, thereby harming productivity[42]. Globally, however, reductions in root-zone soil moisture storage predominate as the key degradative mechanism of interest in our assessment, after accounting for concurrent changes in weather i.e., precipitation, temperature (part of controls in our regression models) to isolate agricultural influence.

Data for tree cover debt (in hectares) is obtained from ref. 1. It is based on difference between current tree cover estimated using Landsat data[73] and the potential (natural) tree cover[29] i.e., potential tree cover minus current actual tree cover (positive values mean debt, and higher values mean higher land degradation). The potential percent tree cover was modelled using machine learning based on visually interpreted percent tree cover in protected areas as training data (~78,000 plots) and environmental covariates including climate, topography, and soil variables (soil organic carbon, soil sand content, soil depth to bedrock)[29]. The original tree cover debt data is available at 1-km resolution, which we resample to the 10-km common grid using sum resampling.

The unifying aim of our framework is to isolate human-induced land degradation by quantifying it as a series of 'debts'[1], defined as the gap between current land conditions and counterfactual baselines that would exist in the absence of human agricultural influences. We prefer this approach as it enables a more robust assessment of historical degradation processes than is typical in prior global studies, which often rely on arbitrary baseline years that do not account for regionally variable agricultural histories. Instead, we prioritize pre-degradation benchmarks where feasible, drawing on available globally consistent, high-resolution gridded datasets for counterfactual (potential, natural) soil erosion rates and tree cover. Counterfactual land conditions are derived based on potential natural vegetation predicted using a machine-learning model trained on land cover data from protected areas (assumed to reflect near-natural states), or based on historical land cover databases (going back to pre-agriculture times); the predicted natural land cover serves as input to erosion prediction model, with all other environmental drivers (climate, soil, lithology) held constant, thus yielding estimates of 'no-human' baselines. The human-induced 'soil erosion debt' is then calculated as the difference between these potential erosion rates and observed present-day rates. We fully acknowledge that data availability constrains full consistency across all degradation metrics' benchmarks we use. For subsoil compaction, the pre-degradation benchmark is the inherent soil strength (precompression stress) as a function of intrinsic and environmental factors – soil texture, bulk density, and matric suction (soil moisture) – explicitly excluding the mechanical stresses from modern agricultural machinery, with the Subsoil Compaction Susceptibility Index (SCSI) derived as the ratio of estimated machinery-induced soil stress to this baseline strength, thereby isolating the human agriculture-driven soil compaction as our 'soil compaction debt'. For soil water debt and SOC debt, we benchmark against satellite-derived soil moisture and model prediction of SOC for circa 1980 and quantify the decline to circa 2010, while controlling for concurrent changes in climate (precipitation, temperature) in our regression model to isolate agricultural influences. Unlike the other land degradation variables, the soil water debt and SOC debt represent a more recent history, and thus its estimated role in current yield gaps is underestimated. In sum, these benchmarks, while varying by metric due to data limitations, are deliberately selected to approximate pre- or minimally human-influenced states.

Supplementary Note 4 Control variables

Estimating the association between land degradation and crop yield gaps at global scale is challenged by the presence of confounding variables that affect both land degradation and crop yield gaps (i.e., variables that are common cause to both land degradation and crop yield gaps), which can lead to bias (in sign/direction, in magnitude/strength, or in both) in the estimated association (i.e., the estimates being not equal to their true value). Carefully accounting for the potential confounders helps to prevent the model from mis-attributing the spatial variation in crop yield gaps that is in reality driven by the spatial variations in the confounding variables (for example, climate and management), to the spatial variations in land degradation intensities. We therefore compile global gridded datasets of factors that affect both land degradation and crop yields, as well as factors that affect crop yields (which controlling for would improve the precision of the estimated degradation-yields association). We use gridded data, for circa 2010 (i.e., average of 2008-2012, unless otherwise specified) when available to better match the spatial and temporal resolution and coverage of the crop yield gaps and land degradation variables.

Natural environmental controls

To control for weather variation/climate condition (precipitation, air temperature, and solar radiation), we use the 1-km static climatology data from WorldClim V2.1[74], which is the average of climate conditions during the 1970-2000 period. We also control for current weather based on TerraClimate[75] monthly climate data record at 5-km resolution for circa 2010 (average of 2008-2012). In addition, we control for long-term difference (between circa 1980 and circa 2010) in the climate variables that may, alongside agricultural activities, have contributed to the long-term reduction (debt) in soil water availability and SOC. By doing so, we isolate the reduction in soil water and SOC that are caused by human agricultural activities. For static climatology, we control for annual precipitation total, precipitation during the wettest month, annual mean air temperature, mean temperature during the warmest quarter, maximum temperature during the warmest month, annual mean incoming solar radiation, and yearly aridity (ratio of evapotranspiration and precipitation). For current (circa 2010) weather and long-term weather difference, we control for annual precipitation total, annual minimum temperature, annual maximum temperature, and annual mean solar radiation. The climate variables are resampled to 10-km common grid using mean resampling.

We also control for terrain (topographical) variables, and basic, relatively fixed soil characteristics namely soil type and soil texture. Elevation data and the derived slope is taken from the 250-m Global Multi-resolution Terrain Elevation Data (GMTED) 2010[76], whereas terrain ruggedness at 90-m is available from Amatulli et al.[77]. The terrain variables are mean-resampled to the 10-km common analysis grid. As for soil type, we use the predicted global map of the United States Department of Agriculture (USDA) great soil group at 250-m resolution[78]. Soil sand, silt, and clay content are taken from SoilGrids 2.0[71], which we average across the 0-30 cm soil depth. Soil type is resampled to 10-km common grid using mode (majority) resampling, whereas soil sand, silt, and clay content are resampled using mean. SoilGrids 2.0 is a suite of global gridded dataset of various soil properties, which is produced using Digital Soil Mapping (machine learning) methodology calibrated with plot-level measurements from around the world as training database and various geospatial environmental covariates. We do not control for other soil characteristics than mentioned above, as the other soil properties might be affected by land degradation[45]. These soil properties which are affected by degradation, in turn may affect crop yields, and thus controlling for them would absorb away the degradation-yields association because they lie in the

middle of the relational pathway between land degradation and yields. Soil texture (i.e., relative composition of sand, silt, and clay particles) may be changed by soil erosion; we test excluding them from the set of environmental controls, and it has negligible effects on the result (i.e., the estimated degradation-yields association).

Agricultural management (input) controls

We control for agricultural inputs—that affect crop yields and are thus expected to partly mask the degradation-yields association—namely fertilizer, irrigation, pesticide, labor, and mechanization. To match the spatial and temporal scale and coverage of our degradation-yields analysis, we give preference to gridded datasets that are available for circa 2010. Data on nitrogen based fertilizer application rate (g N year^{-1} per grid cell) for circa 2010 are sourced from the annual global gridded 10-km dataset[79]. This spatial downscaling-based data product includes ammonium (NH_4) fertilizer, nitrate (NO_3) fertilizer, manure fertilizer, as well as NH_x and NO_y atmospheric deposition, all of which we use. Soil phosphorus (kg ha^{-1}) prediction for 2010 at 1-km resolution[80] is used, and resampled to 10-km using mean aggregation. For irrigation, we use the global gridded (10-km) data on area equipped for irrigation[81], recently updated including for the year 2010[82]. For pesticide input, we use the pesticide application rate (kg ha^{-1}) high estimate for 2015[83]. We sum the pesticide estimates across all pesticide types, for each grid cell. The data for soil phosphorus, and for pesticide, are based on machine learning model prediction using covariates including various soil variables; we test including and excluding them which has negligible effects on the result. For labor and mechanization input factors, in the absence of gridded data, country-level annual employment in agriculture (% of total employment; share of working age persons who are engaged in the agricultural sector to total employment) from World Bank[84] for circa 2010, and country-level annual gap-filled data on farm machinery measured in metric horsepower from USDA[85] for circa 2010 are used.

Socio-economic and institutional controls

Previous studies have found global evidence for the important role of institutions and policies, relative to natural geographical endowments, in shaping the global crop yields variability[86]. Institutions and policies (constraints, frictions, incentives) shape the broader (macro) scale environment and context within which farmers operate, driving economic behaviors and attitudes of farmers as economic agents[87], for example in their adoption of (investment in) adaptation measures to mitigate the adverse role of land degradation in crop yields. Therefore, to better capture the masking role of agricultural adaptation on the degradation-yields association, we include a set of socio-economic controls in our model specification.

We include as controls the gridded (10-km) sub-national Human Development Index (HDI) and Gross Domestic Product (GDP; total, and per capita) for circa 2010[88], country-level agricultural share of GDP from World Bank[89] for circa 2010, country-level access to electricity in rural area (% of rural population)[90] for circa 2010, country-level mobile cellular phone subscription (persons per 100 people)[91] for circa 2010, as well as perceived environmental policy enforcement (developed by the World Economic Forum)[92] and the Bayesian corruption index[93], taken from Wuepper et al.[94]. These socioeconomic, and institutional and governance quality indicators serve as proxy to the availability of and access to resources, education and technology (agricultural research and development, extension services) to mitigate the adverse role of land degradation in yields, such as through sustainable farming practices. We also add the country-level property right

protection index[94] as a proxy to land tenure security, which may influence farmers' decisions on making long-term investments in sustainable practices[95]. We do not include in the controls set country-level variables that have negligible effects on the result and that have missing data for important countries with significant amount of cropland areas.

Supplementary Note 5 Global average associations: multivariable regression

To quantify the global average association between land degradation and crop yield gaps, we estimate the following model:

$$
\begin{aligned}
\ln \text{crop yield gap}_i = \alpha + \boldsymbol{\beta}_{\textbf{land degradation}} \cdot \textbf{ln land degradation}_i + \\
\boldsymbol{\gamma} \cdot \mathbf{E}_i + \boldsymbol{\theta} \cdot \mathbf{A}_i + \boldsymbol{\lambda} \cdot \mathbf{I}_i + \epsilon_I = \\
\alpha + \beta_{\text{soil erosion debt}} \cdot \ln \text{soil erosion debt}_i + \\
\beta_{\text{soil compaction debt}} \cdot \ln \text{soil compaction debt}_i + \\
\beta_{\text{soil organic carbon debt}} \cdot \ln \text{soil organic carbon debt}_i + \\
\beta_{\text{soil water debt}} \cdot \ln \text{soil water debt}_i + \\
\beta_{\text{tree cover debt}} \cdot \ln \text{tree cover debt}_i + \\
\boldsymbol{\gamma} \cdot \mathbf{E}_i + \boldsymbol{\theta} \cdot \mathbf{A}_i + \boldsymbol{\lambda} \cdot \mathbf{I}_i + \epsilon_i
\end{aligned}
\tag{1}
$$

where i is the spatial unit of analysis i.e., every 10-km × 10-km grid cell of the world's cropland, and $\boldsymbol{\beta}$ are the coefficients of interest i.e., the measure of the association between each land degradation independent variable and the crop yield gaps response variable. α is a constant (intercept), **E** is a vector of natural environmental (climate, soil, topography) controls, **A** is a vector of agricultural management (agricultural inputs) controls, **I** is a vector of socio-economic and institutional controls, ϵ is the error term, and ln is natural logarithm. We include all the assessed land degradation processes in the model simultaneously, considering that the land degradation processes may be interdependent and collocate/co-occur. This helps to isolate the role of each land degradation process while controlling for the other degradation processes. We run a double-log (log-log) regression model to simplify the interpretation of the coefficients $\boldsymbol{\beta}$ in terms of elasticities i.e., percentage changes (similar to previous studies looking at crop yield elasticities but to climate variables[3,4]). That is, β is interpreted as the percent change (of its baseline value) in crop yield gaps associated with a 1% change (of its baseline value) in the magnitude of the respective land degradation process, holding other factors constant. (Note that this is a relative elasticity, capturing proportional changes around the observed values in the data, rather than deviations from an absolute or counterfactual "no-human" baseline as in the debt-based conceptualizations of land degradation.). In other words, coefficient $\beta = b$ means a 1% change in the magnitude of land degradation is associated with a b% change in crop yield gaps, on average (global average i.e., averaged across all units (grid cells) and thereby all levels of land degradation). The interpretation in terms of elasticities (percentage changes) makes the coefficients of the land degradation variables from the regression model comparable. All input variables are log-transformed as in ref. 5, which helps normalize variables with skewed distributions and linearize the relationship between the independent variables and the dependent variable. Finally, the cumulative, overall (total) role of all the land degradation processes is estimated by summing the coefficients $\boldsymbol{\beta}$, with the sum representing the total percent change in yield gaps associated with a 1% change in the magnitude of all the investigated land degradation processes simultaneously. The standard error

of the sum of the coefficients is calculated as square root of the sum of the variance of individual land degradation variables and the covariance between the land degradation variables.

As an alternative specification to the above linear model, we also test including quadratic terms of the land degradation variables, including all interactions between the land degradation variables (two variables or more), and including all interactions between land degradation variables and gridded management (agricultural input) variables. Subsequently we calculate the average marginal associations of the land degradation variables (average slopes of the fitted nonlinear degradation-yields dose-response function) using the marginaleffects[15] library in R.

Supplementary Note 6 Spatial heterogeneity: causal forest estimation

The global average association estimated above may mask significant regional differences in the degradation-yields association and does not reveal the maximum association that has occurred at individual locations. To estimate spatial heterogeneity in the degradation-yields association, we employ a machine learning based method namely causal forest[7,8], an adaptation of the popular random forest[96] predictive machine learning algorithm. Causal machine learning leverages the predictive capability of modern machine learning methods, with the adjustment that instead of predicting an outcome variable (such as crop yields), the objective is to predict the role of some treatment variable (land degradation in our case) on the outcome (crop yield gaps in our case). The algorithm estimates the marginal influence of small changes in the continuous treatment variable i.e., the local slope of the outcome with respect to the treatment intensity. For each observation, the forest uses its ‘nearest neighbors’ in the covariate space to estimate the treatment effect. The approach makes use of the full samples in the dataset to model how the association varies (i.e., is heterogeneous) continuously across the covariate space. This has advantages over traditional approach based on subgroup analysis with arbitrarily pre-defined grouping (e.g., geographic regions), that assumes constant association within each subgroup. Moreover, such subgroups analysis to identify spatial heterogeneity in the association, while straightforward (in practice done by estimating separate regression models for separate geographical regions), is deemed not appropriate in the context of this study given the geographical differences in spatial resolution of the source data we use, particularly the available spatial aggregation of the reported yield censuses and surveys from different countries. The tree-based learning algorithm also means causal forest is able to capture complex non-linear relationships, and interactions between variables, which is likely necessary when predicting heterogeneity in the association i.e., beyond the aggregated global average estimated in the previous section. Thus, it allows us to predict the degradation-yields association for each individual sample i.e., the 10-km × 10-km grid cell in our case. We use the map of the grid-cell level association to identify local hotspots where land degradation has had strong positive association with crop yield gaps. In other words, we are interested in where land degradation has been associated with higher yield gaps or reduced ability to close yield gaps.

We run causal forest with the same model specification as in Equation 1, using the grf library[97] in the R statistical environment[9]. The hyperparameters are kept at their default values (number of trees 2000, and honesty fraction 0.5). We perform some initial tests running causal forest with additional hyperparameter tuning (set to “all”), or with feature selection and model fitting separately for the treatment model (land degradation) and the outcome model (crop yield gaps), which has negligible effects on the result. Causal forests apply the so-called “honest” splitting approach and use out-of-bag predictions to help mitigate overfitting and reduce bias from confounding variables. The estimated association at grid-cell level is interpreted as percent yield

gaps change associated with a 1% change in the magnitude of the specified land degradation, within that grid cell. Causal forest requires specifying one treatment variable (i.e., one land degradation variable in our case) at a time. Therefore, we run causal forest five times, to specify each of the five land degradation processes as the treatment variable, while we include the other land degradation variables not specified as the treatment variable, in the controls set. We therefore obtain the map of grid-cell-level degradation-yields association, for each land degradation variable. We then sum the grid-cell-level association, per grid cell, to estimate the total magnitude of the role of all the investigated land degradation processes in crop yield gaps, within each grid cell.

Supplementary Note 7 Translating model estimates into production, calorie, protein, and revenue losses

We convert the estimated percent increase in yield gaps associated with a 1% increase in land degradation magnitude at grid-cell level, into the corresponding amounts of loss in terms of food calories, protein content, and crop revenues. Note that as the yield gaps data is for a given year, the estimated role of land degradation here is the loss in food supply and crop revenues, per year. To do this, we convert the percent increase in yield gaps into the loss in crop production in tonnes (t). We convert the elasticity (percent increase in yield gaps relative to the baseline yield gaps value) into absolute percentage point increase in yield gaps. As the yield gaps data is presented in percent relative to attainable yields (tonnes ha^{-1}), we multiply the percentage points increase in yield gaps, with attainable yields (tonnes ha^{-1}), which gives absolute decrease in yields in tonnes ha^{-1}. As the yield gaps data were aggregated (weighted average, with per-crop harvested area as weights) across the ten crops, we use also attainable yields (tonnes ha^{-1}) aggregated for the ten crops (weighted average, with per-crop harvested area as weights), per grid-cell. Afterwards, the decrease in yields in tonnes ha^{-1} is multiplied with total (sum) harvested area (ha) of the ten crops per grid-cell, which gives total loss in crop production in tonnes.

In addition to absolute losses, we calculate relative losses as a percentage of country-total baseline production. At the grid-cell level, we computed baseline production (tonnes) as the product of actual yield (tonnes ha^{-1}) and harvested area (ha) across the ten crops. Production losses (tonnes) are computed as the product of yield losses (tonnes ha^{-1}) and harvested area. We then aggregate both quantities to the country level by summing across all grid cells within each country. Relative losses were calculated as the ratio of country-total production losses to country-total baseline production, expressed as a percentage.

To convert the per grid cell loss in crop production in tonnes into loss in food energy supply in kilocalories, we use country-level statistics of per food (commodity) category 'food' quantity (tonnes; original unit in the data is 1000 tonnes) and 'food supply (kcal)' (kcal; original unit in the data is million kcal) from the FAOSTAT Food Balance Sheet (FBS) 2010-[98]. To minimize the number of countries with missing data, we take available data for each country and crop category, from the year that is closest to 2010. To match with the crops used in the crop yield gaps data, we include FBS food categories namely wheat and products, rice and products, barley and products, maize and products, sorghum and products, cassava and products, sugar cane, rape and mustardseed, soyabeans, and palm oil. To obtain the food kilocalories supply per food quantity (kcal $tonne^{-1}$), and aggregate across the crops, we sum the food kilocalories supply (kcal) across crops, and sum the food quantity (tonnes) across crops, and then divide the two sums. Multiplying the loss in crop production in tonnes (aggregated across crops), with this food kilocalories supply

per food quantity (kcal tonne^{-1}) gives the total loss in food kilocalories (kcal). We follow similar calculation to convert loss in crop production in tonnes to loss in food protein supply in tonnes, using per food category value of ‘food’ (1000 tonnes) quantity (tonnes) and ‘Protein supply quantity (tonnes)’ variables in the Food Balance Sheet. Note that the above calculation is identical to using "Food supply (kcal/capita/day)", "Food supply quantity (kg/capita/yr)", and "Protein supply quantity (g/capita/day)" in the FBS data.

To convert the per grid cell loss in crop production in tonnes to loss in crop revenue in USD, we use country-level statistics of per food (commodity) category value of ‘Producer Price (USD/tonne)’ from the FAOSTAT Producer Prices sheet[99]. To minimize the number of countries with missing data, we take available data for each country and crop category, separately for producer price and production, from the year that is closest to 2010. To match with the crops used in the crop yield gaps data, Producer Prices sheet crop categories included are barley, maize (corn), rice, sugar cane, wheat, sorghum, soya beans, rape or colza seed, cassava (fresh), and palm oil. The prices (USD tonne^{-1}) are aggregated across the crops by weighted average, with per crop category production quantity (tonnes) as weights. The per crop category production quantity (tonnes) is taken from the Food Balance Sheet variable “Production” (1000 t).

$$\text{Loss in production (tonnes)} = \text{Increase in yield gaps (pp)} \times \text{Attainable yields (tonnes ha}^{-1}) \times \text{Harvested area (ha)} \quad (2)$$

$$\text{Loss in calories (kcal)} = \text{Loss in production (tonnes)} \times \text{Kilocalories supply per quantity (kcal tonne}^{-1}) \quad (3)$$

$$\text{Loss in protein (tonnes)} = \text{Loss in production (tonnes)} \times \text{Protein supply per quantity (tonnes tonne}^{-1}) \quad (4)$$

$$\text{Loss in revenue (USD)} = \text{Loss in production (tonnes)} \times \text{Producer price (USD tonne}^{-1}) \quad (5)$$

Supplementary Note 8 Robustness and sensitivity analyses

Our conclusions remain consistent across various model specifications (**Fig. 2**, **Supplementary Table 1**, **Supplementary Fig. 2**, **Supplementary Fig. 3**). That is, the overall association of land degradation to yield gaps is found consistently positive and statistically significant across different models: First, considering that soil texture (i.e., relative composition of sand, silt, and clay particles) may be changed by soil erosion, we test excluding them from the set of environmental controls (**Model 7**). Soil characteristics other than soil type and texture are not controlled for as they might be affected by land degradation[45]. Second, we test including soil depth[100], which could be affected by soil erosion (i.e., as a mediator of soil erosion's role on yields), but could also moderate the adverse role of soil erosion on crop yields (**Model 15**). Third, we test including soil total nitrogen[71] which should also capture soil nitrogen natural availability (**Model 16**). Fourth, we add groundwater table depth estimated under natural equilibrium conditions without anthropogenic use[101] (**Model 17**). Fifth, we test including gridded data of predicted field size classes[102], which can act as a high-resolution proxy to degree of mechanization (**Model 14**). Sixth, as mechanization level was used to model soil compaction[35] included in our assessed land degradation processes, we test including and excluding mechanization[85] as control (**Model 20**). Seventh, we test including as controls the agriculture share of government expenditure and agriculture orientation index for government expenditure (ratio of expenditure to revenue)[103] as a proxy to public investment in agricultural research and development (**Model 21**), as well as the Economic Freedom Index[104,105] (**Model 22**). Eighth, we test adding road density[106] (**Model 18**) or travel time to cities[107] (**Model 19**) as a control, as a proxy for farmers' access to markets, agricultural inputs, and agricultural extension services[108]. Ninth, we test adding latitude and longitude (second-order polynomial, including linear, quadratic, and interaction terms) as controls, which helps to account for broad spatial trends and geographic patterns (and location specific) in unobserved (unmeasured) spatial heterogeneity[109] (**Model 5**). This approach absorbs spatially structured confounding and reduces residual spatial autocorrelation, which in turn helps mitigate bias in coefficient estimates arising from unmodelled spatial structure. Tenth, we test controlling for crop types (**Model 6**). Eleventh, we also test removing control variables with high multicollinearity (variance inflation factor > 10)[110], in which only climate variables (eight in total) are highly multicollinear. Omitting these controls, the coefficients for all land degradation variables remain highly significant ($p < 0.01$) and positive, consistent with the baseline specification, with an overall association estimated at 0.188 (0.181-0.194). Excluding these control variables however increases the risk of omitted variable bias, potentially leading to biased regression coefficients[110], and is thus not our preferred specification. Overall, the results of these robustness checks support the conclusion that our chosen set of control variables has largely accounted for spatial heterogeneity that could confound the relationship between land degradation and crop yield gaps.

Concerning the model functional forms, while we opt for a linear model in our preferred specification to estimate the global average association (**Model 4**), we also tested alternative approaches: including quadratic terms of the land degradation variables (**Model 9**), including all interaction terms between the land degradation variables (two variables or more; **Model 10**), and including all interaction terms between land degradation variables and the gridded management (agricultural inputs) variables (**Model 11**). We found that the average marginal role (average slope) of the land degradation variables in these non-linear model specifications are close to the ones from the linear model. Across models (**Fig. 2A**, **Supplementary Table 1**, **Supplementary Fig. 3A**), the associations between the overall land degradation and yield gaps are always significant

and positive, with point estimates ranging between 1.18 and 1.88, meaning 1.18-1.88 percent increase in yield gaps associated with a 10% increase in land degradation.

In addition to the alternative functional-form specifications, we conducted further robustness checks to evaluate key methodological assumptions. To address concerns that the estimated associations may partly reflect shared climatic, soil, and management inputs used in the construction of both yield-gap and land degradation datasets, we residualized the land degradation variables with respect to these controls and re-estimated the model. The resulting coefficients were broadly similar to those from the baseline specification, and remained statistically significant for most degradation indicators, suggesting that the main findings are not driven by mechanical correlations arising from overlapping model inputs (**Supplementary Fig. *4***). We also tested the sensitivity of the results to the spatial resolution of the analysis by repeating the estimation at coarser grid resolutions (50 km and 100 km). Across resolutions, the estimated coefficients remained stable in sign, magnitude, and statistical significance, indicating that the observed relationships are robust to the spatial harmonization procedure used in the baseline analysis (**Supplementary Fig. 5**).

Supplementary Note 9 Interpreting a 10% increase in land degradation

To illustrate the magnitude, a 10% increase in the land degradation, evaluated at the mean of the land degradation measures, is an increase of 0.45 Mg ha^{-1} yr^{-1} in soil erosion debt, 0.04 index point in soil compaction debt, 0.06 Mg ha^{-1} in soil organic carbon debt, 0.001 m^3 m^{-3} in soil water debt, and 129 ha in tree cover debt (**Supplementary Table 2**). For context, the median soil erosion debt is 1.61 Mg ha^{-1} yr^{-1}, while heavily degraded grid cells (e.g., 75th-90th percentile) exhibit debts of 5.41-11.07 Mg ha^{-1} yr^{-1}. Comparing a grid cell at the mean (4.54 Mg ha^{-1} yr^{-1}) to one at double the mean (9.08 Mg ha^{-1} yr^{-1}) represents a 100% increase. Similarly, heavily degraded grid cells (75th-90th percentile) show substantially elevated debts relative to the median across all other degradation dimensions: soil compaction (0.68-1.26 vs. 0.17 index points), soil organic carbon (0.30-2.12 vs. 0.00 Mg ha^{-1}), soil water (0.01-0.02 vs. 0.00 mm), and tree cover (1721.77-2421.34 vs. 1114.89 ha). Relative to the global spatial variability i.e., spatial standard deviation (SD) of each land degradation indicator, a 10% increase represents an increase of 0.05 SD for soil erosion debt, 0.08 SD for soil compaction debt, 0.04 SD for soil organic carbon debt, 0.07 SD for soil water debt, and 0.14 SD for tree cover debt.

Supplementary Note 10 Country-level losses associated with land degradation

The country-level estimated losses associated with a 1% increase in land degradation (**Supplementary Table 3**) are, for production loss, 497 thousand tonnes $year^{-1}$ for India, 490 thousand tonnes $year^{-1}$ for China, and 240 thousand tonnes $year^{-1}$ for the United States. These are followed by Mexico (74 thousand tonnes $year^{-1}$), Pakistan (53 thousand tonnes $year^{-1}$), Brazil (51 thousand tonnes $year^{-1}$), and Nigeria (42 thousand tonnes $year^{-1}$). For revenues, the total losses are estimated at 147 million USD $year^{-1}$ for China, 82 million USD $year^{-1}$ for India, and 57 million USD $year^{-1}$ for the United States. These are followed by Nigeria (8.8 million USD $year^{-1}$), Mexico (8.7 million USD $year^{-1}$), Pakistan (7.7 million USD $year^{-1}$), and Indonesia (7.3 million USD $year^{-1}$). For food energy supply, the estimated losses are 1350 billion kcal $year^{-1}$ (to contextualize, assuming 2000 kcal $person^{-1}$ day^{-1}, 1,849,000 persons $year^{-1}$) for China, 1254 billion kcal $year^{-1}$ (1,717,000 persons $year^{-1}$) for India, and 628 billion kcal $year^{-1}$ (860,000 persons $year^{-1}$) for the

United States. These are followed by Mexico (221 billion kcal year^{-1}), Pakistan (169 billion kcal year^{-1}), Brazil (107 billion kcal year^{-1}), and Russia (101 billion kcal year^{-1}).

Supplementary Note 11 Comparison with prior global estimates

Our findings can be compared to previous studies which have a global scope, conducted at a relatively high spatial resolution, and which similarly assess long term impacts of land degradation on crop yields. Based on a review of relevant studies (mostly field experiments) from around the world that estimate agricultural productivity loss due to soil erosion, ref. 111 assumed a crop productivity loss of 8% in agricultural fields that have been intensively cultivated during the past 25-30 years and which experience severe soil erosion rates (> 11 Mg ha^{-1} yr^{-1}). Ref. 112 used a global gridded crop simulation model in which soil erosion is modelled based on assumed soil conservation practices (in turn based on terrain slope) and soil erosion impact on yields is modelled via the loss of topsoil nutrient (soil organic carbon, fertilizer, phosphorus). They obtained a global reduction of yields of 6% (averaged across all field management scenarios and the last 10-year period simulated) in areas with that same rate of severe soil erosion, for maize and wheat. Evaluated at the level of soil erosion of 2 Mg ha^{-1} yr^{-1} below which field studies show negligible effects on crop productivity[113], the corresponding elasticity is 0.018 (i.e., 8% / (100 × 9 Mg ha^{-1} yr^{-1} / 2 Mg ha^{-1} yr^{-1})%) and 0.013 for those two global average estimates respectively. The estimated elasticity for soil erosion debt in our study (0.044, **Fig. 2**; 0.075 when running our model with only soil erosion debt and not the other land degradation variables) is therefore 2.4 to 5.8 times larger than the available previous global estimates. Our combined estimate for all land degradation processes assessed in this study (0.175, **Fig. 2**, **Supplementary Table 1**) is in turn up to 13.5 times larger than the previously reported estimates for soil erosion impact.

Supplementary Note 12 Limitations, data gaps, and contributions

We address in our study the global link between land degradation and crop yield gaps. Our estimate of the global average degradation-yields association, and its spatial heterogeneity, naturally are dependent on the analyzed land degradation processes, and datasets which reflect the current state of knowledge on the locations and degrees of the degradation processes. We recognize that the land degradation processes analyzed in this present study are not an exhaustive account of all processes that may occur at any given location. Some of these processes are difficult to account for using the current debt approach. For example, soil salinization is a critical degradation process in several regions. However, to our knowledge, they cannot yet be quantified globally in a way that effectively isolates human-induced components from natural background levels. Existing global salinity datasets do not yet distinguish between primary salinization, naturally occurring due to parent material or climatic conditions, and secondary or human-induced salinization[114]. Similarly, analyzing nutrient balances would require data on soil nutrient stocks, nutrient inputs (or applications), and nutrient outputs (or harvests), which are not currently available at high resolution or for recent time periods. In addition, it is also important to note that there is currently no universal consensus on the global extent and severity of land degradation[33,34,115–117]. While we are unable to explicitly include certain land degradation processes (e.g., those related to soil biodiversity and nutrient availability) in this study, to the extent that they are interrelated with the processes we have analyzed[32,45,115,118,119], our primary estimate of the overall impact of land degradation likely encompasses some of the effects of these unaccounted processes. Looking

forward, as data become available, we highlight the need for further work incorporating other adaptation and response measures that may mask the role of land degradation in crop yields (such as conservation agriculture practices[120]), along with experimental methods, with the goal of establishing a causal relationship between land degradation and crop yields under diverse real-world management practices and environmental contexts. Finally, our study provides a globally comprehensive, broad assessment of the role of land degradation in overall cropland productivity. This serves a different purpose than studies aiming to provide localized insights at selected locations, which may need to account for crop specificities as well as precise location-specific agricultural practices and yield measurements. Our findings help identify priority areas for spatial targeting, where addressing land degradation (e.g., by conservation agriculture practices) can yield the greatest benefits for agricultural productivity, alongside the environmental and economic co-benefits[121].

Supplementary Figures

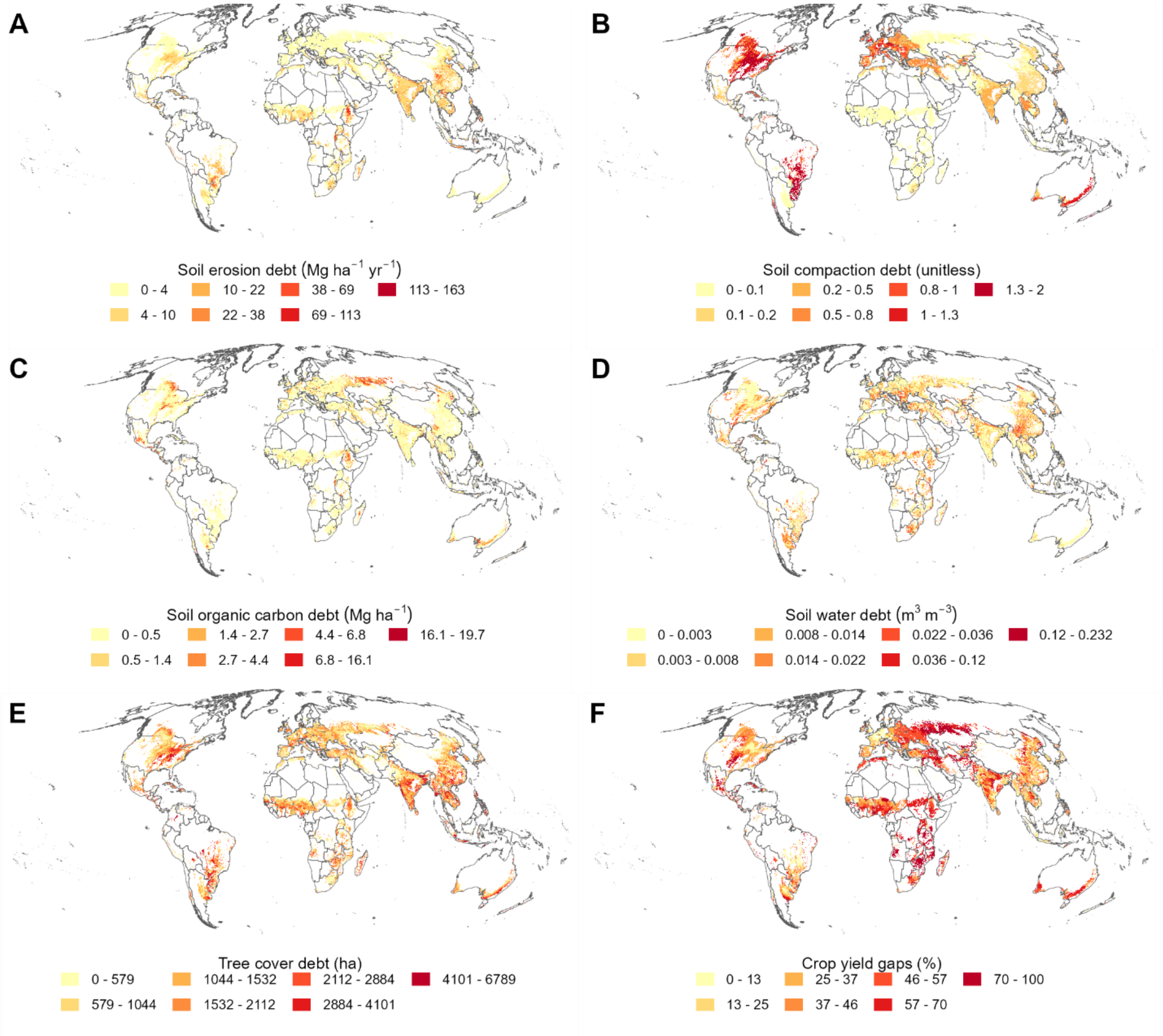

Supplementary Fig. 1 Map- of land degradation variables and crop yield gaps. The spatial resolution of the data is 10 km. The visualization uses seven classes determined by the Fisher-Jenks algorithm for natural breaks. The base map is from the mapdata package (TM World Borders Dataset 0.3) in R (https://search.r-project.org/CRAN/refmans/prevR/html/TMWorldBorders.html).

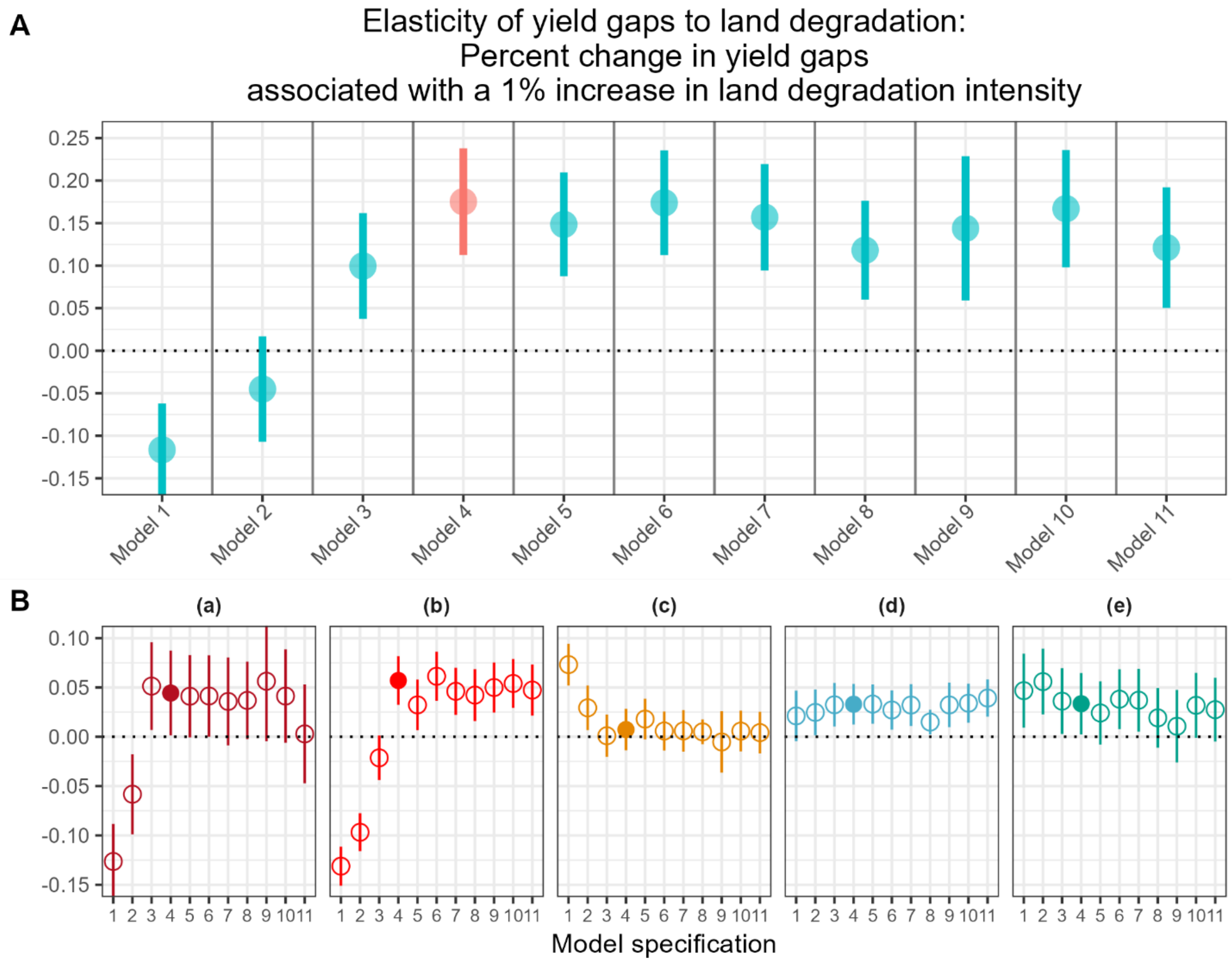

Supplementary Fig. 2 Estimated association between land degradation and crop yield gaps across different model specifications, using Conley standard errors. The spatial bandwidth is 100 km**.** Panel **A** shows the overall association for all land degradation variables combined. Panel **B** shows the association for each land degradation variable, i.e., soil erosion debt **(a)**, soil compaction debt **(b)**, soil organic carbon debt **(c)**, soil water debt **(d)**, and tree cover debt **(e)**. Points indicate the marginal association as elasticities (see the Methods section), i.e., the estimated mean percent (%) change in yield gaps associated with a 1% change in the land degradation measure (n = 405,084 grid cells). Error bars show the 95% confidence interval; the center of each error bar is the point estimate (mean elasticity from the regression). Exact two-sided p-values are provided in the Source Data file (Supplementary Data 1). **Model 1** is a regression with only the land degradation variables and crop yield gaps, without any controls. **Model 2** includes natural-environmental controls (**see the full list of control variables in Supplementary Table 4**). **Model 3** includes additionally the management (agricultural inputs) controls. **Model 4** (our preferred baseline specification) includes additionally the socio-economic-institutional controls. **Model 5** includes additionally longitude and latitude spatial trends as controls. **Model 6** is identical to Model 4 but additionally includes crop type dummies as controls. **Model 7** is identical to Model 4, but omits gridded soil texture variables, which can be influenced by land degradation (i.e., mediator variables), from the controls set. **Model 8** is identical to Model 4 but applies winsorization on the data at the 2.5th and 97.5th quantiles. **Model 9** is identical to Model 4 but adds quadratic terms of land degradation variables. **Model 10** is identical to Model 4 but adds all possible interaction terms between land degradation variables (between two or more variables). **Model 11** is identical to Model 10 but adds all possible interaction terms between land degradation variables and the management (agricultural inputs) variables. The spatial resolution of the analysis is 10 km.

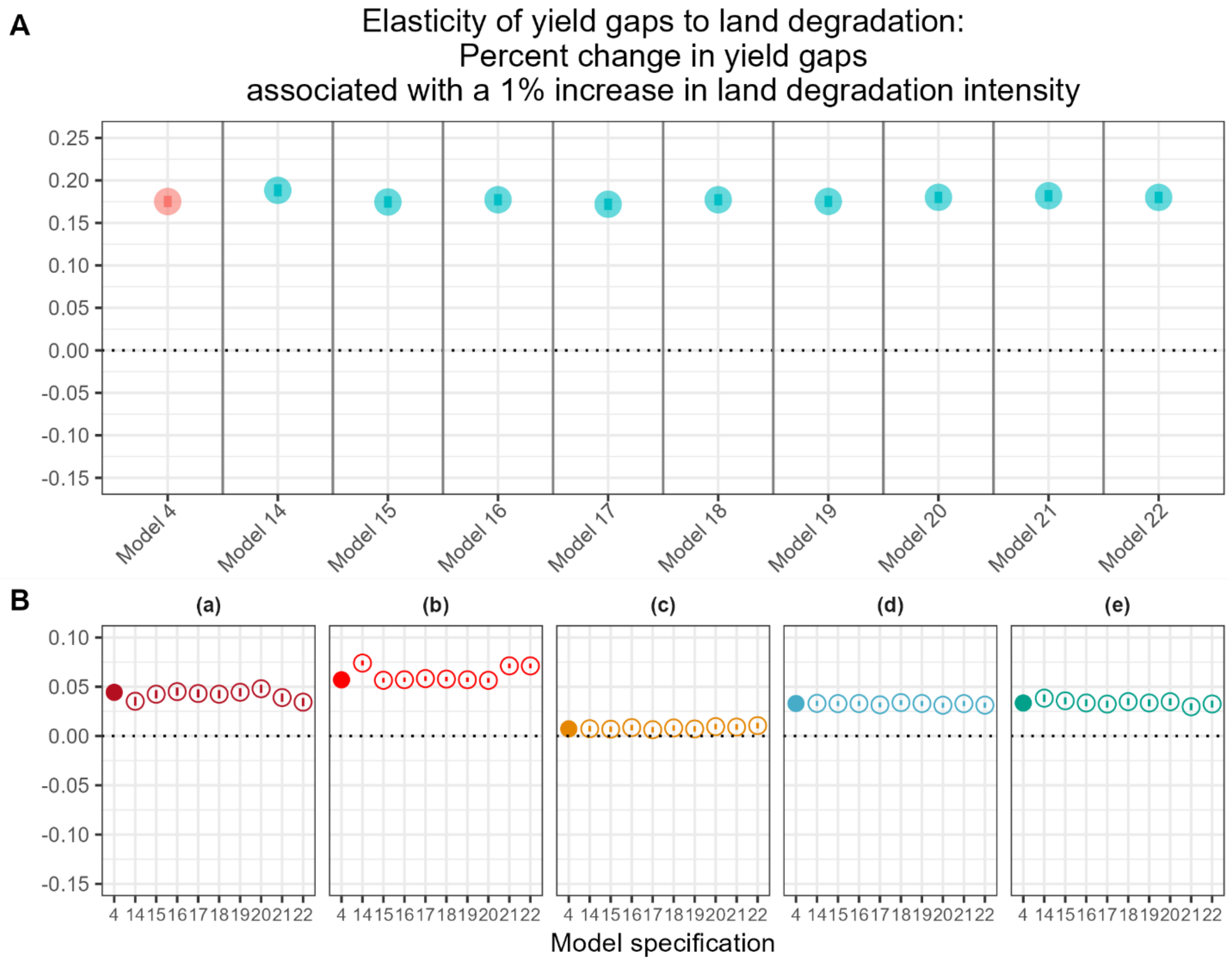

Supplementary Fig. 3 Estimated association between land degradation and crop yield gaps across different additional regression model specifications for robustness checks. Panel **A** shows the overall association for all land degradation variables combined. Panel **B** shows the association for each land degradation variable, i.e., soil erosion debt **(a)**, soil compaction debt **(b)**, soil organic carbon debt **(c)**, soil water debt **(d)**, and tree cover debt **(e)**. Points indicate the marginal association as elasticities (see the Methods section), i.e., the estimated mean percent (%) change in yield gaps associated with a 1% change in the land degradation measure (n = 405,084 grid cells). Error bars show the 95% confidence interval; the center of each error bar is the point estimate (mean elasticity from the regression). Exact two-sided p-values are provided in the Source Data file (Supplementary Data 1). Model 4 is the baseline preferred specification. The models tested below are based on Model 4. **Model 14** adds field size[102] as control. **Model 15** adds soil depth[100] as control. **Model 16** adds soil total nitrogen from SoilGrids 2.0[71] as control. **Model 17** adds groundwater table depth[101]. **Model 18** adds road density[106] as control. **Model 19** adds travel time to cities[107]. **Model 20** removes agricultural machinery[85] from controls set. **Model 21** adds agriculture share of government expenditure and agriculture orientation index for government expenditure (ratio of expenditure to revenue) from FAO[103] as controls. **Model 22** adds Economic Freedom Index by the Fraser Institute[104] as control. The spatial resolution of the analysis is 10 km.

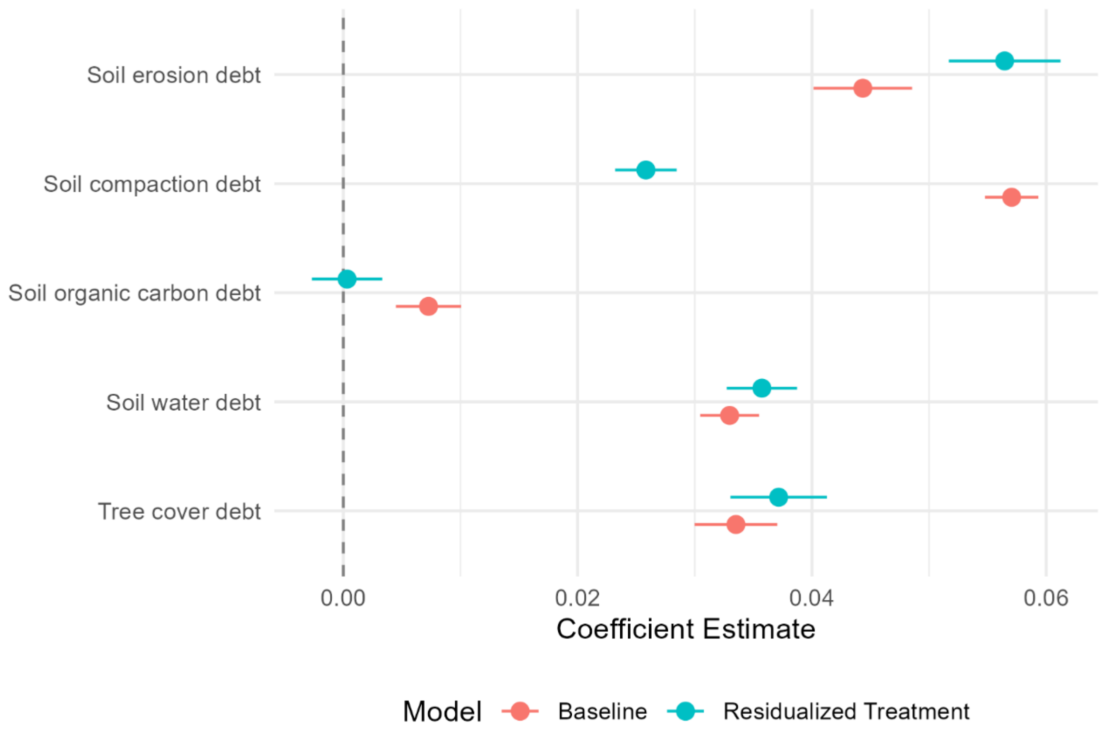

Supplementary Fig. 4 Robustness check comparing baseline model coefficients with residualized land degradation specification. Coefficients show the estimated association (elasticity) between land degradation indicators and yield gaps in the baseline model (which includes climate, soil, and management controls) compared to a specification using land degradation variables residualized with respect to climate, soil, and management controls. Error bars represent 95% confidence intervals. Points indicate the marginal association as elasticities (see the Methods section), i.e., the estimated mean percent (%) change in yield gaps associated with a 1% change in the land degradation measure (n = 405,084 grid cells). Error bars show the 95% confidence interval; the center of each error bar is the point estimate (mean elasticity from the regression). Exact two-sided p-values are provided in the Source Data file (Supplementary Data 1).

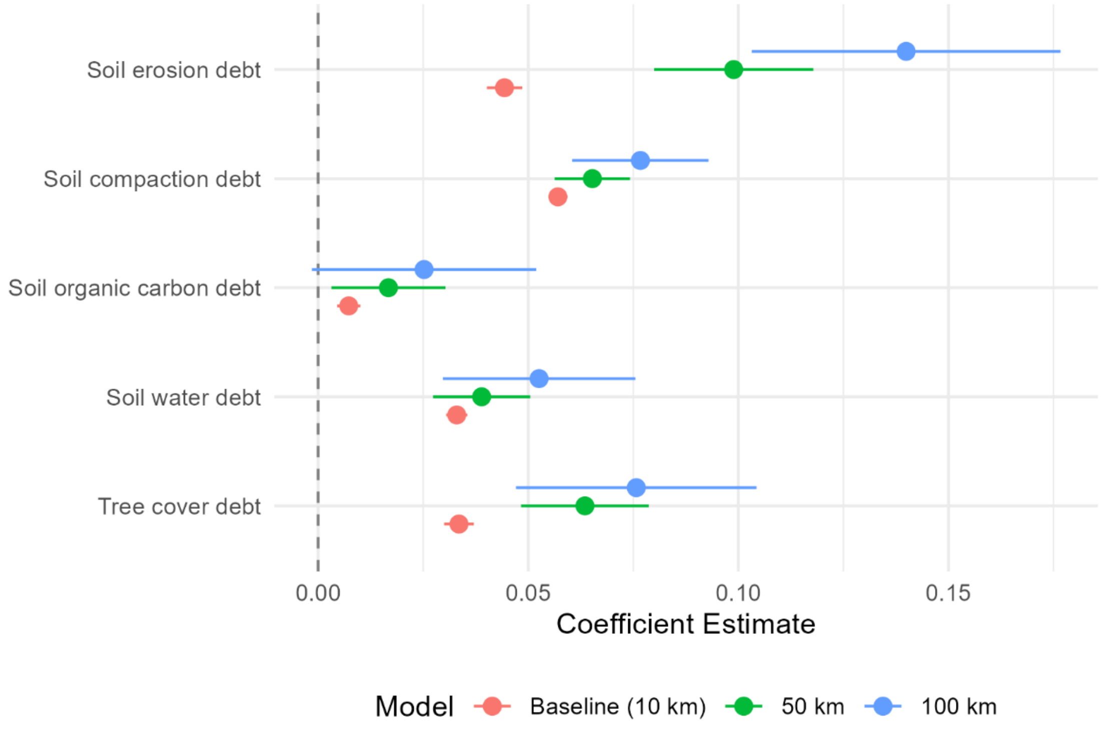

Supplementary Fig. 5 Robustness check comparing baseline model coefficients across spatial resolutions. Points show coefficient estimates with 95% confidence intervals for the baseline 10 km resolution (n = 405,084) compared to coarser resolutions of 50 km (n = 26,637) and 100 km (n = 8,450). Points indicate the marginal association as elasticities (see the Methods section), i.e., the estimated mean percent (%) change in yield gaps associated with a 1% change in the land degradation measure. Error bars show the 95% confidence interval; the center of each error bar is the point estimate (mean elasticity from the regression). Exact two-sided p-values are provided in the Source Data file (Supplementary Data 1).

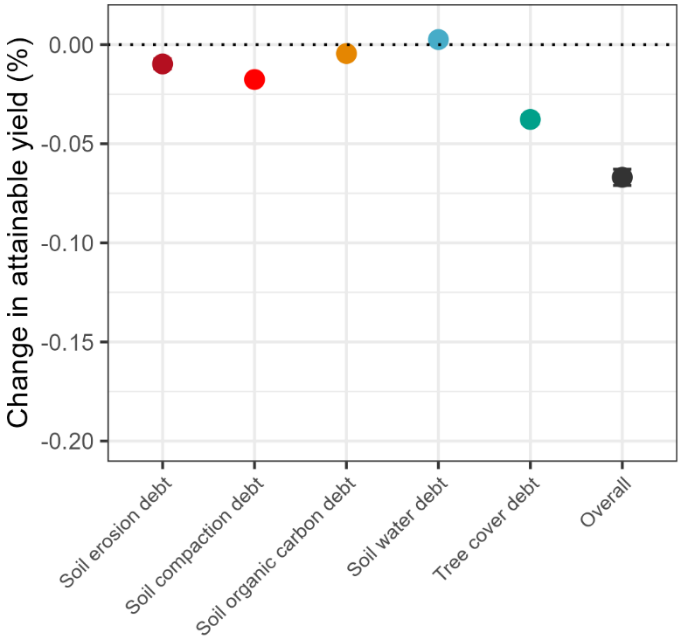

Supplementary Fig. 6 Land degradation and attainable yields. Points indicate the marginal association as elasticities (see the Methods section) i.e., the estimated percentage (%) change in attainable yield associated with a 1 percent increase in land degradation. Error bars show the 95% confidence interval. Based on **Model 4** (our preferred baseline specification, see **Fig. 2** in Main text). Points indicate the marginal association as elasticities (see the Methods section), i.e., the estimated mean percent (%) change in attainable yield associated with a 1% change in the land degradation measure (n = 405,084 grid cells). Error bars show the 95% confidence interval; the center of each error bar is the point estimate (mean elasticity from the regression). Exact two-sided p-values are provided in the Source Data file (Supplementary Data 1).

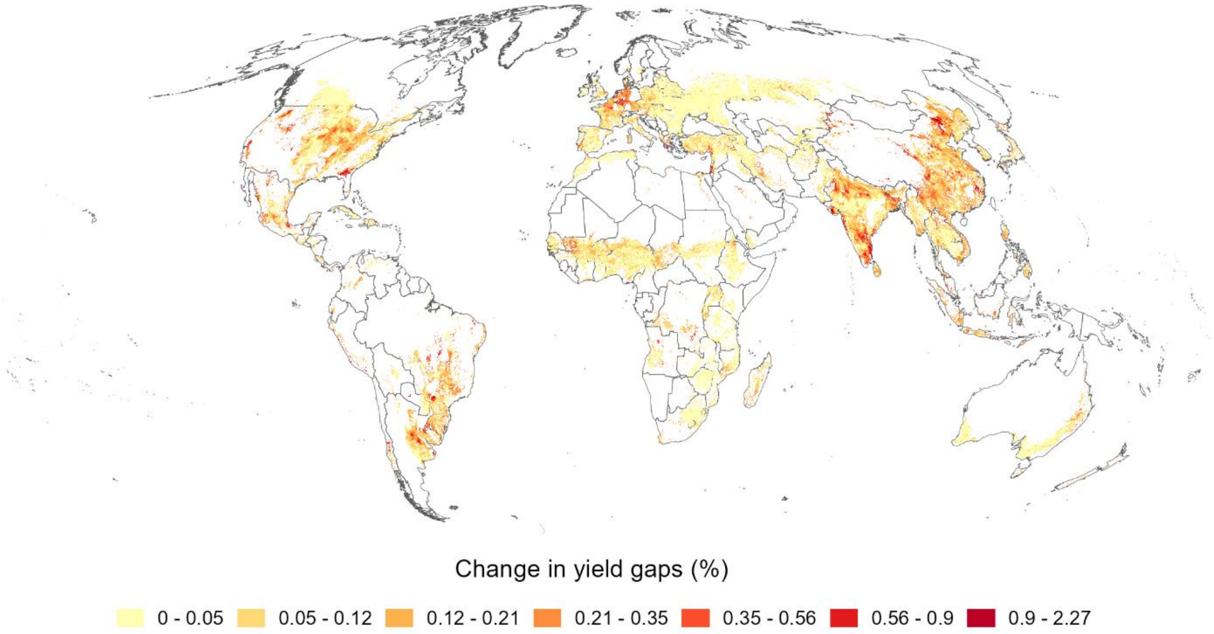

Supplementary Fig. 7 Estimated increase in crop yield gaps associated with overall land degradation. Values show marginal increase as elasticities i.e., the percent (%) increase in yield gaps associated with a 1% increase in land degradation magnitude. The visualization uses seven classes determined by the Fisher-Jenks algorithm for natural breaks. The spatial resolution of the analysis is 10 km. The base map is from the mapdata package (TM World Borders Dataset 0.3) in R (https://search.r-project.org/CRAN/refmans/prevR/html/TMWorldBorders.html).

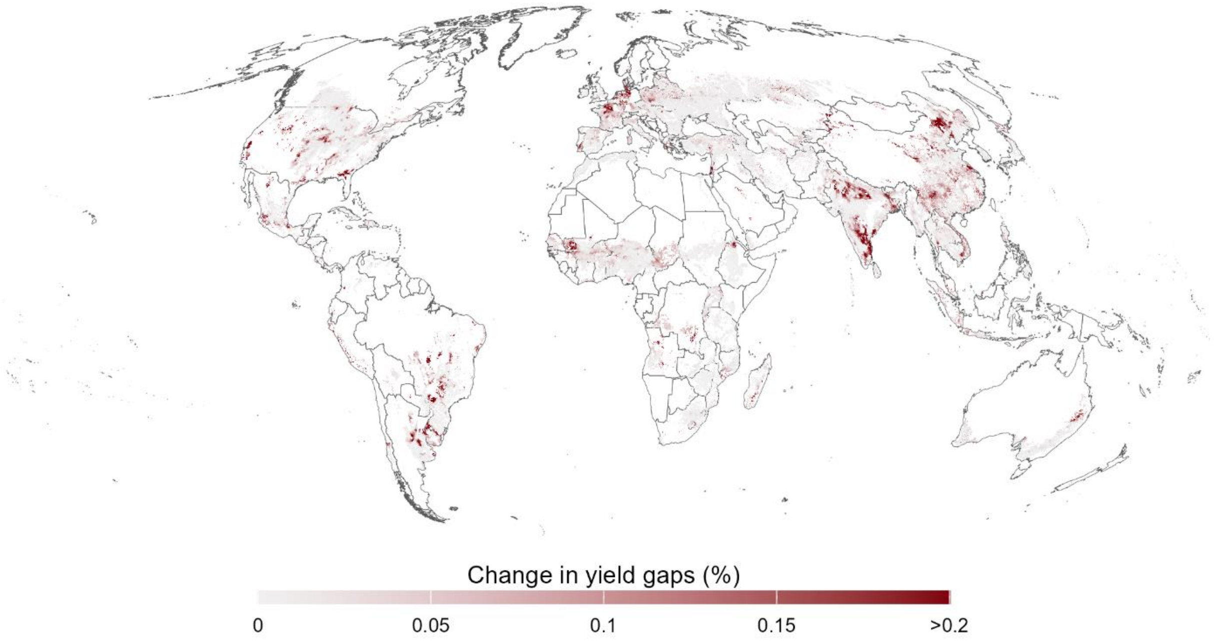

Supplementary Fig. 8 Estimated increase in crop yield gaps associated with soil erosion debt. Values show marginal increase as elasticities i.e., the percent (%) increase in yield gaps associated with a 1% increase in land degradation magnitude**.** The spatial resolution of the analysis is 10 km. The 97.5th percentile, 99th percentile, 99.9th percentile, and maximum values at grid cell level are 0.20, 0.30, 0.64, 1.80%. The base map is from the mapdata package (TM World Borders Dataset 0.3) in R (https://search.r-project.org/CRAN/refmans/prevR/html/TMWorldBorders.html).

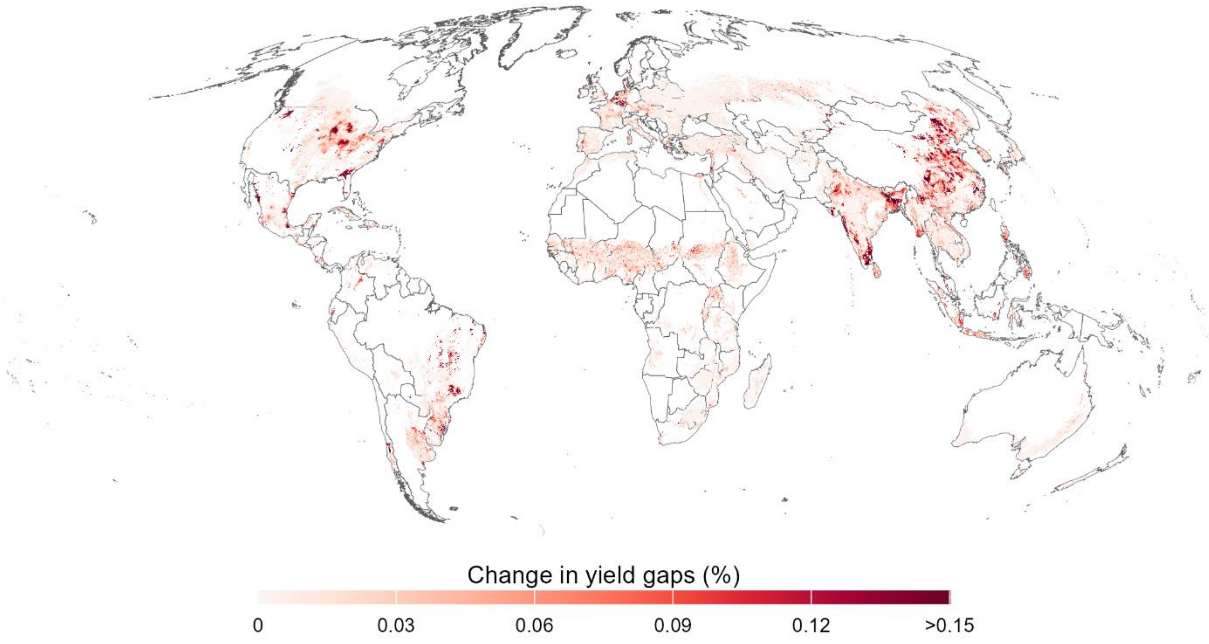

Supplementary Fig. 9 Estimated increase in crop yield gaps associated with soil compaction debt. Values show marginal increase as elasticities i.e., the percent (%) increase in yield gaps associated with a 1% increase in land degradation magnitude. The spatial resolution of the analysis is 10 km. The 97.5th percentile, 99th percentile, 99.9th percentile, and maximum values at grid cell level are 0.15, 0.24, 0.54, 1.37%. The base map is from the mapdata package (TM World Borders Dataset 0.3) in R (https://search.r-project.org/CRAN/refmans/prevR/html/TMWorldBorders.html).

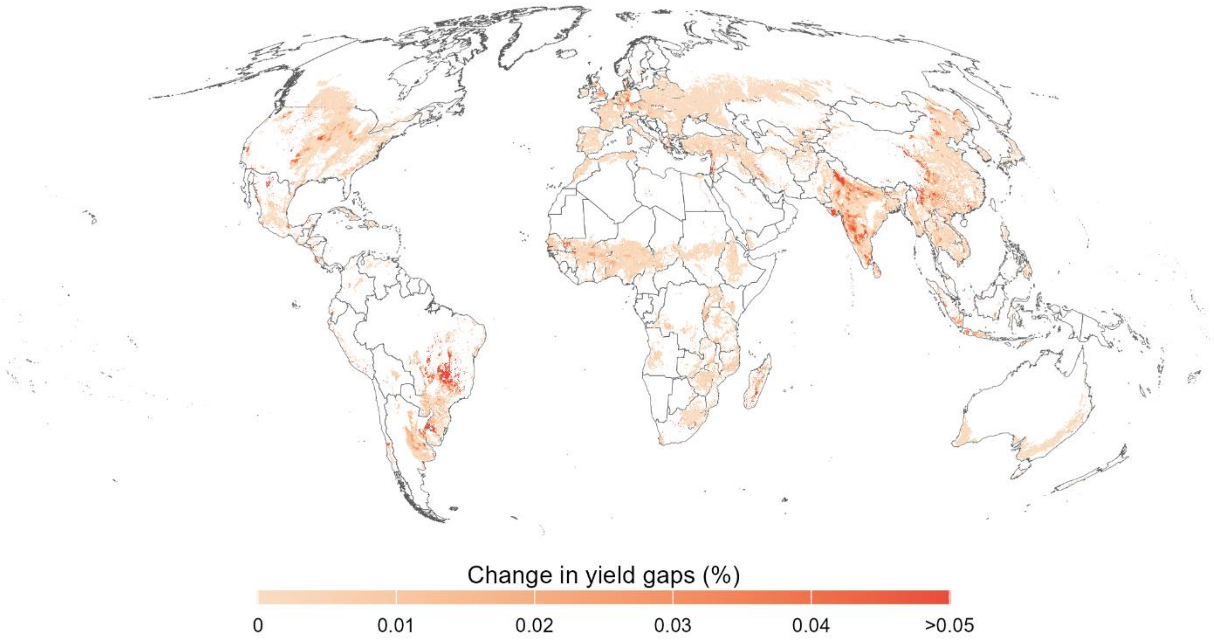

Supplementary Fig. 10 Estimated increase in crop yield gaps associated with soil organic carbon debt. Values show marginal increase as elasticities i.e., the percent (%) increase in yield gaps associated with a 1% increase in land degradation magnitude. The spatial resolution of the analysis is 10 km. The 97.5th percentile, 99th percentile, 99.9th percentile, and maximum values at grid cell level are 0.05, 0.08, 0.17, 0.38%. The base map is from the mapdata package (TM World Borders Dataset 0.3) in R (https://search.r-project.org/CRAN/refmans/prevR/html/TMWorldBorders.html).

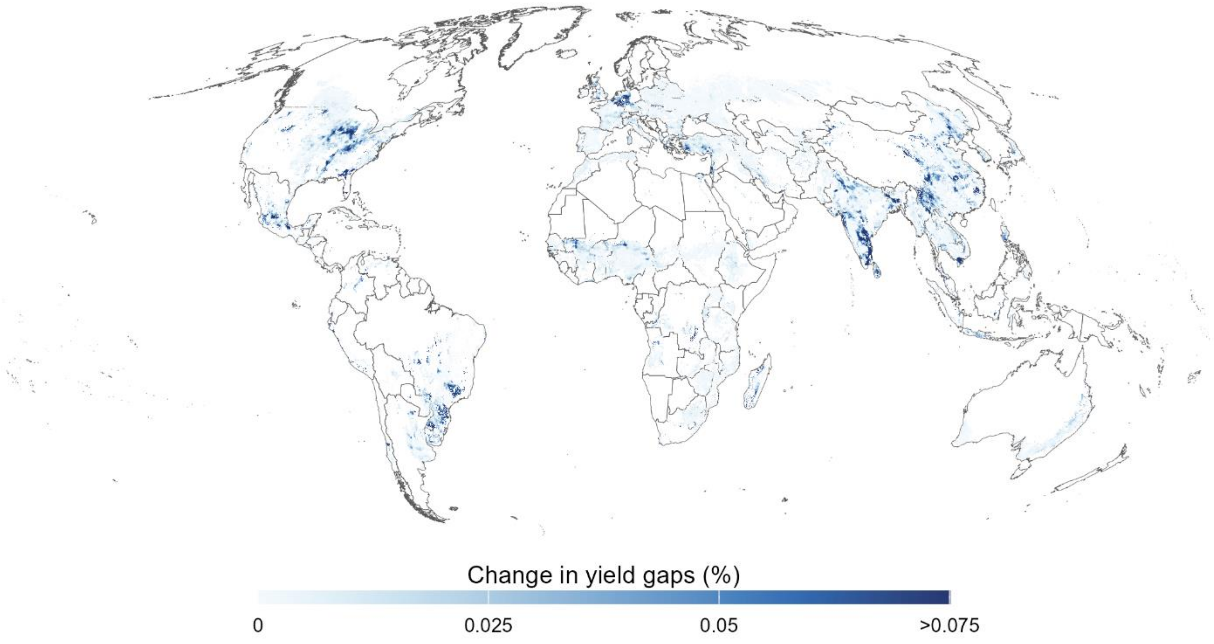

Supplementary Fig. 11 Estimated increase in crop yield gaps associated with soil water debt. Values show marginal increase as elasticities i.e., the percent (%) increase in yield gaps associated with a 1% increase in land degradation magnitude. The spatial resolution of the analysis is 10 km. The 97.5th percentile, 99th percentile, 99.9th percentile, and maximum values at grid cell level are 0.08, 0.12, 0.26, 0.60%. The base map is from the mapdata package (TM World Borders Dataset 0.3) in R (https://search.r-project.org/CRAN/refmans/prevR/html/TMWorldBorders.html).

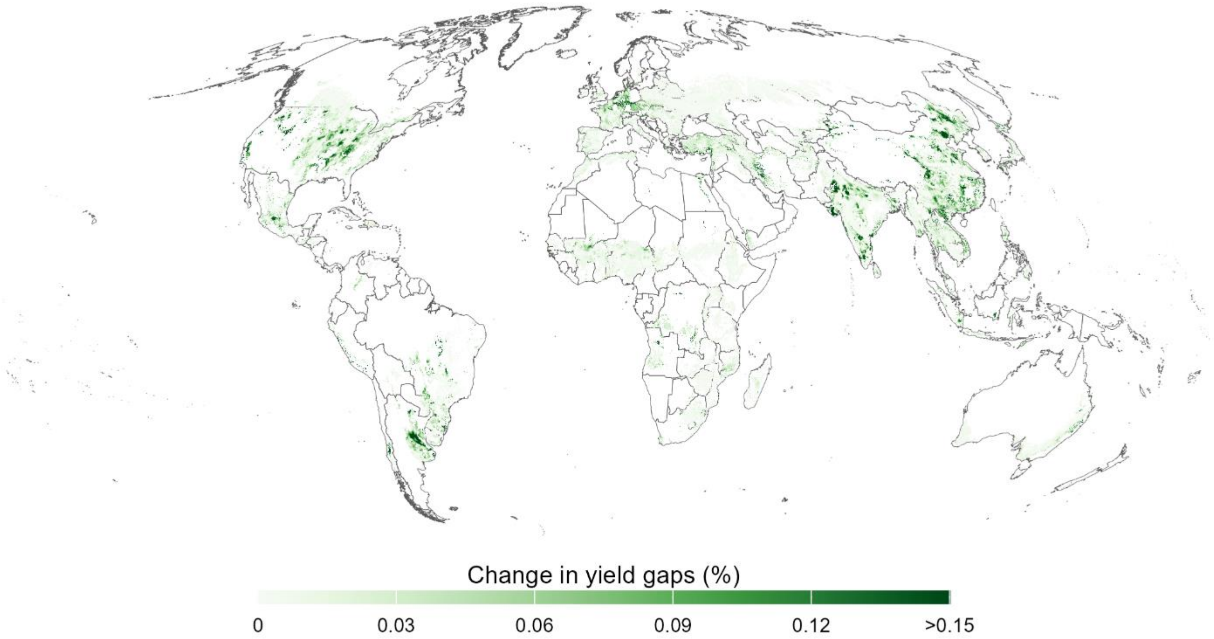

Supplementary Fig. 12 Estimated increase in crop yield gaps associated with tree cover debt. Values show marginal increase as elasticities i.e., the percent (%) increase in yield gaps associated with a 1% increase in land degradation magnitude. The spatial resolution of the analysis is 10 km. The 97.5th percentile, 99th percentile, 99.9th percentile, and maximum values at grid cell level are 0.14, 0.21, 0.41, 0.89%. The base map is from the mapdata package (TM World Borders Dataset 0.3) in R (https://search.r-project.org/CRAN/refmans/prevR/html/TMWorldBorders.html).

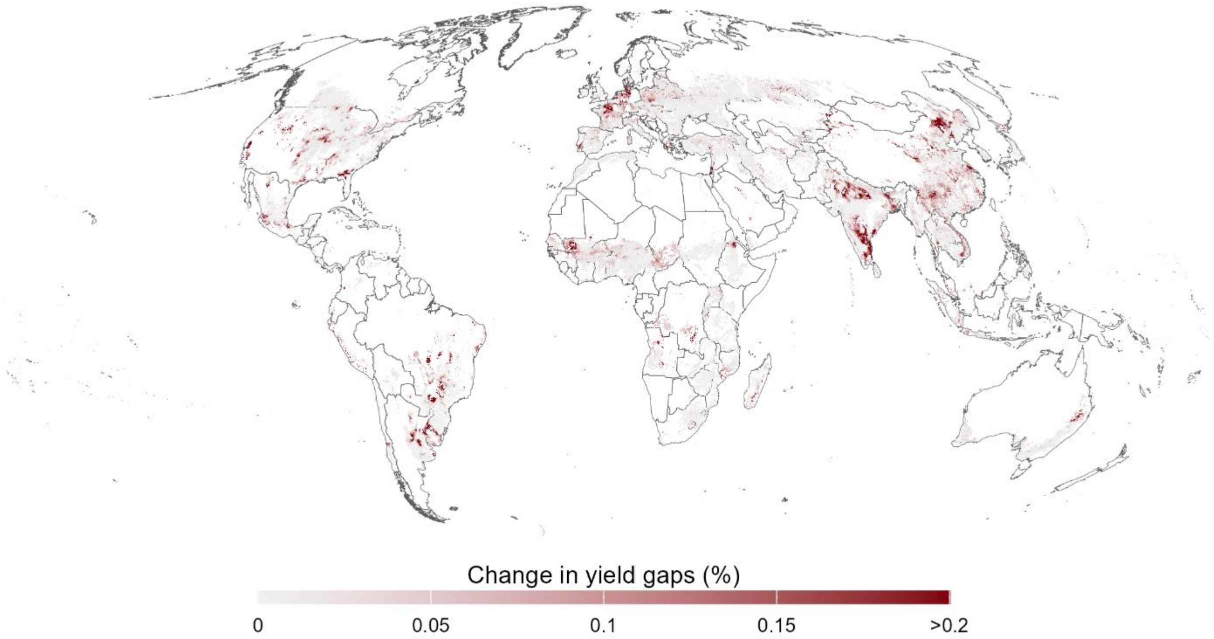

Supplementary Fig. 13 Estimated increase in crop yield gaps associated with soil erosion debt, with causal forest hyperparameters tuning (set to "all"). Values show marginal increase as elasticities i.e., the percent (%) increase in yield gaps associated with a 1% increase in land degradation magnitude. The spatial resolution of the analysis is 10 km. Computational time is 1.9x the default causal forest run. The base map is from the mapdata package (TM World Borders Dataset 0.3) in R (https://search.r-project.org/CRAN/refmans/prevR/html/TMWorldBorders.html).

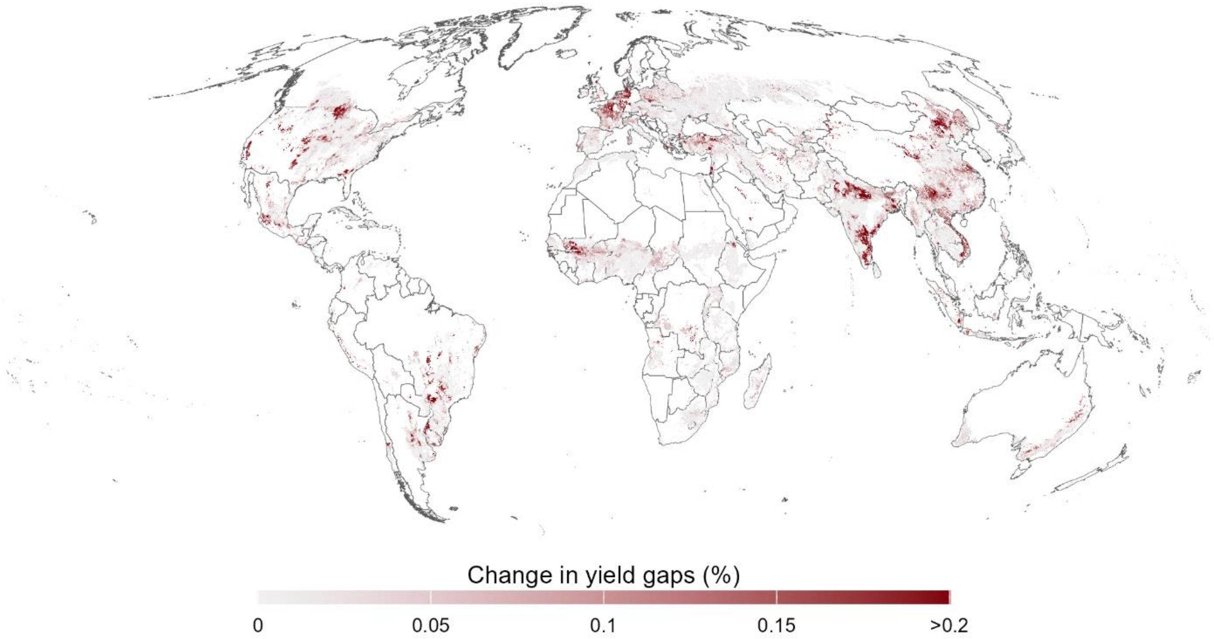

Supplementary Fig. 14 Estimated increase in crop yield gaps associated with soil erosion debt, with feature selection using multivariate adaptive regression splines (MARS) in both the treatment and outcome model subroutines of causal forest. Values show marginal increase as elasticities i.e., the percent (%) increase in yield gaps associated with a 1% increase in land degradation magnitude. The spatial resolution of the analysis is 10 km. Computational time is 3.3x the default causal forest run. The base map is from the mapdata package (TM World Borders Dataset 0.3) in R (https://search.r-project.org/CRAN/refmans/prevR/html/TMWorldBorders.html).

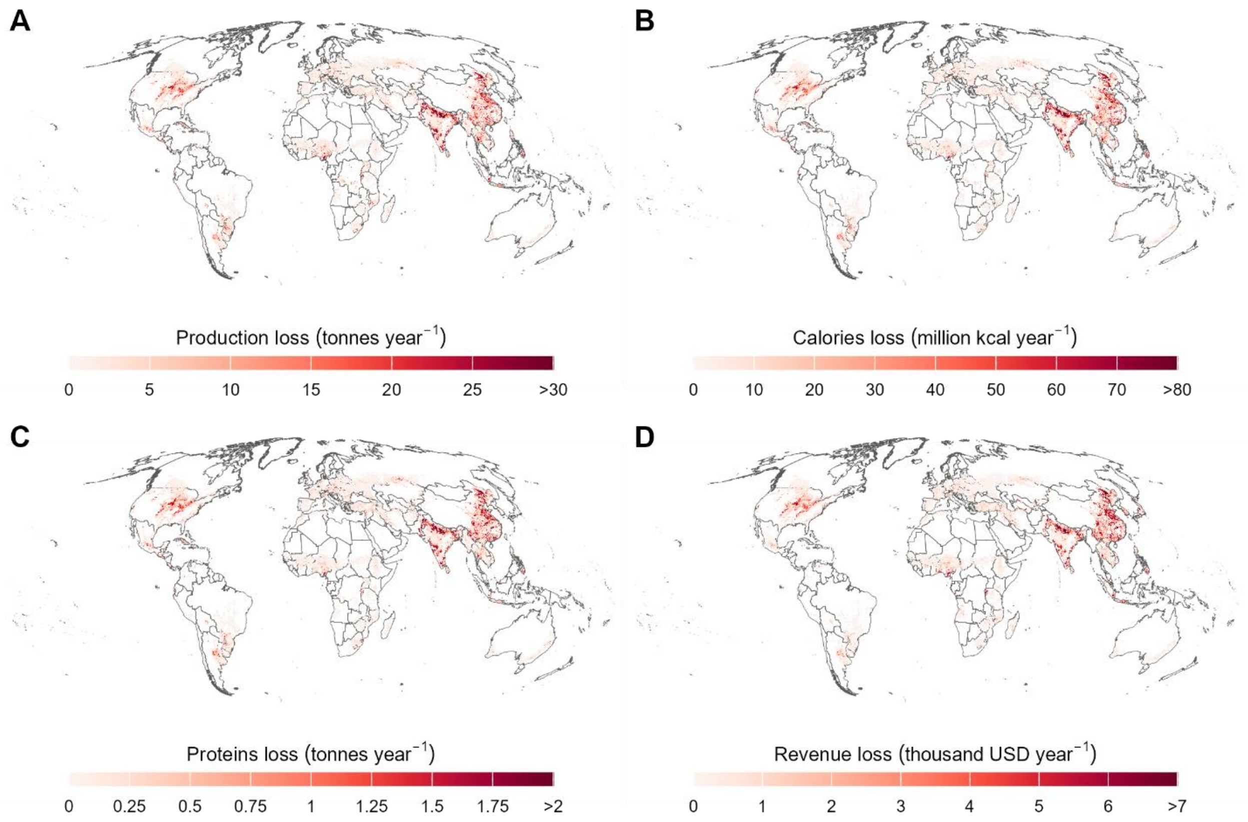

Supplementary Fig. 15 Estimates of production, calorie, protein, and revenue losses due to overall land degradation. Values show the marginal losses, per grid cell, associated with a 1% increase in land degradation magnitude. The spatial resolution of the analysis is 10 km. The 97.5th percentile, 99th percentile, 99.9th percentile, and maximum values at grid cell level for production loss is: 30, 49, 215, 3479 tonnes per year; calories loss: 78, 129, 551, 10392 million kcal per year; proteins loss: 2.0, 3.3, 13.4, 220 tonnes per year; revenues loss: 7279, 11702, 40793, 533626 USD per year. The base map is from the mapdata package (TM World Borders Dataset 0.3) in R (https://search.r-project.org/CRAN/refmans/prevR/html/TMWorldBorders.html).

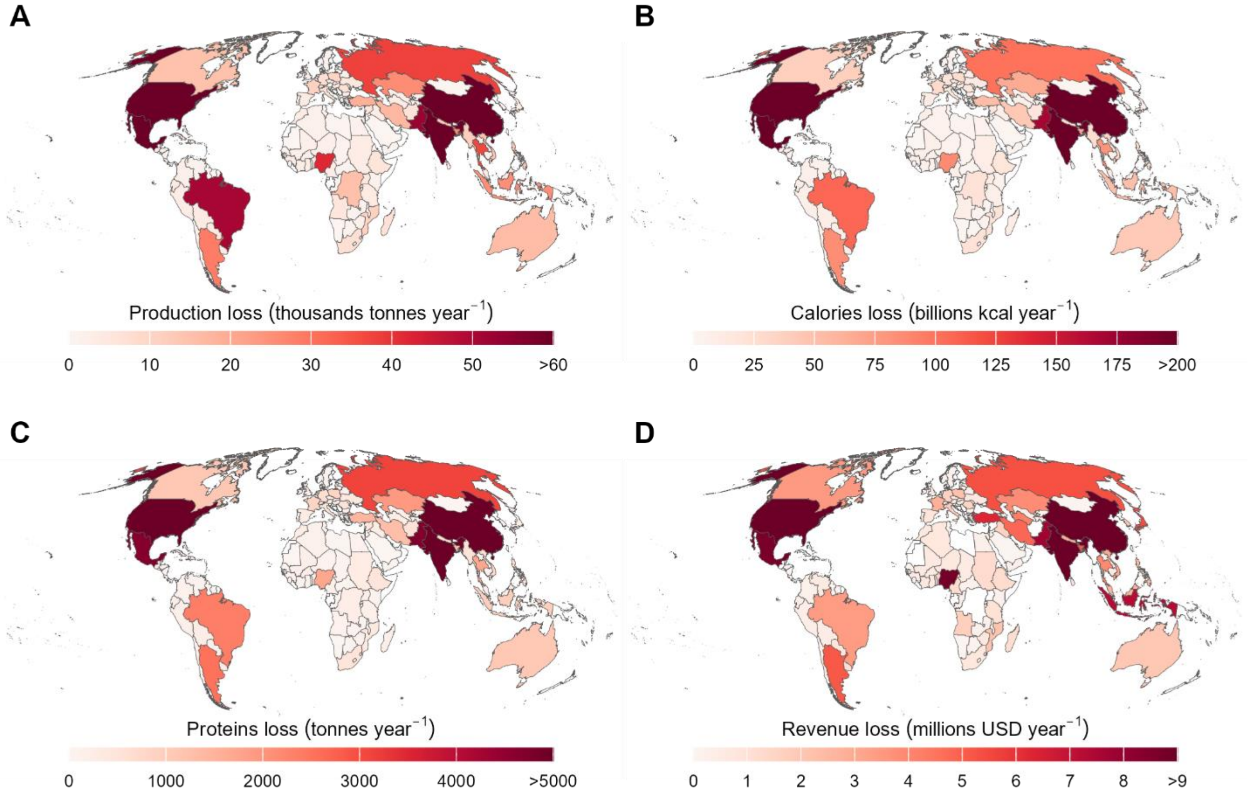

Supplementary Fig. 16 Estimates of country-aggregated (sum) loss of production, calories, proteins, and revenues associated with overall land degradation. Values show the marginal losses associated with a 1% increase in land degradation magnitude. The 97.5th percentile, 99th percentile, 99.9th percentile, and maximum values at country level for production loss are: 65, 398, 496, 497 thousand tonnes per year; calories loss: 200, 1028, 1337, 1350 billion kcal per year; proteins loss: 4682, 25843, 35886, 36770 tonnes per year; revenues loss: 8.8, 76.1, 138.7, 146.7 million USD per year. The base map is from the mapdata package (TM World Borders Dataset 0.3) in R (https://search.r-project.org/CRAN/refmans/prevR/html/TMWorldBorders.html).

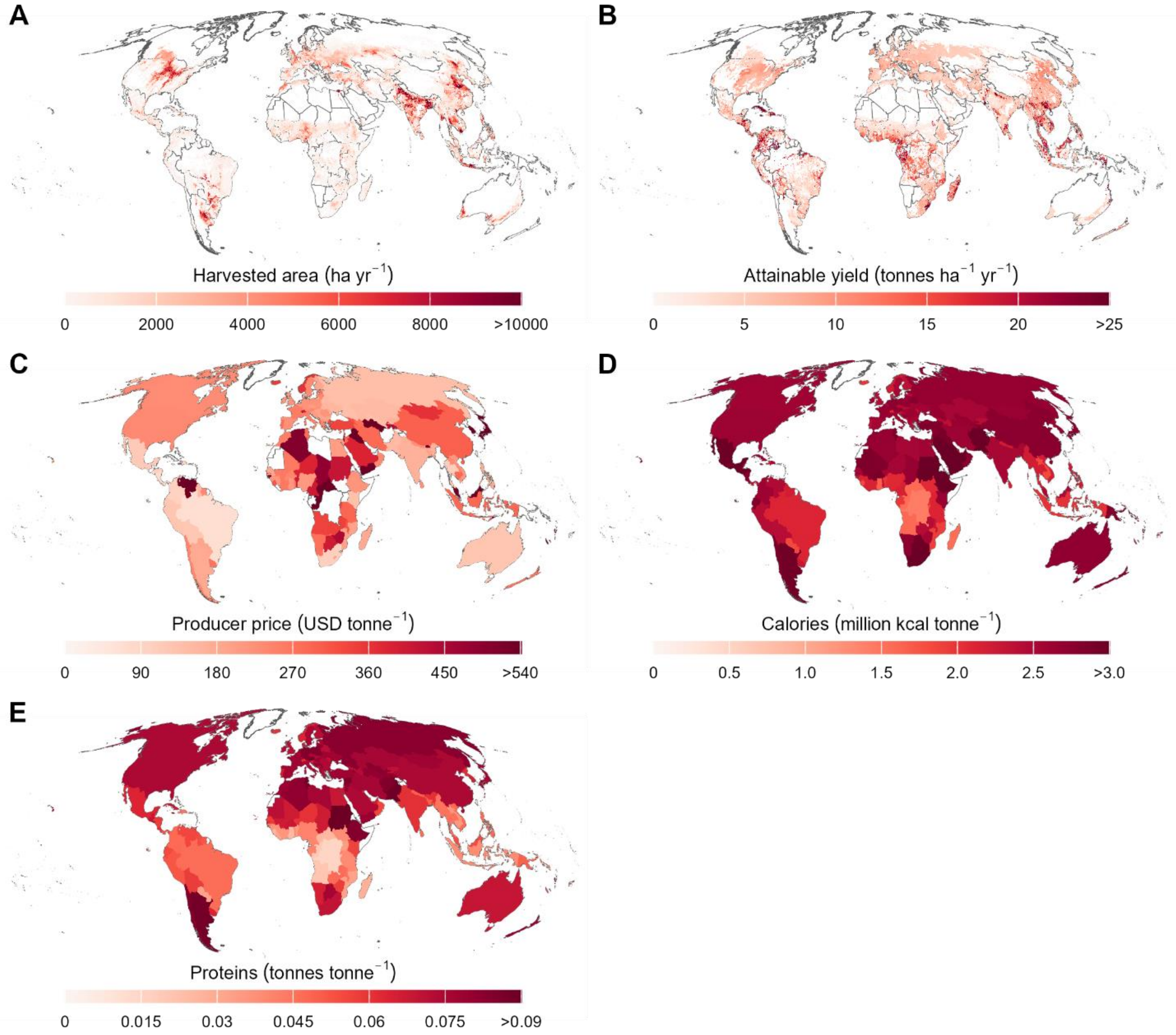

Supplementary Fig. 17 Input data for the calculations to translate the estimated change in yield gaps associated with land degradation, into corresponding change in food production, calories supply, proteins supply, and agricultural revenues. The data are all aggregated (weighted by harvested area) for the ten crops mapped in the yield gaps data, namely wheat, maize, rice, barley, sorghum, cassava, soybean, rapeseed, oil palm, and sugar cane. All data are for circa 2010. The 97.5th percentile, 99th percentile, 99.9th percentile, and maximum values are, for grid cell level (10 km) harvested area: 1582, 3641, 9458, 107040 ha per year; grid cell level (10 km) attainable yields: 26.0, 49.1, 100.1, 110.1 tonnes per ha per year; for country level producer price: 540, 727, 2055, 2156 USD per tonne; for country-level food calories supply: 3.18, 3.21, 3.30, 9.95 million kcal per tonne; and for country-level food proteins supply: 0.088, 0.090, 0.090, 0.306 tonnes per tonne. The base map is from the mapdata package (TM World Borders Dataset 0.3) in R (https://search.r-project.org/CRAN/refmans/prevR/html/TMWorldBorders.html), and from the Natural Earth dataset, accessed via the World dataset in the R tmap package (https://www.naturalearthdata.com/).

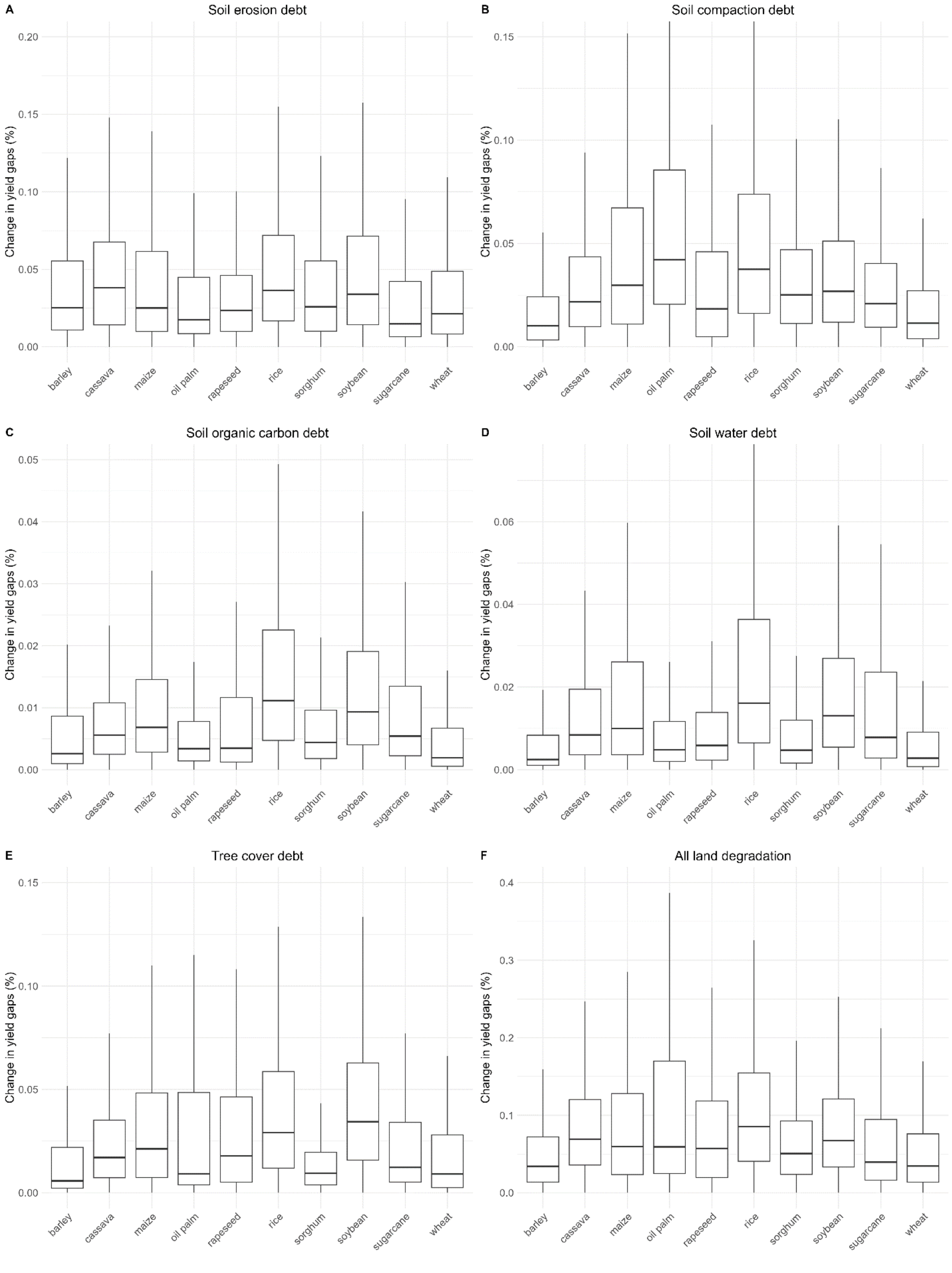

Supplementary Fig. 18 Yield gap elasticity to land degradation by dominant crop type. Values show the percent increase in yield gaps per 1% increase in land degradation magnitude, stratified by the dominant crop type.

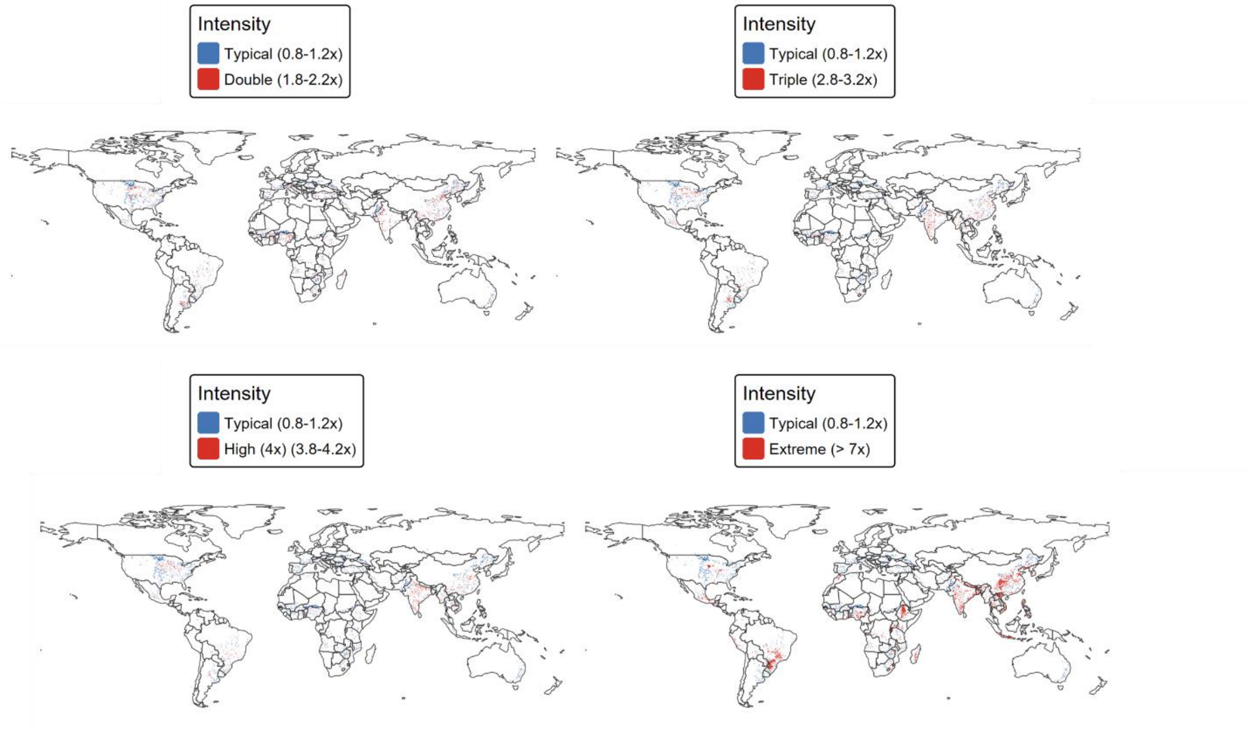

Supplementary Fig. 19 Spatial distribution of soil erosion debt at multiple intensity levels relative to the global median. Values are expressed as multiples of the global median soil erosion debt. For example, a value of 2× indicates soil erosion debt is twice the global median. The base map is from the Natural Earth dataset, accessed via the World dataset in the R tmap package (https://www.naturalearthdata.com/).

Supplementary Tables

Supplementary Table 1 Summary of coefficients (elasticities) estimating the global association of overall land degradation to yield gaps, across different regression model specifications for robustness checks. Values in parentheses indicate one standard error. This table corresponds to the coefficient plots presented in **Fig. 2A** and **Supplementary Fig. 3**. The coefficient represents the marginal elasticity (see **Methods**) i.e., the percent (%) change in yield gaps associated with a 1% change in land degradation. For the main baseline specification Model 4, the list of environmental controls, management (agricultural input) controls, and socioeconomic controls can be found in **Supplementary Table 4**. *P < 0.05, **P < 0.01, ***P < 0.001. Exact two-sided p-values are provided in the Source Data file (Supplementary Data 1).

Model	Description	Coefficient	N	Adj R^2
1	No controls	-0.117 (0.003)***	409,487	0.100
2	Adds natural environmental controls	-0.045 (0.003)***	409,469	0.214
3	Adds natural environmental controls, and management (agricultural input) controls	0.100 (0.003)***	406,899	0.278
**4**	**Adds natural environmental controls, management controls, and socioeconomic controls**	**0.175 (0.003)*****	405,084	0.331
5	Model 4, adds longitude and latitude spatial trends as controls	0.149 (0.003)***	405,084	0.345
6	Model 4, adds crop type dummies as controls	0.174 (0.003)***	405,084	0.357
7	Model 4, omits predicted soil texture variables from controls	0.157 (0.003)***	405,084	0.319
8	Model 4, winsorize data at 2.5^{th} and 97.5^{th} quantiles	0.118 (0.003)***	405,084	0.305
9	Model 4, adds quadratic terms of land degradation variables	0.144 (0.005)***	405,084	0.334
10	Model 4, adds interaction terms between land degradation variables	0.167 (0.004)***	405,084	0.344
11	Model 10, adds interaction terms between land degradation variables and management variables	0.121 (0.004)***	405,084	0.366
12	Model 4, with heteroscedasticity-robust standard errors	0.175 (0.004)***	405,084	0.331
13	Model 4, with Conley standard errors (100 km spatial bandwidth)	0.175 (0.032)***	405,084	0.331
14	Model 4, adds predicted field size category as control	0.188 (0.004)***	368,423	0.348
15	Model 4, adds predicted soil depth as control	0.175 (0.003)***	405,083	0.331
16	Model 4, adds predicted soil total nitrogen as control	0.177 (0.003)***	405,084	0.331

17	Model 4, adds predicted groundwater table depth (without human use) as control	0.172 (0.003)***	405,084	0.332
18	Model 4, adds road density as control	0.177 (0.003)***	405,079	0.332
19	Model 4, adds travel time to cities as control	0.175 (0.003)***	405,084	0.331
20	Model 4, omits agricultural machinery from control set	0.180 (0.003)***	405,084	0.331
21	Model 4, adds agriculture share of government expenditure and agriculture orientation index for government expenditure as controls	0.182 (0.003)***	394,475	0.335
22	Model 4, adds Economic Freedom Index as control	0.180 (0.003)***	398,971	0.338

Supplementary Table 2 Interpretation of a 10% increase in land degradation indicators. Values are reported in the original units of each land-degradation indicator. Spatial SD values are implied by the reported absolute 10% changes and the reported fractions of one spatial SD.

Land degradation indicators	Mean	Median	75th percentile	90th percentile	Absolute change corresponding to a 10% increase at the mean	Spatial SD	10% increase as fraction of SD
Soil erosion debt (Mg ha^{-1} yr^{-1})	4.54	1.61	5.41	11.07	0.45	9.00	0.05
Soil compaction debt (index points)	0.40	0.17	0.68	1.26	0.04	0.50	0.08
Soil organic carbon debt (Mg ha^{-1})	0.60	0.00	0.30	2.12	0.06	1.50	0.04
Soil water debt (m^3 m^{-3})	0.010	0.000	0.010	0.020	0.001	0.014	0.07
Tree cover debt (ha)	1,290.00	1,114.89	1,721.77	2,421.34	129.00	921.43	0.14

Supplementary Table 3 Country-level absolute losses associated with a 1% increase in land degradation for the 10 countries with the highest production losses. Countries are ranked by production loss. Revenue, calorie, and protein rankings may differ. Persons per year are calculated as calorie losses divided by 2000 kcal $person^{-1}$ day^{-1}.

Rank	ISO3	Country	Production loss (‘000 tonnes yr^{-1})	Calories loss (billion kcal yr^{-1})	Persons yr^{-1} at 2000 kcal $person^{-1}$ day^{-1}	Protein loss (tonnes yr^{-1})	Revenue loss (million USD yr^{-1})
1	IND	India	496.9	1,253.5	1,717,169	30,268.1	82.2
2	CHN	China	489.9	1,349.8	1,849,001	36,770.4	146.7
3	USA	United States	240.4	627.5	859,637	17,976.1	56.7
4	MEX	Mexico	73.9	220.8	302,427	4,673.9	8.7
5	PAK	Pakistan	52.6	168.8	231,213	4,687.2	7.7
6	BRA	Brazil	50.5	106.9	146,386	2,363.4	3.4
7	NGA	Nigeria	41.7	86.6	118,683	1,722.6	8.8
8	RUS	Russia	37.9	101.1	138,466	3,135.6	5.3
9	THA	Thailand	37.0	81.3	111,302	1,702.6	4.0
10	ARG	Argentina	28.6	84.8	116,223	2,524.8	5.2

Supplementary Table 4 Summary of data as used in the main estimation sample i.e., after resampling to 10-km common resolution, and filtering for grid cells with values for all variables available. N = 405,084.

Variable	Unit/definition	Original spatial resolution (resampling)	Measurement frequency and temporal aggregation	Mean (standard deviation; minimum-maximum)	Source
Outcome					
**Yield gaps (aggregated for ten crops)**	% relative to attainable yields, yield gaps is attainable (potential) yields minus attained (actual) yields	Spatialized into 10-km, based on ~20000 political units	2010	44.72 (21.52; 0-99.55)	Ref. 21
Land degradation variables					
**Soil erosion debt**	Mg ha^{-1} yr^{-1}	1-km (mean)	Debt is calculated as modelled current (actual) soil erosion rate for 2012, minus modelled native (potential) soil erosion rate based on potential no-human percent tree cover[29].	4.54 (8.3; 0-162.85)	Ref. 1
**Soil compaction debt**	Unitless, ratio of soil stress due to agricultural machinery, to soil strength	11-km (nearest neighbor)	The subsoil compaction susceptibility index is interpreted as debt. It is modelled based on tractor density data with most recent record is for 2009.	0.41 (0.49; 0-2)	Ref. 35
**Soil organic carbon (SOC) debt**	tonnes C ha^{-1}	5-km (mean)	From the annual SOC maps starting from 1981, we take average of 1981-1985 for ca. 1980, and average of 2008-2012 for ca. 2010. Debt is calculated as ca. 1980 value minus ca. 2010 value i.e., as long-term decline in SOC.	0.58 (1.4; 0-19.72)	Ref. 36
**Soil water debt**	m^3 m^{-3}	25-km (nearest neighbor)	From the daily soil moisture data records starting	0.01 (0.01; 0-0.23)	Based on ESA CCI soil moisture products[37–39]

			from 1979, we take average of 1979-1983 for ca. 1980, and average of 2008-2012 for ca. 2010. Debt is ca. 1980 value minus ca. 2010 value i.e., as long-term decline in soil water.		
**Tree cover debt**	hectares	1-km (sum)	Debt is calculated as potential no-human percent tree cover[29] minus current tree cover[73].	1289.62 (847.73; 0-6789.46)	Ref. 1
Controls (natural environmental)					
Climatological solar radiation	kJ m^{-2} day^{-1}	1-km (mean)	Average over 1970-2000 period, from the monthly data, take annual mean	15496.76 (3099.45; 8157.72-22996.33)	WorldClim 2.1[74]
Climatological average temperature	°C	1-km (mean)	Average over 1970-2000 period, from the monthly data, take annual mean	14.9 (8.42; -4.14-30.52)	WorldClim 2.1[74]
Climatological total precipitation	mm	1-km (mean)	Average over 1970-2000 period, from the monthly data, take annual sum	823.37 (495.4; 0-6498.83)	WorldClim 2.1[74]
Climatological average temperature of warmest quarter	°C	1-km (mean)	Average over 1970-2000 period.	23.27 (5.01; 5.92-37.38)	WorldClim 2.1[74]
Climatological maximum temperature of warmest month	°C	1-km (mean)	Average over 1970-2000 period.	30.5 (5.39; 12.22-47.01)	WorldClim 2.1[74]
Climatological total precipitation of wettest quarter	mm	1-km (mean)	Average over 1970-2000 period.	394.36 (282.32; 0-5538.27)	WorldClim 2.1[74]
Climatological aridity	Unitless, ratio between precipitation and potential	1-km (mean)	Average over 1970-2000 period.	5954.98 (3582.03; 3-39553.08)	WorldClim 2.1[74]

	evapotranspiration (scaled by 10000)				
Current total precipitation	mm	4.6-km (mean)	From monthly data, take average of 2008-2012 for ca. 2010.	842.71 (526.41; 0-7091.1)	TerraClimate[75]
Current mean of minimum temperature	°C (scaling factor 0.1)	4.6-km (mean)	From monthly data, take average of 2008-2012 for ca. 2010.	96.85 (81.73; -110.46-263.7)	TerraClimate[75]
Current mean of maximum temperature	°C (scaling factor 0.1)	4.6-km (mean)	From monthly data, take average of 2008-2012 for ca. 2010.	212.67 (88.12; 21.79-390.9)	TerraClimate[75]
Current mean solar radiation	W m^{-2} (scaling factor 0.1)	4.6-km (mean)	From monthly data, take average of 2008-2012 for ca. 2010.	1824.27 (352.57; 931.98-2745.98)	TerraClimate[75]
Change in precipitation between ca. 1980 and ca. 2010	mm	4.6-km (mean)	From monthly data, take average of 2008-2012 for ca. 2010, and average of 1978-1982 for ca. 1980. Change is ca. 2010 value minus ca. 1980 value.	16.48 (124.64; -841-1504.15)	TerraClimate[75]
Change in minimum temperature between ca. 1980 and ca. 2010	°C (scaling factor 0.1)	4.6-km (mean)	From monthly data, take average of 2008-2012 for ca. 2010, and average of 1978-1982 for ca. 1980. Change is ca. 2010 value minus ca. 1980 value.	6.44 (4.65; -11.43-28.22)	TerraClimate[75]
Change in maximum temperature between ca. 1980 and ca. 2010	°C (scaling factor 0.1)	4.6-km (mean)	From monthly data, take average of 2008-2012 for ca. 2010, and average of 1978-1982 for ca. 1980. Change is ca. 2010 value minus ca. 1980 value.	7.12 (5.28; -13.63-20.71)	TerraClimate[75]
Change in solar radiation between ca. 1980 and ca. 2010	W $^{m-2}$ (scaling factor 0.1)	4.6-km (mean)	From monthly data, take average of 2008-2012 for ca. 2010, and average of 1978-1982 for ca. 1980. Change is ca. 2010 value minus ca. 1980 value.	29.53 (64.9; -520.6-300.42)	TerraClimate[75]

Elevation	Meters (above sea level)	231-m (mean)	One time data (static).	516.38 (553.92; -316.59-4752.27)	GMTED[76]
Slope	degrees	231-m (mean)	Derived from elevation. One time data.	3.55 (4.6; 0-39)	GMTED[76]
Terrain ruggedness index	Index	90-m (mean)	One time data (static).	5.21 (7.18; 0.07-63.24)	Geomorpho90m[77]
Soil type	Categorical (1-13)	231-m (mode)	One time data (static). 13 USDA soil great groups.	Mollisols (32.1%); Alfisols (22.6%); Inceptisols (13.9%); Entisols (8.5%); Ultisols (7.4%); Vertisols (6.1%); Oxisols (4.6%); Aridisols (3.1%); Spodosols (1.5%); Histosols (0.1%)	Ref. 78
Soil sand content	g kg^{-1}	250-m (mean)	One time data (static).	375.3 (151.11; 9.7-957.4)	Soil Grids v2.0[71]
Soil silt content	g kg^{-1}	250-m (mean)	One time data (static).	344.07 (115.01; 20.14-761.55)	Soil Grids v2.0[71]
Soil clay content	g kg^{-1}	250-m (mean)	One time data (static).	280.63 (77.38; 20.41-656.86)	Soil Grids v2.0[71]
Controls (adaptation, agricultural inputs, management)					
NH4 fertilizer application	g N per grid cell	10-km (nearest neighbor)	From annual data, take average of 2008-2012 for ca. 2010.	154083272.46 (231873400.74; 0-1933074560)	HaNi[79]
NO3 fertilizer application	g N per grid cell	10-km (nearest neighbor)	From annual data, take average of 2008-2012 for ca. 2010.	16693053.86 (21708835.28; 0-348030496)	HaNi[79]
Manure fertilizer application	g N per grid cell	10-km (nearest neighbor)	From annual data, take average of 2008-2012 for ca. 2010.	37994049.71 (51890521.37; 0-1004916608)	HaNi[79]
NOy deposition	g N per grid cell	10-km (nearest neighbor)	From annual data, take average of 2008-2012 for ca. 2010.	26078421.33 (19603105.36; 909722.44-139149504)	HaNi[79]

NHx deposition	g N per grid cell	10-km (nearest neighbor)	From annual data, take average of 2008-2012 for ca. 2010.	30214724.03 (23751142.74; 406334.88-626110272)	HaNi[79]
Soil phosphorus	mg kg^{-1}	1-km (mean)	One time data (static). Predicted using satellite climate and vegetation covariates for ~2010.	19.12 (23.37; 0-180)	Ref. 80
Pesticide application	Kg ha^{-1}	10-km (nearest neighbor)	One time data (static). Predicted for 2015. Take sum of all pesticide species, for all crops, the high estimates.	22.23 (25.09; 0-207.23)	Ref. 83
Area equipped for irrigation	hectares	10-km (nearest neighbor)	Every 10 or 5 years. Use value for 2010.	589.25 (1272.4; 0-8578.9)	Ref. 81,82
Farm machinery	Farm inventories of farm machinery, measured in thousands of metric horsepower (1000 CV) in tractors, combine-threshers, and milking machines	Country level (rasterize to yield gaps grid)	From annual data, take average of 2008-2012 for ca. 2010.	193135.22 (393290.97; 0.95-1258356.75)	USDA International Agricultural Productivity[85]
Employment in agriculture	% of total employment (share of working age persons who were engaged in the agricultural sector to total employment)	Country level (rasterize to yield gaps grid)	From annual data, take average of 2008-2012 for ca. 2010.	25.6 (22.35; 0.99-88.2)	World Bank[84,122]
Controls (socioeconomic, institution, policies, governance)					
Human development index	Index (0-1; higher value means higher levels of human development, based on health, education, and standard of living)	10-km (nearest neighbor)	From annual data, take average of 2008-2012 for ca. 2010.	0.71 (0.16; 0.32-0.99)	Ref. 88
Gross domestic product (GDP)	US dollar (total GDP = gridded GDP per capita multiplied by gridded population data)	10-km (nearest neighbor)	From annual data, take average of 2008-2012 for ca. 2010.	95059618.63 (381422718.01; 0-36162220032)	Ref. 88

GDP per capita	US dollar	10-km (nearest neighbor)	From annual data, take average of 2008-2012 for ca. 2010.	18014.29 (17918.27; 504.57-150341.81)	Ref. 88
Agriculture GDP	% of total GDP (contributions of the agricultural sector to GDP)	Country level (rasterize to yield gaps grid)	From annual data, take average of 2008-2012 for ca. 2010.	10.06 (10.28; 0.27-53.41)	World Bank[89,122]
Access to electricity in rural area	% of rural population	Country level (rasterize to yield gaps grid)	From annual data, take average of 2008-2012 for ca. 2010.	81.2 (31.66; 0.93-100)	World Bank[90,122]
Phone (mobile cellular) subscription	Number of persons per 100 people	Country level (rasterize to yield gaps grid)	From annual data, take average of 2008-2012 for ca. 2010.	87.08 (35.74; 2.52-173.68)	World Bank[91,122]
Property rights protection index	Index (0-100)	Country level (rasterize to yield gaps grid)	Collapsed across all years (mean) as in ref. 94.	52.18 (3.72; 44.9-60.41)	Ref. 94
Perceived environmental policy enforcement	Index (0-100). Developed by World Economic Forum, based on a survey of business leaders.	Country level (rasterize to yield gaps grid)	Collapsed across all years (mean) as in ref. 94.	36.51 (11.23; 14-62)	Ref. 92,94
Bayesian corruption index	Index (0-100). Bayesian Corruption Index of Standaert, based on corruption perceptions of inhabitants, companies, NGOs, governmental and supragovernmental organizations.	Country level (rasterize to yield gaps grid)	Collapsed across all years (mean) as in ref. 94.	46.34 (14.26; 7.63-74.96)	Ref. 93,94

Supplementary Table 5 Summary statistics of data as used in the main estimation sample i.e., after resampling to 10-km common resolution, and filtering for grid cells with values for all variables available. N = 405,084.

Variable	Mean	Median	SD	Min	Max	1th percentile	2.5th percentile	97.5th percentile	99th percentile
**Yield gaps**	44.72	45.11	21.52	0.00	99.55	0.00	2.77	82.52	89.41
**Soil erosion debt**	4.54	1.61	8.30	0.00	162.85	0.02	0.04	26.73	42.09
**Soil compaction debt**	0.41	0.17	0.49	0.00	2.00	0.00	0.00	1.55	1.63
**Soil organic carbon debt**	0.58	0.00	1.40	0.00	19.72	0.00	0.00	4.90	6.57
**Soil water debt**	0.01	0.00	0.01	0.00	0.23	0.00	0.00	0.03	0.04
**Tree cover debt**	1289.62	1114.89	847.73	0.00	6789.46	72.70	147.52	3411.00	4013.33
Change in precipitation between ca. 1980 and ca. 2010	16.48	6.55	124.64	-841.00	1504.15	-250.20	-185.15	264.25	421.21
Change in minimum temperature between ca. 1980 and ca. 2010	6.44	6.53	4.65	-11.43	28.22	-3.92	-2.66	15.13	16.28
Change in maximum temperature between ca. 1980 and ca. 2010	7.12	7.12	5.28	-13.63	20.71	-4.51	-2.98	16.23	17.45
Change in solar radiation	29.53	36.08	64.90	-520.60	300.42	-158.70	-109.29	164.75	205.82

Variable	Mean	Median	SD	Min	Max	1th percentile	2.5th percentile	97.5th percentile	99th percentile
between ca. 1980 and ca. 2010									
Current total precipitation	842.71	700.80	526.41	0.00	7091.10	121.04	208.45	2121.74	2777.51
Current mean of minimum temperature	96.85	91.38	81.73	-110.46	263.70	-50.37	-40.81	226.93	234.25
Current mean of maximum temperature	212.67	209.62	88.12	21.79	390.90	60.95	69.77	357.32	364.95
Current mean solar radiation	1824.27	1847.82	352.57	931.98	2745.98	1139.62	1201.78	2446.39	2525.33
Elevation	516.38	310.83	553.92	-316.59	4752.27	8.03	17.68	2076.38	2485.81
Slope	3.55	1.61	4.60	0.00	39.00	0.17	0.25	17.27	21.16
Terrain ruggedness index	5.21	2.20	7.18	0.07	63.24	0.14	0.18	26.66	32.86
Soil clay content	280.63	280.19	77.38	20.41	656.86	93.84	123.26	445.31	485.56
Soil sand content	375.30	357.01	151.11	9.70	957.40	70.09	106.72	711.58	756.25
Soil silt content	344.07	350.49	115.01	20.14	761.55	76.57	109.25	572.51	619.47
Climatological solar radiation	15496.76	15668.35	3099.45	8157.72	22996.33	9704.55	10096.57	20614.41	21072.89
Climatological average temperature	14.90	14.70	8.42	-4.14	30.52	0.45	1.29	28.01	28.54
Climatological total	823.37	675.20	495.40	0.00	6498.83	141.03	218.17	1990.88	2591.41

Variable	Mean	Median	SD	Min	Max	1th percentile	2.5th percentile	97.5th percentile	99th percentile
precipitation									
Climatological average temperature of warmest quarter	23.27	22.83	5.01	5.92	37.38	14.42	15.46	32.85	33.56
Climatological maximum temperature of warmest month	30.50	30.15	5.39	12.22	47.01	20.70	21.82	41.72	42.68
Climatological total precipitation of wettest quarter	394.36	313.38	282.32	0.00	5538.27	70.85	99.16	1035.76	1302.66
Climatological aridity	5954.98	5290.75	3582.03	3.00	39553.08	634.67	989.97	14312.74	17586.67
NH4 fertilizer application	154083272.46	68261472.00	231873400.74	0.00	1933074560.00	0.00	0.00	934556080.00	1125628604.16
NO3 fertilizer application	16693053.86	8568686.00	21708835.28	0.00	348030496.00	0.00	0.00	74199379.40	87463715.36
Manure fertilizer application	37994049.71	20765465.00	51890521.37	0.00	1004916608.00	0.00	0.00	189347922.00	260259667.84
NOy deposition	26078421.33	19917703.00	19603105.36	909722.44	139149504.00	4198763.03	5480583.26	80712767.00	95561821.04
NHx deposition	30214724.03	24654602.00	23751142.74	406334.88	626110272.00	3216339.20	4682007.65	83983641.40	97176033.84
Soil phosphorus	19.12	11.27	23.37	0.00	180.00	1.48	1.86	96.22	117.70

Variable	Mean	Median	SD	Min	Max	1th percentile	2.5th percentile	97.5th percentile	99th percentile
Pesticide application	22.23	12.36	25.09	0.00	207.23	0.00	0.01	78.27	85.48
Area equipped for irrigation	589.25	20.26	1272.40	0.00	8578.90	0.00	0.00	4806.89	6125.81
Human development index	0.71	0.74	0.16	0.32	0.99	0.37	0.39	0.94	0.95
Gross domestic product (GDP)	95059618.63	15146839.50	381422718.01	0.00	36162220032.00	0.00	164991.72	660823529.60	1320020194.56
GDP per capita	18014.29	11587.23	17918.27	504.57	150341.81	613.36	1076.27	55743.08	60113.04
Property rights protection index	52.18	51.26	3.72	44.90	60.41	46.54	46.98	59.12	59.15
Perceived environmental policy enforcement	36.51	36.00	11.23	14.00	62.00	14.00	14.00	57.00	61.00
Access to electricity in rural area	81.20	99.11	31.66	0.93	100.00	1.80	2.38	100.00	100.00
Phone (mobile cellular) subscription	87.08	92.09	35.74	2.52	173.68	6.17	20.49	150.35	150.35
Agriculture GDP	10.06	6.52	10.28	0.27	53.41	0.82	1.09	36.83	43.62
Employment in agriculture	25.60	20.65	22.35	0.99	88.20	0.99	1.37	74.01	76.31
Bayesian corruption index	46.34	47.23	14.26	7.63	74.96	16.50	16.50	66.01	70.70

Variable	Mean	Median	SD	Min	Max	1th percentile	2.5th percentile	97.5th percentile	99th percentile
Farm machinery	193135.22	15415.52	393290.97	0.95	1258356.75	9.20	10.50	1258356.75	1258356.75

References

1. Wuepper, D. *et al.* A ‘debt’ based approach to land degradation as an indicator of global change. *Global Change Biology* **27**, 5407–5410 (2021).
2. Asher, S., Lunt, T., Matsuura, R. & Novosad, P. Development Research at High Geographic Resolution: An Analysis of Night-Lights, Firms, and Poverty in India Using the SHRUG Open Data Platform. *The World Bank Economic Review* **35**, 845–871 (2021).
3. Liu, D., Mishra, A. K. & Ray, D. K. Sensitivity of global major crop yields to climate variables: A non-parametric elasticity analysis. *Science of The Total Environment* **748**, 141431 (2020).
4. Wei, T., Cherry, T. L., Glomrød, S. & Zhang, T. Climate change impacts on crop yield: Evidence from China. *Science of The Total Environment* **499**, 133–140 (2014).
5. Wollburg, P., Bentze, T., Lu, Y., Udry, C. & Gollin, D. Crop yields fail to rise in smallholder farming systems in sub-Saharan Africa. *Proc. Natl. Acad. Sci. U.S.A.* **121**, e2312519121 (2024).
6. Zhu, P. *et al.* Warming reduces global agricultural production by decreasing cropping frequency and yields. *Nat. Clim. Chang.* **12**, 1016–1023 (2022).
7. Wager, S. & Athey, S. Estimation and Inference of Heterogeneous Treatment Effects using Random Forests. *Journal of the American Statistical Association* **113**, 1228–1242 (2018).
8. Athey, S., Tibshirani, J. & Wager, S. Generalized random forests. *The Annals of Statistics* **47**, 1148–1178 (2019).
9. R Core Team. *R: A Language and Environment for Statistical Computing*. (R Foundation for Statistical Computing, Vienna, Austria, 2023).
10. Hijmans, R. J. terra: Spatial Data Analysis. (2023).
11. Hijmans, R. J. *Raster: Geographic Data Analysis and Modeling*. (2023).
12. Pebesma, E. Simple Features for R: Standardized Support for Spatial Vector Data. *The R Journal* **10**, 439 (2018).
13. Bergé, L. Efficient estimation of maximum likelihood models with multiple fixed-effects: the R package FENmlm. *CREA Discussion Papers* (2018).

14. Arel-Bundock, V. modelsummary: Data and Model Summaries in R. *Journal of Statistical Software* **103**, 1–23 (2022).

15. Arel-Bundock, V., Greifer, N. & Heiss, A. How to Interpret Statistical Models Using marginaleffects in R and Python. *Journal of Statistical Software*.

16. Dowle, M. & Srinivasan, A. data.table: Extension of `data.frame`. (2023).

17. Wickham, H. *et al.* Welcome to the tidyverse. *Journal of Open Source Software* **4**, 1686 (2019).

18. GDAL/OGR contributors. GDAL/OGR Geospatial Data Abstraction software Library. Open Source Geospatial Foundation https://doi.org/10.5281/zenodo.5884351 (2024).

19. Gorelick, N. *et al.* Google Earth Engine: Planetary-scale geospatial analysis for everyone. *Remote Sensing of Environment* **202**, 18–27 (2017).

20. van Ittersum, M. K. *et al.* Yield gap analysis with local to global relevance—A review. *Field Crops Research* **143**, 4–17 (2013).

21. Gerber, J. S. *et al.* Global spatially explicit yield gap time trends reveal regions at risk of future crop yield stagnation. *Nat Food* **5**, 125–135 (2024).

22. Fuglie, K., Morgan, S. & Jelliffe, J. World Agricultural Production, Resource Use, and Productivity, 1961–2020. http://www.ers.usda.gov/publications/pub-details/?pubid=108649.

23. Ray, D. K. *et al.* Climate change has likely already affected global food production. *PLoS ONE* **14**, e0217148 (2019).

24. van Dijk, M. *et al.* Reducing the maize yield gap in Ethiopia: Decomposition and policy simulation. *Agricultural Systems* **183**, 102828 (2020).

25. Wuepper, D., Le Clech, S., Zilberman, D., Mueller, N. & Finger, R. Countries influence the trade-off between crop yields and nitrogen pollution. *Nat Food* **1**, 713–719 (2020).

26. Ghosh, A. *et al.* Monitoring Sustainable Development Goal Indicator 15.3.1 on Land Degradation Using SEPAL: Examples, Challenges and Prospects. *Land* **13**, 1027 (2024).

27. Mo, L. *et al.* Integrated global assessment of the natural forest carbon potential. *Nature* **624**, 92–101 (2023).

28. Sanderman, J., Hengl, T. & Fiske, G. J. Soil carbon debt of 12,000 years of human land use. *Proc. Natl. Acad. Sci. U.S.A.* **114**, 9575–9580 (2017).

29. Bastin, J.-F. *et al.* The global tree restoration potential. *Science* **365**, 76–79 (2019).

30. Sonneveld, B. G. J. S. & Dent, D. L. How good is GLASOD? *Journal of Environmental Management* **90**, 274–283 (2009).

31. Oldeman, L. R., Hakkeling, R. T. A. & Sombroek, W. G. *World Map of the Status of Human-Induced Soil Degradation: An Explanatory Note, Second Revised Edition*. https://www.isric.org/sites/default/files/isric_report_1990_07.pdf (1991).

32. FAO & ITPS. *Status of the World's Soil Resources (SWSR) – Main Report*. (Food and Agriculture Organization of the United Nations and Intergovernmental Technical Panel on Soils, Rome, Italy, 2015).

33. Intergovernmental Panel On Climate Change. *Climate Change and Land: IPCC Special Report on Climate Change, Desertification, Land Degradation, Sustainable Land Management, Food Security, and Greenhouse Gas Fluxes in Terrestrial Ecosystems*. (Cambridge University Press, 2022). doi:10.1017/9781009157988.

34. Olsson, L. *et al.* Land Degradation. in *Climate Change and Land: an IPCC special report on climate change, desertification, land degradation, sustainable land management, food security, and greenhouse gas fluxes in terrestrial ecosystems* (eds. Shukla, P. R. et al.) (IPCC, 2019).

35. Keller, T. & Or, D. Farm vehicles approaching weights of sauropods exceed safe mechanical limits for soil functioning. *Proc. Natl. Acad. Sci. U.S.A.* **119**, e2117699119 (2022).

36. Zhao, Y., Xie, E., Zhang, X. & Peng, Y. Global topsoil SOC stock from 1981 to 2018 estimated by combining process-based model and space-for-time digital soil mapping. Zenodo https://doi.org/10.5281/zenodo.5040380 (2021).

37. Gruber, A., Scanlon, T., van der Schalie, R., Wagner, W. & Dorigo, W. Evolution of the ESA CCI Soil Moisture climate data records and their underlying merging methodology. *Earth System Science Data* **11**, 717–739 (2019).

38. Dorigo, W. *et al.* ESA CCI Soil Moisture for improved Earth system understanding: State-of-the art and future directions. *Remote Sensing of Environment* **203**, 185–215 (2017).

39. Preimesberger, W., Scanlon, T., Su, C.-H., Gruber, A. & Dorigo, W. Homogenization of Structural Breaks in the Global ESA CCI Soil Moisture Multisatellite Climate Data Record. *IEEE Transactions on Geoscience and Remote Sensing* **59**, 2845–2862 (2021).

40. Lawrence, D. & Vandecar, K. Effects of tropical deforestation on climate and agriculture. *Nature Clim Change* **5**, 27–36 (2015).

41. Grosset-Touba, F., Papp, A. & Taylor, C. Rain follows the forest: Land use policy, climate change, and adaptation. SSRN Scholarly Paper at https://doi.org/10.2139/ssrn.4333147 (2024).

42. Carswell, A. M. *et al.* Agricultural practices can threaten soil resilience through changing feedback loops. *npj Sustain. Agric.* **3**, 56 (2025).

43. Prăvălie, R. *et al.* A unifying modelling of multiple land degradation pathways in Europe. *Nat Commun* **15**, 3862 (2024).

44. Chappell, A., Baldock, J. & Sanderman, J. The global significance of omitting soil erosion from soil organic carbon cycling schemes. *Nature Clim Change* **6**, 187–191 (2016).

45. Obalum, S. E., Chibuike, G. U., Peth, S. & Ouyang, Y. Soil organic matter as sole indicator of soil degradation. *Environ Monit Assess* **189**, 176 (2017).

46. Amundson, R. *et al.* Soil and human security in the 21st century. *Science* **348**, 1261071 (2015).

47. FAO. *The State of the World's Land and Water Resources for Food and Agriculture – Systems at Breaking Point. Main Report*. (FAO, Rome, 2022). doi:10.4060/cb9910en.

48. Pimentel, D. & Burgess, M. Soil Erosion Threatens Food Production. *Agriculture* **3**, 443–463 (2013).

49. Lal, R. Digging deeper: A holistic perspective of factors affecting soil organic carbon sequestration in agroecosystems. *Global Change Biology* **24**, 3285–3301 (2018).

50. Pimentel, D. *et al.* World Agriculture and Soil Erosion. *BioScience* **37**, 277–283 (1987).

51. Zhang, Y., Hartemink, A. E., Vanwalleghem, T., Bonfatti, B. R. & Moen, S. Climate and land use changes explain variation in the A horizon and soil thickness in the United States. *Commun Earth Environ* **5**, 129 (2024).

52. Six, J., Conant, R. T., Paul, E. A. & Paustian, K. Stabilization mechanisms of soil organic matter: Implications for C-saturation of soils. *Plant and Soil* **241**, 155–176 (2002).

53. Rawls, W. J., Pachepsky, Y. A., Ritchie, J. C., Sobecki, T. M. & Bloodworth, H. Effect of soil organic carbon on soil water retention. *Geoderma* **116**, 61–76 (2003).

54. Fu, Z. *et al.* Land use effects on soil hydraulic properties and the contribution of soil organic carbon. *Journal of Hydrology* **602**, 126741 (2021).

55. Shaheb, M. R., Venkatesh, R. & Shearer, S. A. A Review on the Effect of Soil Compaction and its Management for Sustainable Crop Production. *J. Biosyst. Eng.* **46**, 417–439 (2021).

56. Correa, J., Postma, J. A., Watt, M. & Wojciechowski, T. Soil compaction and the architectural plasticity of root systems. *J Exp Bot* **70**, 6019–6034 (2019).

57. Nawaz, M. F., Bourrié, G. & Trolard, F. Soil compaction impact and modelling. A review. *Agron. Sustain. Dev.* **33**, 291–309 (2013).

58. Shah, A. N. *et al.* Soil compaction effects on soil health and cropproductivity: an overview. *Environ Sci Pollut Res* **24**, 10056–10067 (2017).

59. Obour, P. B. & Ugarte, C. M. A meta-analysis of the impact of traffic-induced compaction on soil physical properties and grain yield. *Soil and Tillage Research* **211**, 105019 (2021).

60. Chen, X. & Hu, Q. Groundwater influences on soil moisture and surface evaporation. *Journal of Hydrology* **297**, 285–300 (2004).

61. Zhao, S. *et al.* Impact of deeper groundwater depth on vegetation and soil in semi-arid region of eastern China. *Front. Plant Sci.* **14**, (2023).

62. Coppus, R. *The Global Distribution of Human-Induced Land Degradation and Areas at Risk*. https://doi.org/10.4060/cc2843en (2023) doi:10.4060/cc2843en.

63. World Bank. *World Development Report 2008: Agricultural Development*. (2008).

64. Famiglietti, J. S. The global groundwater crisis. *Nature Clim Change* **4**, 945–948 (2014).

65. Scanlon, B. R. *et al.* Groundwater depletion and sustainability of irrigation in the US High Plains and Central Valley. *Proceedings of the National Academy of Sciences* **109**, 9320–9325 (2012).

66. Ledo, A. *et al.* Changes in soil organic carbon under perennial crops. *Global Change Biology* **26**, 4158–4168 (2020).

67. Panagos, P. *et al.* Estimating the soil erosion cover-management factor at the European scale. *Land Use Policy* **48**, 38–50 (2015).

68. Von Jeetze, P. J. *et al.* Projected landscape-scale repercussions of global action for climate and biodiversity protection. *Nat Commun* **14**, 2515 (2023).

69. Borrelli, P. *et al.* An assessment of the global impact of 21st century land use change on soil erosion. *Nat Commun* **8**, 2013 (2017).

70. DiMiceli, C., Sohlberg, R. & Townshend, J. MODIS/Terra Vegetation Continuous Fields Yearly L3 Global 250m SIN Grid V061. (2022).

71. Poggio, L. *et al.* SoilGrids 2.0: producing soil information for the globe with quantified spatial uncertainty. *SOIL* **7**, 217–240 (2021).

72. Xie, E. *et al.* Integration of a process-based model into the digital soil mapping improves the space-time soil organic carbon modelling in intensively human-impacted area. *Geoderma* **409**, 115599 (2022).

73. Hansen, M. C. *et al.* High-Resolution Global Maps of 21st-Century Forest Cover Change. *Science* **342**, 850–853 (2013).

74. Fick, S. E. & Hijmans, R. J. WorldClim 2: new 1-km spatial resolution climate surfaces for global land areas. *Intl Journal of Climatology* **37**, 4302–4315 (2017).

75. Abatzoglou, J. T., Dobrowski, S. Z., Parks, S. A. & Hegewisch, K. C. TerraClimate, a high-resolution global dataset of monthly climate and climatic water balance from 1958–2015. *Sci Data* **5**, 170191 (2018).

76. Danielson, J. J. & Gesch, D. B. *Global Multi-Resolution Terrain Elevation Data 2010 (GMTED2010)*. 26 (2011).

77. Amatulli, G., McInerney, D., Sethi, T., Strobl, P. & Domisch, S. Geomorpho90m, empirical evaluation and accuracy assessment of global high-resolution geomorphometric layers. *Sci Data* **7**, 162 (2020).

78. Hengl, T. & Nauman, T. Predicted USDA soil great groups at 250 m (probabilities). Zenodo https://doi.org/10.5281/zenodo.3528062 (2018).

79. Tian, H. *et al.* History of anthropogenic Nitrogen inputs (HaNi) to the terrestrial biosphere: a 5 arcmin resolution annual dataset from 1860 to 2019. *Earth Syst. Sci. Data* **14**, 4551–4568 (2022).

80. McDowell, R. W., Noble, A., Pletnyakov, P. & Haygarth, P. M. A Global Database of Soil Plant Available Phosphorus. *Sci Data* **10**, 125 (2023).

81. Siebert, S. *et al.* A global data set of the extent of irrigated land from 1900 to 2005. https://doi.org/10.13019/M20599 (2015) doi:10.13019/M20599.

82. Mehta, P. *et al.* Half of twenty-first century global irrigation expansion has been in water-stressed regions. *Nat Water* **2**, 254–261 (2024).

83. Maggi, F., Tang, F. H. M., La Cecilia, D. & McBratney, A. PEST-CHEMGRIDS, global gridded maps of the top 20 crop-specific pesticide application rates from 2015 to 2025. *Sci Data* **6**, 170 (2019).

84. World Bank. Employment in agriculture (% of total employment) (modeled ILO estimate). World Bank Open Data (2022).

85. U.S. Department of Agriculture Economic Research Service. International Agricultural Productivity. (2023).

86. Adamopoulos, T. & Restuccia, D. Geography and Agricultural Productivity: Cross-Country Evidence from Micro Plot-Level Data. *The Review of Economic Studies* **89**, 1629–1653 (2022).

87. Fischer, R., Byerlee, D. & Edmeades, G. O. Can technology deliver on the yield challenge to 2050. in *Expert meeting on how to feed the world in 2050* 46, 389–462 (FAO, Rome, 2009).

88. Kummu, M., Taka, M. & Guillaume, J. H. A. Gridded global datasets for Gross Domestic Product and Human Development Index over 1990–2015. *Sci Data* **5**, 180004 (2018).

89. World Bank. Agriculture, forestry, and fishing, value added (% of GDP). World Bank Open Data (2022).

90. World Bank. Access to electricity, rural (% of rural population). World Bank Open Data (2022).

91. World Bank. Mobile cellular subscriptions (per 100 people). World Bank Open Data (2022).

92. Browne, C., Di Battista, A., Geiger, T. & Gutknecht, T. The Executive Opinion Survey: The Voice of the Business Community. in *The Global Competitiveness Report 2014–2015* (eds. Bilbao-Osorio, B. & others) (World Economic Forum, 2014).

93. Standaert, S. Divining the level of corruption: A Bayesian state-space approach. *Journal of Comparative Economics* **43**, 782–803 (2015).

94. Wuepper, D. *et al.* Agri-environmental policies from 1960 to 2022. *Nat Food* **5**, 323–331 (2024).

95. Barbier, E. B. & Hochard, J. P. Land degradation and poverty. *Nat Sustain* **1**, 623–631 (2018).

96. Breiman, L. Random Forests. *Machine Learning* **45**, 5–32 (2001).

97. Tibshirani, J., Athey, S., Sverdrup, E. & Wager, S. *Grf: Generalized Random Forests*. (2023).

98. FAO. FAOSTAT: Food Balances (2010-). (2024).

99. FAO. FAOSTAT: Producer Prices. (2024).

100. Pelletier, J. D. *et al.* A gridded global data set of soil, intact regolith, and sedimentary deposit thicknesses for regional and global land surface modeling. *J Adv Model Earth Syst* **8**, 41–65 (2016).

101. Fan, Y., Li, H. & Miguez-Macho, G. Global Patterns of Groundwater Table Depth. *Science* **339**, 940–943 (2013).

102. Lesiv, M. *et al.* Estimating the global distribution of field size using crowdsourcing. *Global Change Biology* **25**, 174–186 (2019).

103. FAO. FAOSTAT: Government Expenditure. (2024).

104. Lawson, R. & Murphy, R. Economic Freedom Dataset, published in Economic Freedom of the World: 2023 Annual Report. (2023).

105. Wuepper, D., Wang, H., Schlenker, W., Jain, M. & Finger, R. *Institutions and Global Crop Yields*. https://www.nber.org/papers/w31426 (2023) doi:10.3386/w31426.

106. Meijer, J. R., Huijbregts, M. A. J., Schotten, K. C. G. J. & Schipper, A. M. Global patterns of current and future road infrastructure. *Environ. Res. Lett.* **13**, 064006 (2018).

107. Weiss, D. J. *et al.* A global map of travel time to cities to assess inequalities in accessibility in 2015. *Nature* **553**, 333–336 (2018).

108. Shamdasani, Y. Rural road infrastructure & agricultural production: Evidence from India. *Journal of Development Economics* **152**, 102686 (2021).

109. Wuepper, D. Does culture affect soil erosion? Empirical evidence from Europe. *European Review of Agricultural Economics* **47**, 619–653 (2020).

110. Kim, J. H. Multicollinearity and misleading statistical results. *Korean J Anesthesiol* **72**, 558–569 (2019).

111. Panagos, P. *et al.* Cost of agricultural productivity loss due to soil erosion in the European Union: From direct cost evaluation approaches to the use of macroeconomic models. *Land Degradation & Development* **29**, 471–484 (2018).

112. Carr, T. W., Balkovič, J., Dodds, P. E., Folberth, C. & Skalský, R. The impact of water erosion on global maize and wheat productivity. *Agriculture, Ecosystems & Environment* **322**, 107655 (2021).

113. den Biggelaar, C., Lal, R., Wiebe, K. & Breneman, V. The Global Impact Of Soil Erosion On Productivity I: Absolute and Relative Erosion-induced Yield Losses. in *Advances in Agronomy* vol. 81 1–48 (Academic Press, 2003).

114. Hassani, A., Azapagic, A. & Shokri, N. Predicting long-term dynamics of soil salinity and sodicity on a global scale. *Proc. Natl. Acad. Sci. U.S.A.* **117**, 33017–33027 (2020).

115. Van der Esch, S. *et al. Exploring Future Changes in Land Use and Land Condition and the Impacts on Food, Water, Climate Change, and Biodiversity: Scenarios for the Global Land Outlook*. (2017).

116. Jiang, K. *et al.* Global land degradation hotspots based on multiple methods and indicators. *Ecological Indicators* **158**, 111462 (2024).

117. Gomiero, T. Soil Degradation, Land Scarcity and Food Security: Reviewing a Complex Challenge. *Sustainability* **8**, 281 (2016).

118. Rickson, R. J. *et al.* Input constraints to food production: the impact of soil degradation. *Food Sec.* **7**, 351–364 (2015).

119. Hassani, A., Smith, P. & Shokri, N. Negative correlation between soil salinity and soil organic carbon variability. *Proceedings of the National Academy of Sciences* **121**, e2317332121 (2024).

120. Palm, C., Blanco-Canqui, H., DeClerck, F., Gatere, L. & Grace, P. Conservation agriculture and ecosystem services: An overview. *Agriculture, Ecosystems & Environment* **187**, 87–105 (2014).

121. Iseman, T. & Miralles-Wilhelm, F. *Nature-Based Solutions in Agriculture – The Case and Pathway for Adoption*. (FAO and The Nature Conservancy, Virginia, 2021). doi:10.4060/cb3141en.

122. Chrisendo, D., Piipponen, J., Heino, M. & Kummu, M. Socioeconomic factors of global food loss. *Agric & Food Secur* **12**, 23 (2023).
