# Supplementary material for: Land degradation is associated with larger crop yield gaps across global croplands
Source: Nat Food. 2026 Jul 22;7(7):678–87. doi: 10.1038/s43016-026-01382-5 (PMC13391347; doi:10.1038/s43016-026-01382-5)
Supplement: Supplementary file 2 — Reporting Summary [file 43016_2026_1382_MOESM2_ESM.pdf]

nature portfolio

Corresponding author(s): Hadi Hadi; h.hadi@ilr.uni-bonn.de

Last updated by author(s): May 27, 2026

Reporting Summary

Nature Portfolio wishes to improve the reproducibility of the work that we publish. This form provides structure for consistency and transparency in reporting. For further information on Nature Portfolio policies, see our Editorial Policies and the Editorial Policy Checklist.

Statistics

For all statistical analyses, confirm that the following items are present in the figure legend, table legend, main text, or Methods section.

n/a	Confirmed	
☐	☒	The exact sample size (*n*) for each experimental group/condition, given as a discrete number and unit of measurement
☐	☒	A statement on whether measurements were taken from distinct samples or whether the same sample was measured repeatedly
☐	☒	The statistical test(s) used AND whether they are one- or two-sided<br>*Only common tests should be described solely by name; describe more complex techniques in the Methods section.*
☐	☒	A description of all covariates tested
☐	☒	A description of any assumptions or corrections, such as tests of normality and adjustment for multiple comparisons
☐	☒	A full description of the statistical parameters including central tendency (e.g. means) or other basic estimates (e.g. regression coefficient) AND variation (e.g. standard deviation) or associated estimates of uncertainty (e.g. confidence intervals)
☐	☒	For null hypothesis testing, the test statistic (e.g. F, t, r) with confidence intervals, effect sizes, degrees of freedom and P value noted<br>*Give P values as exact values whenever suitable.*
☒	☐	For Bayesian analysis, information on the choice of priors and Markov chain Monte Carlo settings
☒	☐	For hierarchical and complex designs, identification of the appropriate level for tests and full reporting of outcomes
☒	☐	Estimates of effect sizes (e.g. Cohen's *d*, Pearson's *r*), indicating how they were calculated

Our web collection on statistics for biologists contains articles on many of the points above.

Software and code

Policy information about availability of computer code

Data collection	All input datasets were obtained from publicly available sources, as described in the Methods and Supplementary Table 4. Spatial data compilation, harmonization and extraction were performed using Google Earth Engine and R version 4.5.2 (R Foundation for Statistical Computing). Geospatial data processing in R used the packages sf v1.0.19, raster v3.6.31, terra v1.8.29, exactextractr v0.10.1 and fasterize v1.1.0. Data wrangling used dplyr v1.1.4, readr v2.1.5 and data.table v1.18.0. Google Earth Engine is a cloud-based geospatial analysis platform and does not provide a fixed package-style version number for the platform used in this study.
Data analysis	Statistical analyses were performed in R version 4.5.2. Regression models were estimated using fixest v0.13.2. Marginal effects and average slopes were computed using marginaleffects v0.32.0. Model summaries and coefficient extraction used broom v1.0.7 and modelsummary v2.5.0. Causal forest analyses were performed using grf v2.6.1. Figure and map generation used ggplot2 v4.0.2, tidyterra v0.7.2, cowplot v1.1.3, colorspace v2.1.1, classInt v0.4.11, tmap v4.2 and tmaptools v3.3. Country-code harmonization used countrycode v1.6.1. Custom R scripts and Google Earth Engine workflows used for data processing, analysis and figure generation are deposited with the replication materials associated with the article.

For manuscripts utilizing custom algorithms or software that are central to the research but not yet described in published literature, software must be made available to editors and reviewers. We strongly encourage code deposition in a community repository (e.g. GitHub). See the Nature Portfolio guidelines for submitting code & software for further information.

Data

Policy information about availability of data

All manuscripts must include a data availability statement. This statement should provide the following information, where applicable:

- Accession codes, unique identifiers, or web links for publicly available datasets
- A description of any restrictions on data availability
- For clinical datasets or third party data, please ensure that the statement adheres to our policy

All input datasets used in this study were obtained from publicly available sources. The datasets, access links and citations are described in the Methods section and listed in Supplementary Table 4. The processed analysis-ready data, source data underlying the figures and tables, and replication materials generated during this study are deposited in Zenodo (10.5281/zenodo.20214047) with the article replication package. There are no restrictions on access to the processed data generated by this study. Users of the original third-party datasets should consult the cited source providers for the applicable terms of use and licensing conditions.

Research involving human participants, their data, or biological material

Policy information about studies with human participants or human data. See also policy information about sex, gender (identity/presentation), and sexual orientation and race, ethnicity and racism.

Reporting on sex and gender	Not applicable.
Reporting on race, ethnicity, or other socially relevant groupings	Not applicable.
Population characteristics	Not applicable.
Recruitment	Not applicable.
Ethics oversight	Not applicable.

Note that full information on the approval of the study protocol must also be provided in the manuscript.

Field-specific reporting

Please select the one below that is the best fit for your research. If you are not sure, read the appropriate sections before making your selection.

☐ Life sciences ☐ Behavioural & social sciences ☒ Ecological, evolutionary & environmental sciences

For a reference copy of the document with all sections, see nature.com/documents/nr-reporting-summary-flat.pdf

Ecological, evolutionary & environmental sciences study design

All studies must disclose on these points even when the disclosure is negative.

Study description	This study used observational geospatial datasets sourced from satellite-derived data products, census data, and secondary data sources, to assess the link between land degradation and crop yields globally, and where global impact hotspots are to be found. To achieve this, we first compiled dataset consisting of various high-resolution global maps of land degradation, crop yield gaps, and an extensive set of control variables. Then, we carried out an analysis using regression models and machine learning methods to explore the relationship between crop yield gaps and land degradation, including multiple sensitivity analyses. Finally, we calculated the estimated loss of food production, calories, and revenues.
Research sample	We analyzed the full population of global cropland areas as identified in the secondary datasets (N = 405,084 grid cells at 10-km resolution).
Sampling strategy	No sampling was performed in the analysis.
Data collection	We compiled datasets from publicly available sources, consisting of various high-resolution global maps of land degradation, crop yield gaps, and an extensive set of natural environmental, agricultural management, as well as socio-economic and institutional control variables.
Timing and spatial scale	The common spatial scale of the analysis is 10-km x 10-km. The native spatial scales of the input datasets span from 90-m to country level. The land degradation data were constructed as the difference between current land conditions (circa 2010) and historical (natural) conditions, or as long-term differences in land conditions (between circa 1980 and circa 2010). The timing of the target variable of interest namely crop yield gaps is circa 2010, and therefore for all the control variables, when available, data for circa 2010 were used.

Data exclusions	No data were excluded from the analysis.
Reproducibility	Within the manuscript, we clearly stated all the data and the steps taken to ensure the reproducibility of the study, identifying all software languages and packages. We analyzed the entire population data (global croplands as identified in the secondary data); there is no experiment to be repeated.
Randomization	In our observational-based analysis of the link between land degradation and crop yield gaps, we controlled for natural environmental (incl. climate, soil, terrain characteristics), agricultural management (incl. fertilizer use, irrigation, labor, mechanization), as well as socio-economic and institutional factors (incl. agricultural share in GDP, property rights, corruption). We did not perform a randomized control trial.
Blinding	Blinding refers to the concealment of group allocation from one or more individuals involved in a study, and is thus not relevant to our study which was desktop-based

Did the study involve field work? ☐ Yes ☒ No

Reporting for specific materials, systems and methods

We require information from authors about some types of materials, experimental systems and methods used in many studies. Here, indicate whether each material, system or method listed is relevant to your study. If you are not sure if a list item applies to your research, read the appropriate section before selecting a response.

Materials & experimental systems

n/a	Involved in the study
☒	☐ Antibodies
☒	☐ Eukaryotic cell lines
☒	☐ Palaeontology and archaeology
☒	☐ Animals and other organisms
☒	☐ Clinical data
☒	☐ Dual use research of concern
☒	☐ Plants

Methods

n/a	Involved in the study
☒	☐ ChIP-seq
☒	☐ Flow cytometry
☒	☐ MRI-based neuroimaging

Plants

Seed stocks	Not applicable.
Novel plant genotypes	Not applicable.
Authentication	Not applicable.
